# Proceedings of the Andalas International Public Health Conference 2017: Padang, West Sumatera Province, Indonesia. 06-07 September 2017

**DOI:** 10.1186/s12889-017-4877-4

**Published:** 2017-11-30

## O1 An outbreak of typhoid fever at secondary school of Magelang district, Indonesia in 2016

### Nasir Ahmad^1^, Adi Isworo^2^, Chrisnaety Indriani Silaban^1^

#### ^1^Field Epidemiology Training Programs, Gadjah Mada University, Yogyakarta 55281, Indonesia; ^2^Polytechnic of Health, Indonesian Ministry of Health, Semarang, 50268, Indonesia

##### **Correspondence:** Nasir Ahmad (nasirahmad3443@gmail.com)

**Background**

On October 15th, 2016, Magelang District Health Office (DHO) received a report from a school that some students had typhoid fever. Outbreak investigation was done to know the magnitude of outbreak, the cause, and describe the outbreak.

**Materials and methods**

This research was a descriptive epidemiology study with active case finding to find additional cases. Data were collected by interview using standard questionnaire. The researchers took sample of clean water, drinking water and blood in school. Water sample and blood were sent to laboratory.

**Results**

Total number of cases were 150 students. The highest attack rate was found among the first year students 139 per 381 (36.48%) and 72 per 359 females (20.1%). The onset of disease started from 28 September to 26 October. There was a bathing activity in the river at on 26 September that the students attended which was followed by eating together without washing hand with soap. IgM was positive in 51 students and water sample was negative for *Salmonella typhi*.

**Conclusions**

There was an outbreak of typhoid fever with 150 cases in school on September to November 2016. The outbreak was caused by bathing activity in the river and not washing hand before eating.

## O2 Estimation of TB burden on districts and cities level in West Sumatra Province, Indonesia

### Defriman Djafri (defrimandjafri@fkm.unand.ac.id)

#### Department of Epidemiology & Biostatistics, Faculty of Public Health, Andalas University, Padang, 25147, Indonesia

**Background**

Indonesia is among the top highest TB burden globally with more than 1 million cases in a year and 690.000 not reported second highest TB burden globally. There were differences in the prevalence and incidence. There were differences in the prevalence and the incidence among regional and districts/cities. There was considerable variation between provinces and between districts / cities in the case of notification per 100.00 population. An estimate of burden of disease is required for planning and budgeting. The objective study was assessing estimation of TB burden districts and cities level in West Sumatra Province, Indonesia.

**Materials and methods**

The estimation was conducted by using the results from TB prevalence surveys. Incidence was estimated by using measurements from national surveys of the prevalence of TB disease which were combined with estimates of the duration of disease. Incidence was estimated as the prevalence of TB was divided by the average duration of disease assuming epidemic equilibrium.

**Results**

There was a marked difference in the estimation of TB burden between cities and districts. The estimated burden of TB in cities was higher than in districts. In the city, the incidence was estimated 650-670 per 100.000 population and the district was estimated 425-500 per 100.000 population. Several possible risk factors differentiated the allocation of TB Burden each city and district.

**Conclusions**

High quality prevalence survey is needed to estimate TB incidence in order to allocate the national TB burden to each city and district in Indonesia.

## O3 Self-medication practice among senior high school students in Padang, Indonesia

### Dedy Almasdy^1^, Dian Ayu Juwita^1^, Ika Rosmayanti^1^, Nina Kurniasih^2^

#### ^1^Faculty of Pharmacy, Andalas University, Padang, 25163, Indonesia; ^2^Dr. Rasidin Hospital, Padang, 25159, Indonesia

##### **Correspondence:** Dedy Almasdy (dedyalmasdy@gmail.com)

**Background**

Self-medication is an act of people to select and to use medicines - both modern and traditional - for themselves to treat illness or sign and symptoms of illness without consulting to a professional health care. This practice is commonly used in the world. The study aimed to know the description of self-medication practice among senior high school students in Padang City.

**Materials and methods**

The study was a cross-sectional study using a questionnaire as data collection instrument. The samples were taken by using stratified random sampling technique among senior high school students in Padang.

**Results**

The results showed that the practice of self-medication was mostly done by female students (63.2%) and by mathematic and natural sciences students (59.5%). The main reason was the assumption that they did not suffer serious illness (44.4%). Sources of information on self-medication practice were mainly parents, relatives, or friends (63.8%). The medicine was mostly obtained from pharmacies (67.5%). The most frequent used drug was analgesic or antipyretic (54.6%), while few of respondents used antibiotics (4.0%). Most of the students used two or more medicines (64.1%). Furthermore, most of them did not read the brochure before using the medicine (62.0%).

**Conclusions**

Self-medication was mostly done by female students. The most frequent used drug was analgesic or antipyretic. Students’ reference for self-medication were their surrounding people including parents, relatives, or friends.

## O4 Spatial analysis of Dengue Hemorrhagic Fever (DHF) in Pesisir Selatan Regency, Indonesia

### Yeffi Masnarivan, Yoko Masna Rivan M

#### STIKes Prima Nusantara, Bukittinggi, 26122, Indonesia

##### **Correspondence:** Yeffi Masnarivan (yeffimasnarivan@gmail.com)

**Background**

Dengue Hemorrhagic Fever (DHF) is a contagious disease that is still a national problem in the field of public health. Nowadays, DHF disease is likely to increase and spread widely. This study was aimed to describe the incidence of DHF from 2013 to 2016 through spatial analysis in Pesisir Selatan Regency.

**Materials and methods**

The study was a times series study. The data used were secondary data of DHF cases in Pesisir Selatan Regency from 2013 to 2016.

**Results**

The results showed that, spatially, the highest DHF incidence rate in South Pesisir Regency was found in coverage area of Salido Health Centre. There were 233 DHF cases per 100.000 population. The lowest incidence was found in coverage area of Tanjung Makmur Health Centre with 19 DHF cases per 100.000 population.

**Conclusions**

Generally, all working area of health centers in Pesisir Selatan Regency are classified as dengue-endemic areas. It is suggested to the public to increase their participation in prevention and control of Dengue fever such as Mosquito Nest Eradication program. Health promotion should be improved such as community counseling in public health centers with high incidence of DHF.

## O5 Socioeconomic factors and community-acquired pneumonia prevalence in Indonesian children: a multilevel analysis

### R. Machmud^1^, A. Bakhtiar^2^, P. Loh^3^, I. Ariawan^4^, R. Agustina^5^

#### ^1^Department of Public Health, Faculty of Medicine, Andalas University, Padang, 25147, Indonesia; ^2^Department of Health Public Policy and Administration, Faculty of Public Health, University of Indonesia, Padang, 25163, Indonesia; ^3^Department of Epidemiology, Harvard School of Public Health, Cambridge, MA, 02115, USA; ^4^Department of Biostatistics, Faculty of Public Health, University of Indonesia, Padang, 25147, Indonesia; ^5^Department of Nutrition, Faculty of Medicine, University of Indonesia, Padang, 25147, Indonesia

##### R. Machmud (rizandamachmud@fk.unand.ac.id)

**Background**

We examined associations between the district-level human development index (HDI) and household-level socioeconomic status scores and the prevalence of community-acquired pneumonia among children under-5 years in Indonesia.

**Materials and methods**

The study used data from the University of Indonesia’s Benefit Evaluation Study (BES) II to analyze 7,170 children from 10,900 households across 27 districts from 7 provinces in Indonesia.

Multilevel logistic regression was applied to determine the Intracluster Correlation Coefficient (ICC) and the contribution of district-level HDI and household-level socioeconomic status indicators on the prevalence of pneumonia among under-five children.

**Results**

The overall prevalence of pneumonia of these children was 4.9% (CI 95% 3.34 - 6.57). This finding showed that there was association of the district-level HDI status scores and no association between the household-level socioeconomic status scores in the prevalence of pneumonia. The prevalence of pneumonia in the low-HDI district was significantly higher (9.3%) than high-HDI district (4.6%). The prevalence of pneumonia in wealthy households were similar (6.1%) to poor households (6.6%).

**Conclusions**

In Indonesia, districts with low HDI scores have higher rates of pneumonia among under-five children compared to those with high HDI scores. Therefore, children from these districts may be at an increased risk of pneumonia.

## O6 Evaluation of malaria migration surveillance system in Magelang District in 2015

### Nasir Ahmad^1^, Adi Isworo^2^, Chrisnaety Indriani Silaban^1^

#### ^1^Field Epidemiology Training Programs, Gadjah Mada University, Yogyakarta 55281, Indonesia; ^2^Polytechnic of Health, Indonesian Ministry of Health, Semarang, 50268, Indonesia

##### **Correspondence:** Nasir Ahmad (nasirahmad3443@gmail.com)

**Background**

Magelang District was certified for reaching elimination of malaria in April 2014, but an epidemic signal was re-established in April 2015. Earlier import cases were obtained in March. Evaluation of the malaria migration surveillance system was conducted in terms of the implementation process and obstacles of the malaria migration surveillance system in Magelang District.

**Materials and methods**

This study was a descriptive research. The subjects were 15 village officers. The data were collected by using interview and observation on surveillance data collection and data processing.

**Results**

The community awareness in reporting the migrants was lacking. The barrier was during blood donation when sometimes the immigrants refused to take a blood test.

**Conclusions**

Malaria migration surveillance system in Magelang District hampered by some barriers. The barrier on data collection was lack of awareness among the society to report new migrants and migrants refused to take blood test. It is expected that the head of the village make some regulations regarding society participation in malaria migration surveillance.

## O7 The effect of family assistance to improve the quality of life of DM Type 2 patients in Makassar, Indonesia

### Ridwan Amiruddin^1^, Ansariadi^1^, Sri Syatriani^2^, Nurhaedar Djaffar^3^

#### ^1^Department of Epidemiology, Faculty of Public Health, Hasanuddin University, Makassar, 90245, Indonesia; ^2^Department of Biomedic, Faculty of Medical Sciences Hasanuddin University, Makassar, 90245, Indonesia; ^3^Department Nutrition, Faculty Public Health, Faculty of Hasanuddin University, Makassar, 90245, Indonesia

##### **Correspondence:** Ridwan Amiruddin (ridwan.amiruddin@gmail.com)

**Background**

The prevalence of diabetes mellitus in Indonesia over the past five years has increased. Indonesian Basic Health Research conducted in 2010 showed that the prevalence of diabetes mellitus increased by 1.5%, and 2.1% in 2013. In 2014, it increased by 5.8% and the number of DM patients increased to 9.1 million in 2015. The aim of this research was to know the influence of family assistance toward the improvement of quality of life among DM type 2 patients in Makassar City, Indonesia.

**Materials and methods**

This study used a non-randomized pre- and post-test control group design. The samples were 80 patients of DM type 2 in coastal area of Makassar City. The data were analyzed by using paired t-test.

**Results**

The results showed that family assistance could improve the quality of life of patients with DM type 2. There was a significant difference between the quality of life of DM Type 2 patients before and after intervention. The number of DM type 2 patients whose quality of life was less good decreased with the mean difference of quality of life scores was 21.77.

**Conclusions**

Family assistance affected the improvement of quality of life among DM type 2 patients in Makassar city.

## O8 Smoking habit, physical activity and hypertension in Indonesian rural area

### Rian Diana^1^, Ali Khomsan^2^, Naufal Muharam Nurdin^2^, Faisal Anwar^2^, Hadi Riyadi^2^

#### ^1^Department of Nutrition, Faculty of Public Health, Universitas Airlangga, Surabaya, 60115, Indonesia; ^2^Department of Community Nutrition, Faculty of Human Ecology, Bogor Agricultural University, Bogor, 16680, Indonesia

##### **Correspondence:** Rian Diana (rian.diana@fkm.unair.ac.id)

**Background**

Hypertension is increasingly prevalent in Indonesia particularly among middle aged population. The purpose of this study was to examine the associations between smoking habit, physical activity, and hypertension among middle aged people in rural area.

**Materials and methods**

This cross sectional study involved 224 subjects aged 45-59 years old and conducted in Cianjur District, West Java in 2014. Data on blood pressure was measured by an automatic blood pressure monitor. Data characteristics of the sample and smoking habit were collected through interview with structured questionnaire. Physical activity (PA) was assessed by 24-hour physical activity recall. Chi Square was applied to analyze the associations between smoking habit, physical activity and hypertension.

**Results**

Most of the subjects (92.9%) had low education level. Majority of the subjects worked as farm laborer (43.3%) and 46.4% of subjects had smoking habit. They consumed 8.1 cigarette/day and 90% of them were men. Most of the subjects (72.1%) had intention to stop smoking and 79.8% realized that smoking endangers their health but 60.6% of them smoke the similar number of cigarette compared to last year. Based on a 24-hour PA recall during a working day, 38.8% had a light PA, 37.5% had a heavy PA and 23.7% had moderate PA. This study found that 49.1% of subjects had hypertension, 35.7% had prehypertension, and 15.2% had a normal blood pressure. Chi square showed that there was a significant association between smoking habit and physical activity with hypertension.

**Conclusions**

High prevalence of hypertension was associated with smoking habit and low physical activity. This implied that the importance of not smoking and had a regular physical activity were good for decreasing the prevalence of hypertension.

## O9 Toddlers, children, and referred patients as predictors for Dengue Shock Syndrome (DSS) in Semarang City, Central Java Province, Indonesia

### Yudi Pradipta^1^, Ida Safitri Laksanawati^2^, Dibyo Pramono^3^

#### ^1^Faculty of Public Health, Andalas University, Padang, 25147, Indonesia; ^2^Department of Children Health, Sardjito Hospital, Yogyakarta, 55284 Indonesia; ^3^Department of Preventive & Community Dentistry, Faculty of Dentistry, University of Gadjah Mada, Yogyakarta, 55281, Indonesia

##### **Correspondence:** Yudi Pradipta (yudipradipta@yahoo.co.id)

**Background**

Semarang city is an endemic area of dengue in Indonesia with incidence rate at 92.43 per 100.000 in 2014. Most fatal cases were caused by severe dengue signed by shock syndrome. The severity of dengue was differently related to social determinants including individual factors, socioeconomics, and health system. This study aimed to investigate the social determinants that related to DSS in Semarang City.

**Materials and methods**

A case-control study was conducted in February until March 2016. Cases were dengue patients with shock syndrome diagnosed by the clinician in hospital, and controls were dengue patients without shock syndrome. Sampling was done based on dengue surveillance data from district health office regarding most recent DSS and non-DSS hospitalized patients. Participants were interviewed by using structured questionnaire. Data were analyzed by using chi-square test and logistic regression with 95% CI.

**Results**

There were 73 cases and 73 controls. Most of shock syndrome patients were children (64.38%), female (49.32%), the decision maker in family was low educated (27.40%), admitted to hospital after 4 days of fever (52.05%), and referred from primary health cares (89.04%). Factors related to DSS were under 5 years old (OR=4.02; 95% CI=1.27-12.68), age group 5-18 years old (OR=4.14; 95% CI=1.47-11.61), and referred patients (OR=3.217; 95% CI=1.28-8.05).

**Conclusions**

The community should improve the awareness of shock syndrome and take their children immediately to health services to obtain prompt treatment. Precision diagnosis and the decision made by the clinician to refer the patients rapidly to the hospital were important to reduce the risk of severe dengue.

## O10 Legal aspect on public health protection from tobacco consumption and exposure in Indonesia

### Sukanda Husin (kanda_57@yahoo.com)

#### Faculty of Law, Andalas University, Padang, West Sumatera, 21528, Indonesia

**Background**

Indonesia is the second largest cigarette market in the world and 90 million or 65 of its male population are smokers. Furthermore, 20 million of the smokers are children. This statistic clearly shows that Indonesia faces the devastating health, social, environmental and economic consequences of tobacco consumption or exposure to cigarette smoke. The health, social, environmental and economic effects of cigarette smoke has attracted international attention. With regard with the control of tobacco consumption, the World Health Organization enacts WHO Framework Convention on Tobacco Control (FCTC). The objective of this Convention and its protocols is to protect present and future generations. However, Indonesia does not ratify the Convention yet due to the national interest. This paper is intended to find the reasons why Indonesia does not ratify FCTC and to offer possible legal solution to the problem.

**Materials and methods**

This research is a normative legal research, which is based on literature study.

**Results**

The result of the research is that Indonesia does not yet have the comprehensive law on the restriction of tobacco consumption and distribution. The restriction of tobacco consumption and distribution is still regulated under the Act No. 36 of 2009 concerning Health. The Price and tax measures as the most important strategy to control tobacco is not stipulated under the Act No. 36 of 2009.

**Conclusions**

Based on the research, it can be concluded that Indonesian Government is reluctant to ratify FCTC due to merely economic reasons, notably the states revenue from tobacco, and the income of the farmers and industries are among the factors which are considered by the Government of Indonesia.

## O11 Determinants of maternal mortality in Padang City, Indonesia

### Masrizal, Ainul Mardia Oktiara, Ratno Widoyo

#### Faculty of Public Health, Andalas University, Padang, 21528, Indonesia

##### **Correspondence:** Masrizal (masrizal_khaidir@yahoo.com)

**Background**

Maternal Mortality Rate (MMR) in Padang City increased during the period of 2013 to 2015. The increase of MMR was not regardless of various factors such as health status and healthy behavior. This research aimed to classify and map the sub-districts of Padang City based on the determinants of maternal mortality in Padang.

**Materials and methods**

The study design was descriptive analytic by using secondary data with seven determinants of maternal mortality. Sub-districts were grouped by using cluster analysis. The unit analysis in this study was all sub-districts in Padang.

**Results**

Cluster analysis resulted two sub-groups. The first cluster included sub-districts which have more factors that influence maternal mortality, while the second cluster included sub-districts which have less influencing factors of maternal mortality. Determinants of maternal mortality were percentage of pregnant women at high risk of complication, the percentage of new family planning, the percentage of participant of active family planning, percentage of pregnant women who are carrying out antenatal care for first trimester, and the percentage of pregnant women with tetanus immunization.

**Conclusions**

Percentage of high-risk women, new and active participants in family planning, antenatal care, and having tetanus immunization were the determinant factors of maternal mortality in Padang. It is suggested to the Padang District Health Office to plan maternal health program based on the characteristic area of each sub-district.

## O12 The determinants of Tuberculosis treatment results in referral hospitals of ‘Aisyiyah community TB care program

### Rifqatussa’adah^1^, Sudiyanto Kamso^2^, Purwantyastuti^3^

#### ^1^Department of Public Health, University of YARSI, Jakarta, 10510, Indonesia; ^2^Faculty of Public Health, University of Indonesia, Depok, 16424, Indonesia; ^3^Faculty of Medicine, University of Indonesia, Jakarta, 10430, Indonesia

##### **Correspondence:** Rifqatussa’adah (rifqatussaadah@yarsi.ac.id)

**Background**

Indonesia is predicted to reach 1 million tuberculosis cases per year. Some private hospitals in Indonesia provide community tuberculosis services and also involved in the community activities to support tuberculosis control program. One of the collaborations between private party and community in organizing tuberculosis program is ‘Aisyiyah Community TB Care. This study aimed to identify the determinants of tuberculosis treatment results in referral hospitals of ‘Aisyiyah Community TB Care Program.

**Materials and methods**

The study was conducted from April 2015 to April 2016. As many as 410 adult TB patients were the samples of this study, treated in referral hospitals of ‘Aisyiyah community TB care program including Cempaka Putih Islamic Hospital, Pondok Kopi Islamic Hospital, and Sukapura Islamic Hospital. Data was collected from medical records.

**Results**

The results showed that the most dominant variable affecting tuberculosis treatment outcome was the existence of drug intake supervisor. Patient with drug intake supervisor had better treatment outcome compared to those with no drug intake supervisor after controlled by age variable (OR = 2.3, 95% CI:1.14 to 4.77). Drug intake supervisors in 2013 were the cadres of ‘Aisyiyah (35%) and the patient's family (65%), whereas in 2014, the supervisors were from the relatives of the patients (100%).

**Conclusions**

The existence of drug intake supervisor determined the better outcome of tuberculosis treatment. It is recommended not to choose the PMO from patient’s family. Other people who have got training on tuberculosis treatment are more respected by the patient. However, supervisor activities should be well-monitored during treatment.

## O13 Relationship of immunization status and diphtheria based on geographic information system in Padang, Indonesia

### Masrizal, Febi Damisti Ramadhani

#### Public Health, Andalas University, Padang, West Sumatra, 21528, Indonesia

##### **Correspondence:** Masrizal (masrizal_khaidir@yahoo.com)

**Background**

This study was aimed to identify the effect of covariates variables (education, knowledge, attitude of the mother, and the density of bedrooms), and immunization with diphtheria in children under 15 years in the city of Padang based on Geographic Information System.

**Materials and methods**

A case-control study was conducted to the population of mothers of children under 15 years in the city of Padang whereby diphtheria cases (probable cases and confirmed case) were existed. A total of 102 samples were taken by simple random sampling, matched by gender and region of residence. Controls were the closest neighbors to the cases. Data were collected through interviews by using questionnaires. Data were analyzed by using spatial analysis.

**Results**

Bivariate analysis showed that immunization status (OR=2.42, 95%CI:1.00-5.85), mother’s education level (OR=0.33, 95%CI:0.12-0.9) and the density of bedrooms (OR=3.2, 95% CI: 1.17-8.7) were linked to diphtheria. Knowledge and attitude of the mother were not related to diphtheria. Final multivariate modeling indicated that mother’s education, mother’s attitude, and density of bedrooms were confounders to the immunization status and diphtheria.

**Conclusions**

Mother’s education, mother’s attitudes and density of bedrooms influenced the relationship between immunization status and diphtheria. Thus, it is expected that the local public health center to improve health promotion program regarding immunization and diphtheria, especially in the Kuranji Timur, Padang and North Padang sub-districts.

## O14 The epidemiological study of pediculosis capitis among students in public primary school 08 of North Moramo Sub-district, Indonesia in 2016

### Yusuf Sabilu, Nurhijrianti Akib, Andi Faizal Fachlevy, Syawal Kamiluddin Saptaputra, Ruslan Majid, La Dupai

#### Public Health Faculty of Halu Oleo University, Kendari, South East Sulawesi, Indonesia

##### **Correspondence:** Yusuf Sabilu (yusufsabilu@yahoo.com)

**Background**

Pediculosis capitis (PC) is a skin disease caused by infestation of parasite *Pediculus humanus capitis* that grows and develops in the lining of a human scalp. The purpose of this study was to determine the epidemiological illustration of PC among students in public elementary school 08 of North Moramo, Indonesia in 2016.

**Materials and methods**

The method in this study was descriptive cross sectional with epidemiological study approach. The populations in this study were all students in public elementary school 08 of North Moramo in 2016. The samples in this study were 49 respondents taken by using purposive sampling method.

**Results**

The results showed that most of students with PC patients were female (100%), 7-9 years (89.5%), middle-haired and long-haired (100%), and rare shampoo frequency (84%). It was found that students with PC were those who frequently made contact with a pillow or bedding along (91.7%) and used a comb or hair accessories along with (91.9%). This study found that 87.5% of students with PC had bedroom which did not meet health requirements.

**Conclusions**

It can be concluded that most of students with PC were female and had bad hygiene.

## O15 Application of climate information for developing early warning model for controlling Dengue Hemorrhagic Fever incidence in endemic area

### Ririh Yudhastuti, Setya Haksama, M. Farid D Lusno, Aditya Sukma Pawitra

#### Faculty of Public Health, Universitas Airlangga, Surabaya, 60115, Indonesia

##### **Correspondence:** Ririh Yudhastuti (ririh.unair@gmail.com)

**Background**

Dengue Hemorrhagic Fever (DHF) incidence has been recorded in Surabaya, Indonesia since 1968. Many efforts have been taken to control the diseases up to 2017. This happens as a consequence of climate, reactive solution and imprecise consideration to anticipate the diseases. The research was aimed to develop Dengue Early Warning Model which can be utilized to provide an input to anticipate and control the incidence rate (IR).

**Materials and methods**

This research used secondary data of DHF incidence taken from Surabaya Health Agency in 2005-2015 and from BMKG Perak Surabaya station in 2005-2015. Map was made to determine the endemic areas and statistical test was employed.

**Results**

The results showed that the rate and period of DHF incidence can be predicted by using climate information i.e. three weekly moving average of rainfall and two weekly moving average of maximum, minimum and mean of air temperature. The accuracy of IR prediction model can be increased by utilizing the non-climatic information.

**Conclusions**

DHF Early Warning Model are consist of a number of DHF incidence and the average of weekly temperature with one week lag prior to prediction period. The development of DHF Early Warning System will be useful for predicting the incidence rate, assisting the anticipation and mitigating efforts to the decease incidence by determining the optimum time to reduce mosquito nests and deciding the time of fogging.

## O16 Gastritis incidence among senior high school students in Pekanbaru, Indonesia

### Dwi Sapta Aryantiningsih, Winda Parlin

#### Prodi IKM STIKes Payung Negeri, Pekanbaru, 28291, Indonesia

##### **Correspondence:** Dwi Sapta Aryantiningsih (dwisapta.ryan@payungnegeri.ac.id)

**Background**

Gastritis is an inflammatory process in the mucosa and sub mucosa of the stomach. Gastritis is the most common disorder in adolescents, because the diagnosis is often based only on clinical symptoms rather than histopathology examination. According to data from Pekanbaru Health Department, gastritis is included into the 10 most common diseases (1824 adolescent cases in 2014) and continues to increase from year to year. This study aimed to determine factors associated with gastritis symptoms omong senior high school students.

**Materials and methods**

This research was quantitative analytic with cross sectional design. A total of 211 samples were interviewed. Data analysis was done by bivariate and multivariate.

**Results**

This study found that gastritis symptoms were related to diet with OR 21.01 (95% CI: 9.15-48.27), consuming alcohol or soda with OR 3.76 (95% CI: 1.49-9.46) and smoking with OR 0.248 (95% CI: 0.09-0.63). The confounding variables were consuming alcohol or soda and knowledge.

**Conclusions**

It can be inferred that poor diet has a dominant effect on gastritis symptoms that experienced by students. It is suggested for students to change their behavior on diet and to pay more attention on their diet, nutrition of food they frequently eat and their lifestyle in order to avoid the symptoms of gastritis.

## O17 The difference of risk perception after getting education by using genogram simulation and paper-based Diabetes Risk calculator on patient with Diabetes family history

### Ta Larasati^1,2^, Nur I Lipoeto^2^, Hafni Bachtiar^2^, Mudjiran^3^

#### ^1^Community and Family Medicine Department, Medical Faculty, University of Lampung, Bandar Lampung, Lampung, 35141, Indonesia; ^2^Faculty of Medicine, Andalas University, Padang, 25147, Indonesia; ^3^Padang State University, Padang, 25171, Indonesia

##### **Correspondence:** Ta Larasati (t_a_larasati@yahoo.co.id)

**Background**

Recent evidence of family history of diabetes is an independent risk factor for type 2 diabetes mellitus. Therefore, current prevention of this type is necessary for the patient to do healthy behavior through intention and awareness of the risks. This study was aimed to determine the difference of risk perception among the patients after educated by using Genogram simulation and paper-based diabetes risk calculator.

**Materials and methods**

The design of the study was a non-randomized controlled trial. Subjects were identified with restriction criteria including patient with a family history of type 2 Diabetes Mellitus, 19-50 years old, and having no evidence of Diabetes. The subjects were 35 patients who were divided into control group and 2 intervention groups. One intervention group was educated by using Genogram simulation and another group was educated by using paper-based diabetes risk calculator. Risk perception was identified by using validated questionnaire. Data were analyzed by using Kruskall Wallis test and were continued by using Mann Whitney U test with 95% level of confidence.

**Results**

There were significant differences of risk perception after the intervention of the control group, using genogram simulation (p=0,001) and using the paper-based diabetes risk calculator (p=0,039), but there was no difference in risk perception between the intervention groups.

**Conclusions**

There were significant differences in risk perception between the control group and intervention group educated by using simulation genogram and those educated by using diabetes risk calculator.

## O18 Evaluation of subjective symptoms and the concentration of benzene, toluene and xylene exposure in shoe manufacturing industry

### Taufik Ashar, Evi Naria, Devi Nuraini Santi

#### Departement of Environmental Health, University of Sumatera Utara, Medan, 20155, Indonesia

##### **Correspondence:** Taufik Ashar (doctta@gmail.com)

**Background**

Shoe industry is one of the oldest industry. Hazardous chemical substances such as adhesives containing benzene, toluene and xylene (BTX) are used in manufacturing process. Due to lack of studies on exposure to BTX among shoemakers in Medan city, this study was aimed to assess subjective symptoms and the concentrations of BTX compounds in shoe workshops in Medan city, Indonesia.

**Materials and methods**

A total of 47 shoemakers in 7 workshops in Medan were selected for this study and monitored for BTX concentrations. Ambient air samples were taken based on NIOSH Manual of Analytical Method number 1501. Target compounds were analyzed by gas chromatography which equipped with flame ionization detector.

**Results**

The most frequently respiratory or neurologic symptoms reported by workers were cough (40.4%), followed by dizziness (55.3%). For eyes, 55.3% of respondents complained eye irritation. The range concentrations of benzene, toluene and xylene obtained were 0.09 ppm to 2.83 ppm, 1.20 ppm to 178.98 ppm, and 0.11 ppm to 50 ppm, respectively. From ambient measurements, the proportion of workers exposed to exceeding the Indonesian Occupational Exposure Limit was toluene (36.2%) and benzene (25.5%). There were 2 surveyed workshops (28.57%) with improper ventilation system.

**Conclusions**

The results showed that benzene and toluene levels were over the occupational exposure limits, and the subjective symptoms had affected more than half workers. Therefore, it is suggested that the workers should use a cartridge mask to protect their health. Moreover, local exhaust ventilation should be installed to provide sufficient ventilation system.

## O19 The association of cadmium and malondialdehyde in urine and Parkinsonism symptoms in community that exposed to cadmium from drinking water

### Taufik Ashar, Surya Dharma

#### Department of Environmental Health, University of North Sumatera, Medan, 20155 Indonesia

##### **Correspondence:** Taufik Ashar (doctta@gmail.com)

**Background**

Cadmium (Cd) is metal toxic that affect the function of nervous system. This study was aimed to analyze the association of cadmium intake from drinking water with Parkinsonism symptoms and to examine the correlation between urinary cadmium, urinary malondialdehyde (MDA) as one of oxidative stress marker and Parkinsonism symptoms.

**Materials and methods**

This study was analytic observation with cross sectional design, performed in community around Namo Bintang dumpsite, Medan City, Indonesia. Urine samples were examined to identify Cd and MDA. Validated Parkinsonism symptoms questionnaire was used to obtain nervous system disorder.

**Results**

The findings showed that Parkinsonism symptoms found among exposed group were screaming nightmares (62.5%), weakness of limb (55.5%) and stiffness rigidity in legs (51.8%). Among unexposed group, troublesome concentration and memory (72.4%), stiffness rigidity in legs (65.5%), and screaming nightmares (58.6%) were the most frequent symptoms. This study found that exposure to Cd from drinking water had no significant association with Parkinsonism symptoms. However, negative correlation had been reported between MDA and Parkinsonism symptoms (r=-0.331, p=0.013).

**Conclusions**

This study suggested that the exposure of Cd could cause oxidative stress, but did not affect the function of nervous system.

## O20 The distribution of potential pollutants in the area of River Code, Yogyakarta City, Indonesia

### Ahmad Faizal Rangkuti^1^, Musfirah^2^

#### ^1^Faculty of Public Health, Universitas Ahmad Dahlan, Yogyakarta, D.I. Yogyakarta, 55165, Indonesia; ^2^Faculty of Public Health, Universitas Ahmad Dahlan, Yogyakarta, D.I. Yogyakarta, 55165, Indonesia

##### **Correspondence:** Ahmad Faizal Rangkuti (faizal.rangkuti@ikm.uad.ac.id)

**Background**

River Code crosses Yogyakarta city. Rapid city development leads to the increasing number of pollutant sources that potentially pollute the environment of the River Code. The impact of pollution to River Code will contaminate wells which are sources of clean and drinking water for the surrounding residents.

**Materials and methods**

The research has been done by using observational method. The research was conducted along River Code.

**Results**

The research findings showed that along the River Code, there were potential sources of pollution including hospitals, hotels, farms and houses that produced organic waste, non-organic, and medical waste. Residents still used wells as source of drinking water or domestic use. A total of 11 out of 18 observed wells located along the river were in less than 10 meters distance from the river.

**Conclusions**

In River Code area, there are potential sources of pollutants. The existence of pollution can affect the quality of water in the wells which are used by the community as drinking water source.

## O21 Determinants of sustainable waste management intention behavior on junior high school students in Padang, Indonesia

### Aria Gusti (aria.mkes@gmail.com)

#### Department of Epidemiology & Biostatitstics, Faculty of Public Health, Andalas University, Padang, 25147, Indonesia

**Background**

The low awareness of the population to implement sustainable waste management behavior has an impact on increasing the amount of waste in the environment. This study aimed to analyze the influence of attitudes, subjective norms, and Perceived Behavior Control (PBC) on sustainable waste management intention-behavior and the influence of knowledge on attitude toward sustainable waste management behavior.

**Materials and methods**

This research was a quantitative research with the cross-sectional design. The population was the students of Junior High School in Padang City. The sample was taken by using proportional random sampling technique. Data were analyzed by using structural equation model (SEM) with AMOS program 2.1.

**Results**

The results of this study indicated that knowledge affected students’ attitudes toward sustainable waste management (CR = 2.067; p = 0.039). Attitudes had an effect on intention behavior (CR = 3.479; p = 0.001). The subjective norm had an effect on intention behavior (CR = 5.516; p = 0.001). PBC had an effect on intention behavior (CR = 2.532; p = 0.011).

**Conclusions**

Attitude, subjective norms, and PBC are determinants of sustainable waste management intention-behavior on junior high school students in Padang. It is suggested to the Education Office of Padang City to include teaching materials on sustainable waste management behavior into the environmental education curriculum of Junior High School.

## O22 An analysis of occupational safety and health risk at production division with Hirarc method in Batanghari Barisan rubber company, Padang City, Indonesia

### Nopriadi, Mitbasman Mikra, Nizwardi Azkha

#### Department of Environmental and Occupational Health, Faculty of Public Health, Andalas University, Padang, 25147, Indonesia

##### **Correspondence:** Nopriadi (nopriadi_dhs@yahoo.com)

**Background**

The interaction between human, tools, and environment in any job have a risk and potentially lead to occupational accident. Batanghari Barisan Company was one of rubber companies located in Padang City. In one of these industries, it had been documented about 132 cases of occupational accidents. This research aimed to analyze risk management (HIRARC) in production division of Batanghari Barisan Company as an effort to discover the risks and hazards of occupational health.

**Materials and methods**

The study design was qualitative and using HIRARC method to analyze health and occupational risk. The data were collected through in-depth interview, field observation, and document review. Sampling technique used was purposive sampling. Data analysis was started by scoring the risk.

**Results**

The result showed that there were hazard resources such as “*gancu*” (hook), trucks, forklift, floor, production machine, lift, trolley, carts, and caustic soda. Risk scoring in production division was divided as 14 high risks, 71 moderate risks, and 1 low risk. The risk controlled that had been practiced were the using of PPE (Personal Protection Equipment), monthly machine maintenance and permit work of forklift (License Work).

**Conclusions**

There were 7 hazard resources averagely in each division. Meanwhile, risk assessment in production division, in general, was low, and risk controlling had not been appropriate. The company is suggested to increase their attention in implementing occupational safety and applying Indonesia Government Decree No. 50/2012 about Occupational Safety Management System in the company.

## O23 The quality of water of Mahakam River at Loa Duri village, Kutai Kartanegara District, Indonesia

### Hansen, Lisa Wahidatul Oktaviani, Erni Wingki Susanti

#### Stikes Muhammadiyah Samarinda, Samarinda, 75123, Indonesia

##### **Correspondence:** Hansen (sri.sunarti@gmail.com)

**Background**

Mahakam River is surrounded by industry, coalmining, palm oil processing, water transportation, fisheries, agriculture, powerplants, farm, shipyards, and domestic activities. Besides, the river water is intended as a fresh water and clean water source by the community. This study was aimed to identify water quality of Mahakam River at Loa Duri Village.

**Materials and methods**

Ths study used cross sectional research design. Water samples were analyzed physically in laboratory.

**Results**

This study found that TDS concentration ranged from 29 mg/l to 69.00 mg/l. TSS concentration ranged from 07.00 mg/l to 20.00 mg/l. Water Ph ranged from 1.10 mg/l to 8.53 mg/l. BOD concentration ranged from 2.14 mg/l to 3 mg/l. The concentration of COD ranged from 10.70 mg/l to 25 mg/l. Concentration of iron ranged from 0.45 mg/l to 0.55 mg/l.

**Conclusions**

TDS and TSS concentration in Mahakam River were below the standard limit, whereas raw Fe (iron), BOD, COD exceed the standard of quality. Health problems experienced by the community in Loa Duri including the disruption of skin due to using river water for daily needs. The local government needs to undertake quality monitoring of Mahakam river water periodically and implement the clean river program and conduct socialization on environmental health and public health.

## O24 Risk assessment of total suspended particulate among unloading workers in Teluk Bayur Harbor in 2016

### Azkha Nizwardi, Rini Merli Sulistia

#### Andalas University, Padang, 25121, Indonesia

##### **Correspondence:** Azkha Nizwardi (nizwardi.azkha@yahoo.co.id)

**Background**

The industrial area has a big contribution to air pollution. Port of Teluk Bayur area is marked as the industrial area in Padang City. According to Environmental Impact Management Agency of Padang City, Teluk Bayur Harbor is designated as regional port of West Sumatera with no air quality monitoring, especially the concentration of TSP in the region.

**Materials and methods**

The study was an environmental health risk assessment. It was carried out in the port of Teluk Bayur during March to May 2016 with 97 respondents of unloading workers. Accidental sampling was employed.

**Results**

This study found TSP concentration at 0.48 mg/m3, the average age of workers was 55 years old, with the median of time exposure among workers was 12 hours per day, and the median of exposure frequency among workers was 182 days/year. The workers have been worked for 30 years in average, and the average weight of workers was 66 kg. Dose-response of TSP was 2.42. The intake of non-carcinogenic was 0.03613 mg/kg/day. RQ was found at less than 1.

**Conclusions**

It is expected that the government and port industry can do further studies and routine monitoring of the concentration of TSP.

Acknowledgements

Thanks to a participant of Unloading Workers in Teluk Bayur Port and other who assisted in this research.

## O25 The effect of ajwa date on lactic acid increase for physical fatigue recovery: an experimental study in rats

### Fatmawaty Mallapiang^1^, Azriful^1^, Muhammad Anwar Hafid^2^

#### ^1^Public Health Department in Medical and Health Science Faculty, Universitas Islam Negeri Alauddin Makassar (UIN), Makassar, 92113, Indonesia; ^2^Nursing Department in Medical and Health Science Faculty, Universitas Islam Negeri Alauddin Makassar (UIN), Makassar, 92113, Indonesia

##### **Correspondence:** Fatmawaty Mallapiang (fatmawaty.mallapiang@uin-alauddin.ac.id)

**Background**

Fatigue restoration by women workers using natural matters becomes a very important issue in work productivity improvement. Over-physical loading is one of the causes of physical fatigue. Ajwa date is rich of nutrition, thus it is necessary to study its potential use on lactic acid recovery. This study aimed to investigate the impact of different doses of Ajwa Date on lactic acid recovery with the induction of physical activity.

**Materials and methods**

An experimental study was conducted with pre- and post-test. This study involved 20 female rats divided into study groups and control group. The study groups were classified to three different groups related to the dose of Ajwa Date, it was low-dose (A), medium-dose (B), high-dose (C)) and the control group (D). All groups swam for 30 minutes with 5% of the body loads and measured for the lactic acid levels at pre-, after 5 minutes and 30 minutes swim.

**Results**

This study found that there was no difference on lactic acid levels between groups in 5 minutes after a physical activity and a significant difference was found in 30 minutes after a physical activity. The significant difference was found at the group of moderate and high dose.

**Conclusions**

The effect was found at a high dose of Ajwa Date with induction of physical activity and loading. A difference was found between the middle-dose and high-dose groups on lactic acid recovery with induction of physical activity and loading.

## O26 Knowledge of cleaning workers on Personal Protective Equipment (PPE) and its implementation at Faculty of Public Health, Universitas Indonesia

### Fatmi Yumantini Oktikasari^1^, Ladesi Natalia Nababan^1^, Herliyanti Yadi^2^

#### ^1^Department of Biostatistics, Faculty of Public Health, Universitas Indonesia, Depok, 16424, Indonesia; ^2^Department of Health Policy and Administation, Faculty of Public Health, Universitas Indonesia, 16424, Depok, Indonesia

##### **Correspondence:** Fatmi Yumantini Oktikasari (miyo.sari@gmail.com)

**Background**

Occupational disease tends to increase every year and it is associated with the usage of personal protective equipment (PPE). Preliminary study in faculty of public health Universitas Indonesia showed that most of cleaning workers did not use PPE during working. This study is aimed to explore the knowledge of cleaning workers regarding PPE and their behavior on PPE use while they are on duty in working area.

**Materials and methods**

This study was a qualitative research with rapid assessment methods. Data collection was conducted through in-depth interviews to cleaning workers, supervisors and management. Field observation and document observation were also done in collecting data. Triangulation of informant and method were implemented for validation purpose.

**Results**

Most of cleaning workers understand their job description with the details and functions of personal protective equipment. They do understand regarding the importance of personal protective equipment use while duty. In the working area however, majority of them use personal protective equipment rarely with some reasons.

**Conclusions**

Cleaning workers have good knowledge about personal protective equipment. In contrast, the application of PPE use in working area is still lacking.

## O27 Availability of personal protective equipment and standard operating procedure with usage behavior in cleaning workers at Faculty of Public Health, Universitas Indonesia

### Indah Widiyaningrum, Ryza Jazid BN, Dian Hardiani

#### Department of Biostatistics, Faculty of Public Health, Universitas Indonesia, Depok, 16424, Indonesia

##### **Correspondence:** Indah Widiyaningrum (indahwidiya@gmail.com)

**Background**

Potential health problems for workers are work accident and occupational diseases. The number of work accident cases increased in Indonesia in the last 5 years. A total 96,314 cases were reported in 2009 and increased to 103,285 in 2013. The number of occupational diseases in 2011 was 57,929 cases and decreased to 40,694 in 2014. Occupational disease which often occurred in cleaning workers is contact dermatitis due to the exposure of chemical substance from cleaning material. The purpose of this study was to explore the availability of Personal Protective Equipment (PPE) and Standard Operating Procedure (SOP) and cleaning worker’s behavior in PPE usage on working area.

**Materials and methods**

This study was a qualitative research with rapid assessment method. Triangulation of method and informant was used to validate the study result. In-depth interviews and observation were implemented for data collection. The informants of this study were cleaning workers and the supervisor.

**Results**

It was observed that most of cleaning workers did not use PPE. Most of informants said that the availability of PPE was insufficient. Some types of PPE were available in the storage in large quantities, but some other types were not available. SOP for PPE use was available, but most of cleaning workers did not read it.

**Conclusions**

The availability of PPE was incomplete, but some types of PPE were in stock. SOP for the PPE use was available but have not been fully implemented. Optimum supervision was required for the use of PPE among cleaning workers.

## O28 Psychological hazards among air traffic controller (ATC) in Air Nav Indonesia, Makassar Branch

### Lalu Muhammad Saleh^1^, Syamsiar S. Russeng^1^, Hasanuddin Ishak^2^

#### ^1^Department of Occupational Health and Safety, Public Health Faculty, University of Hasanuddin, Makassar, 90245, Indonesia; ^2^Department of Environmental Health, Public Health Faculty, University of Hasanuddin, Makassar, 90245, Indonesia

##### **Correspondence:** Lalu Muhammad Saleh (lalums@unhas.ac.id)

**Background**

Psychology of a person especially for workers can be changed any time following the harmony of emotions, work activities, environment and other factors, which ultimately can affect the occurrence of disease both physically and mentally. ATC needs high concentration and in a monotonous atmosphere which predicted to affect the psychological aspects of ATC. However, the psychological hazard by ATC cause of influence work activities such as boring, stress, and burnout should be addressed immediately.

**Materials and methods**

A cross sectional research design was used in this study and questionnaires were used for data collection. The Population was ATC Officers in Air Nav Indonesia Makassar Branch with 72 ATC officers was involved as samples. AMOS software was used in this study to perform SEM analysis.

**Results**

Our findings showed that psychological hazard and its risk occurred among ATC officers in Air Nav Indonesia, Makassar Branch. Psychological hazards found in this study included stress, boring, and burnout. The final model of psychological hazard and its risk showed that psychological hazard influenced health status and quality of life of ATC officers, but not to productivity.

**Conclusions**

Finally, this study conclude that there was evidence that psychological hazard can occur in ATC officers and contributes to health and quality of life of air traffic controllers.

## O29 The impact of implementation of quality management system of ISO 9001 at public health centers in Tangerang Selatan

### Fajar Ariyanti, Desty Pratiwi Marlisman, Ida Farida, Fitria Aryani Susanti, Arif Sumantri

#### Department of Health Management, Faculty of Medicine and Health Science, UIN Syarif Hidayatullah Jakarta, Tangerang Selatan, 15412, Indonesia

##### **Correspondence:** Fajar Ariyanti (fajar.ariyanti@uijkt.ac.id)

**Background**

Public health center (*Puskesmas*) as primary health facility in Tangerang Selatan has responsibility to provide good services to the community based on the ISO standard. However, there are no information regarding the quality of public health center after received ISO 9001 certification. The purpose of this study was to investigate and evaluate the implementation of quality management system of ISO 9001 in public health center in Tangerang Selatan.

**Materials and methods**

The population of the quantitative study was all patients who visited three Puskesmas which certified by ISO in the period of March to August 2016. The inclusion criteria of patients were productive age at 15 to 64 years old.

**Results**

The results showed that health centers could achieve the quality target by handling 100% patient complaints and regarding the time for providing services in maternal and child clinic, laboratory, and pharmacy. The number of patient visit in the laboratory, pediatric, dental, maternal and child clinic, and general clinic had exceeded the quality of target for ISO certification.

**Conclusions**

The ISO certification could improve quality of public health centers and increasing the number of patient visit who may recommend the services to other people.

## O30 Ergonomic posture application and musculoskeletal disorders among students of Faculty of Dentistry, Andalas University

### Murniwati^1^, Detty Iriani^2^, Rafika Maulina^1^, Nurul Khairiyah^1^

#### ^1^Faculty of Dentistry, Andalas University, Padang, 25127, Indonesia; ^2^Faculty of Medicine, Andalas University, Padang, 25127, Indonesia

##### **Correspondence:** Murniwati (murniwatihabib@yahoo.com)

**Background**

Dentist does dental work on a small area and uses lots of equipment. The habit of working without applying ergonomic posture may have an impact on musculoskeletal disorders. This research aimed to find out the relationship between ergonomic posture application and musculoskeletal disorders.

**Materials and methods**

This research used cross-sectional design and 33 students at Faculty of Dentistry of Andalas University were selected by purposive sampling. A form of check lists for ergonomic posture criteria and Cornell’s questionnaire of musculoskeletal discomfort was used as data collection instrument. The data was analyzed by using chi-square test.

**Results**

This study results showed that four out of eight of the ergonomic posture criteria were applied by the respondents. It was found that 60.6% respondents applied 3-4 ergonomic criteria where as 39.4% applied 1-2 criteria. Respondents who experienced musculoskeletal disorders once a day was 21.2% while those who experienced 3-4 times a week and 1-2 times a week were 30.3% and 30.3%, respectively. Respondents who applied 1-2 ergonomic posture criteria were more likely to experience musculoskeletal disorders compared with those who applied 3-4 criteria.

**Conclusions**

Ergonomic posture application is related to musculoskeletal disorders among students of faculty of dentistry, Andalas University. Musculoskeletal disorders is more frequent experienced by the respondents who applied less ergonomic posture criteria.

## O31 Analysis of work posture using Rapid Entire Body Assessment (REBA) as the risk factor of work-related musculoskeletal disorders among bus drivers in Bandar Lampung City

### Fitria Saftarina^1^, Diana Mayasari^1^, Dian Octaviani^2^

#### ^1^Department of Community Medicine, Faculty of Medicine, Lampung University, Bandar Lampung, 35145, Indonesia; ^2^Faculty of Medicine, Lampung University, Bandar Lampung, 35145, Indonesia

##### **Correspondence:** Fitria Saftarina (fitria205@yahoo.co.id)

**Background**

Bus driver is a job forcing the driver to work in static posture for a long time. Improper posture can be a risk factor of musculoskeletal disorders (MSDs). This study was aimed to identify work-related MSDs and its association with work posture and other related factors among bus drivers in Bandar Lampung City.

**Materials and methods**

This was a cross sectional study involving 101 inter-provincial bus drivers obtained by consecutive sampling technique. Musculoskeletal disorders were assessed using Nordic Body Map to identify the distribution of MSDs on the body, while work posture was measured using Rapid Entire Body Assessment (REBA) to evaluate the level of MSD risk.

**Results**

The prevalence of MSDs was 73.3% and were mostly located in the lower back (36.48%), calves (31.08%) and shoulders (28.38%). Most subjects (66.2%) had moderate risk of work posture. There was significant association between MSDs with high risk work posture (AOR=7.68; 95% CI=7.68-353.09); but there was no significant association with work period, age, nutritional status, and exercise.

**Conclusions**

High risk work posture is a risk factor of MSDs which significantly increase the risk of the occurrence of MSDs among bus driver. Ergonomic work posture is urgently applied to reduce the incidence of MSDs among drivers.

## O32 The relationship between noise intensity and blood pressure among employees in CV Tata Rizki, Pekanbaru City

### Winda Parlin, Kursiah Warti Ningsih, Rahmi Pramulia Fitri

#### Department of public health, Sekolah Tinggi Ilmu Kesehatan (STIKES) Payung Negeri, Pekanbaru, 28156, Indonesia

##### **Correspondence:** Winda Parlin (ihsanyuldi@gmail.com)

**Background**

This study aimed to reveal the prevalence of blood pressure among employees and its relationship to noise intensity in CV. Tata Rizki, Pekanbaru City, Indonesia.

**Materials and methods**

The design of this study was cross sectional, with a sample size of 211employees in CV. Tata Rizki, Pekanbaru City, obtained through systematic random sampling method. The data was analyzed by using chi square and logistic regression test.

**Results**

This study showed that high systolic and diastolic blood pressure was related to the intensity of the noise. The results showed significant correlation between noise intensity and blood pressure.

**Conclusions**

In CV Tata Rizki, noise intensity was correlated with employee’s blood pressure.

## O33 The role of health education in order to increase the knowledge of adolescents on the impact of bullying to personal health

### Riksa Wibawa Resna^1^, Dieta Nurrika^2^, Jessy Multiyana^2^

#### ^1^Nursing Department, Banten School Health of Science, Tangerang Selatan, 15318, Indonesia; ^2^Public Health Department, Banten School Health of Science, Tangerang Selatan, 15318, Indonesia

##### **Correspondence:** Riksa Wibawa Resna (riksawibawa@stikesbanten.ac.id)

**Background**

The number of bullying incident from year to year shows alarming figures. By 2013, about 40% of children in Indonesia were bullied in school and 32% were physically abused. This study aimed to see the role of health education conducted among adolescents toward student’s knowledge about bullying in school.

**Materials and methods**

This study used a pre and post-test design with control group study, with a sample of 74 students of class XI from a total of 101 students coming from 4 different programs in private vocational schools in South Tangerang city. Sampling was done by simple random sampling method and then divided into two different groups. The first group was given a health education with a youth approach for 3 times a week, while the other group was left without health education.

**Results**

Study showed the mean score of knowledge in the intervention group at the pre-test was 69.17 increased significantly to 85.38 with 16.21 mean difference and 4.89 standard deviation. The mean score of knowledge of control group descreased slightly from 56.26 to 55 with 1.04 mean different and 0.27 standard deviation

**Conclusions**

Methods of health education with youthful approach proved to be effective for increasing adolescent knowledge about the impact of bullying on health.

## O34 PROKESIMA (*Promosi Kesehatan Ibu Mandiri*) as health promotion model for pregnant women in Sampang, Madura, Indonesia

### Shrimarti Rukmini Devy (s_r_devy@yahoo.co.id)

#### Department of Health Promotion and Behavior Knowledge, Faculty of Public Health, Universitas Airlangga, Surabaya, 60286, Indonesia

**Background**

Non-communicable diseases (NCDs) are responsible as the causel of death among pregnant women. In Indonesia, pre-eclampsia is still among the leading causes of maternal death. PROKESIMA as a model of health promotion was implemented in a collaborative activity that intended to pregnant women. The objective of the study was to know the change of knowledge about the pre-eclampsia due to the implementation of PROKESIMA among pregnant women in Sampang, Madura.

**Materials and methods**

This study was an action research. PROKESIMA was a collaborative activity between health education, religious education and productive economic activity (knitting) which was performed during February-June 2017. This intervention involved nine pregnant women as the participants, midwife and midwife coordinator as the implementer of health education in PROKESIMA at Desa Rapa Daya, Sampang, Madura-East Java, Indonesia. PROKESIMA consisted of six meeting and performed weekly.

**Results**

Over 71% of informants was less than 24 years old and all of them had primary education and became housewife. Four out of 7 informants had their multipara pregnancy. The result of pre-and post-test showed that informant’s knowledge were increased by 60%.

**Conclusions**

PROKESIMA is an effective health promotion model to increase the knowledge among pregnant women in Desa Rapa Daya, Sampang, Madura.

## O35 Cost trend analysis on health promotion program in Medan City

### Destanul Aulia^1^, Sri Fajar Ayu^2^, Riska Afani^1^

#### ^1^Departement of Health Policy and Administration, University of Sumatera Utara, Medan, 20155, Indonesia; ^2^Departement of Agribusiness, University of Sumatera Utara, Medan, 20155, Indonesia

##### **Correspondence:** Destanul Aulia (destanul.aulia@usu.ac.id)

**Background**

Health promotion budget should be provided in sufficient quantity, allocated equitably and utilized properly to be beneficial to public health. This study aimed to determine the availability of costs, the proportion of funds, and forecast the budget of health promotion programs in Medan City.

**Materials and methods**

This study used descriptive analysis through quantitative and qualitative methods to calculate the government budget for health promotion program by using data from District Health Account (DHA). Data were collected by using questionnaire, while the secondary data were obtained from Medan local government expenditure documents and health promotion budget year 2012- 2016.

**Results**

The analysis showed that the total health budget of Medan local government in 2012-2016 was below 15% of its total budget. This insufficient funds hampered the implementation of several health promotion programs planned by Medan Health Office. It was found that health promotion budget plan did not consider macro-economic aspects, which caused a shortage in health promotion funds. In addition, some health promotion programs tend to be monotonous from year to year and has not been able to address the needs of the people of Medan City due to lack of advocacy towards local parliament and private parties.

**Conclusions**

Medan City health budget during 2012-2016 was still below 15%. Health promotion budgeting process did not consider macro-economic aspects. Health promotion programs need to be improved to meet the needs of the community.

## O36 Case report: the promotion of AMOBA App (Application of Mother and Baby)

### Savitri Citra Budi, Yuni Rahmawati, Shyfany Krismarestuti

#### Department of Information and Health Services, Vocational College, Universitas Gadjah Mada, Yogyakarta, 55281, Indonesia

##### **Correspondence:** Savitri Citra Budi (savitri@ugm.ac.id)

**Background**

Maternal and infant mortality rates are the benchmarks of a nation's welfare. Indonesia is the country with the highest maternal mortality rate in Southeast Asia. Application Mother and Baby (AMOBA App) is android based Application presented as a program to form an interactive community which focused on the health of pregnant women to women with under two years children. AMOBA App can provide information and become reminders of pregnancy and childbirth. AMOBA App facilitates the community to do a consultation with health personnel. This study aimed to identify community response towards AMOBA App.

**Materials and methods**

This research type was a descriptive study. Study subjects were midwife, community health cadres, AMOBA App cadres, pregnant women and women children under two years old in the working area of Jetis health center, Yogyakarta. Data were collected through observation techniques, interviews, and documentation studies. Source triangulation was used for validation.

**Results**

AMOBA App had been promoted through counselling and training, implementation phase to the community, and door-to-door assistance for the community. Positive response was found from pregnant women and women with children under two years old.

**Conclusions**

Community responsed positively to AMOBA App. This program need support from policy makers to ensure the sustainability of its implementation.

## O37 Effectiveness of conseling on controlling fluid intake among hemodialysis patients in Adam Malik Hospital, Indonesia

### Nunung F. Sitepu^1^, Rena Betty Pasaribu^2^

#### ^1^Department of Medical Surgical, Faculty of Nursing, University of Sumatera Utara, Medan, 20155, Indonesia; ^2^Adam Malik Hospital, Medan, 20136, Indonesia

##### **Correspondence:** Nunung F. Sitepu (nunung.febriany@gmail.com)

**Background**

Weight gaining between two dialyses is an indicator of fluid intake during the period of hemodialysis which furthermore, it causes various problems including hypertension, peripheral edema, pulmonary edema, and the risk for dilatation and cardio-hypertrophy. The objective of the research was to find out the effectiveness of conseling on controlling fluid intake among hemodialysis patients in Adam Malik Hospital, Indonesia.

**Materials and methods**

This study used quasi-experiment method with one group pre-test and post-test design. The intervention was counseling with health education. The respondents’ weight was measured, health education on fluid intake was provided to the respondents, and then respondents’ weight was measured again. It was conducted in four weeks among 28 respondents, the patients of hemodialysis in Adam Malik Hospital, Indonesia. The samples were taken using purposive sampling and Wilcoxon Test was used for statistical test.

**Results**

The result of the analysis showed that there was a weight loss in patients with hemodialysis after counseling on fluids intake.

**Conclusions**

There was a weight loss in patients with hemodialysis after counseling about fluids intake. It is recommended that patients with hemodialysis participate in counseling so that the limitation of fluid intake for the next hemodialysis can be controlled by themselves independently.

## O38 Climate change and its impact on health: environmental health students’ awareness for protecting public health

### Putri Nilam Sari (nilam.nofri@gmail.com)

#### ^1^Department of Occupational and Environmental Health, Faculty of Public Health, Andalas University, Padang, 25129, Indonesia

**Background**

Environmental health students will be environmental health experts in the future to promote health and prevent health problems due to climate change. They must have a good awareness regarding climate change and its health impacts. This study was designed to investigate the awareness level among environmental health students on global warming.

**Materials and methods**

This research was conducted to 44 students of Environmental Health Department at Faculty of Public Health, Andalas University by using cross sectional approach. Statistical analysis used were chi square and U Mann Whitney test to examine student awareness about the impact of climate change to public health.

**Results**

The results showed that 38.6% respondents had good behavior in anticipating climate change; 56.8% understood about the diseases caused by climate change; 18.2% had a good knowledge on climate change; and 77.3% had good information about the impacts of climate change. It was also found that 59% of them got information from social media. 56.8% had a good perception on the effect of pro environmental behavior to minimize the climate impact; and 56.8% had positive attitude to anticipate the climate change. Gender affected significantly the students’ knowledge on climate change and its impact on health. Their perception of the availability of safe and sufficient of water resources affected significantly to student’s behavior in anticipating climate change and understanding the diseases caused by climate change. Their attitudes toward the use of eco-friendly fuel also significantly influenced their understanding on climate change health impact.

**Conclusions**

It is advisable to the Association of Higher Education Institutions on Public Health to be able to integrate the subject of climate change and its health impact in the curriculum of environmental health majors in university. The health effects of global warming can be disseminated through social media so that students are more aware of the importance of protecting people from the effects of climate change.

## O39 Factors affecting utilization of maternal and child health handbook among mothers and midwives in Tangerang Regency, Banten Province, Indonesia

### Baequni (baequniboerman@gmail.com)

#### Medical and Health Sciences, Islamic State University Syarif, Hidayatullah, Tangerang, 15412l, Indonesia

**Background**

In the era of Sustainable Development Goals (SDGs), Maternal and Child Health Handbook (MCHHB) is becoming a tool for promoting maternal and child health care. MCHHB guidance maternal and child health services as a continuum of care as an effort to reduce maternal and child mortality rates. MCHHB program has been introduced in more than 25 countries including Indonesia. This study aimed to find the factors related to the utilization of MCHHB by the midwives and analyze the use of MCHHB among mothers in Tangerang Regency of Banten Province.

**Materials and methods**

The quantitative and qualitative methods were used to identify the factors affecting utilization of MCHHB by midwives and mothers. The respondents were 207 midwives and 259 mothers at village level in Tangerang regency.

**Results**

The study found that only 36.2% of all examination results were written by midwives in MCHHB. The mothers, on average, read only 30% of MCHHB materials in the handbook. The mothers living in urban areas owned MCHHB 1.49 times compared with the mothers living in rural area. The age of mothers showed significant association with MCHHB ownership (OR=1.24, 95% CI: 1.03-1.10). The midwives’ attitude was associated with the behavior of keeping the MCHHB in ready stock (OR=5.03, 95% CI: 1.03 – 24.49). The midwives’ motivation associated with using MCHHB to educate or explain mothers about the handbook (OR=2.5, 95% CI: 1.06 - 5.86), and giving a new MCHHB to mothers who lost the handbook (OR=1.9, 95% CI: 1.05- 3.65).

**Conclusions**

The minimum midwives’ utilization of the handbook caused poor MCHHB utilization among mothers. The necessity of modifying midwives’ motivation and attitude factors is needed to increase utilizing MCHHB.

## O40 The development of gerotranscendence among the elderly in Surabaya City, Indonesia

### Rachmah Indawati^1^, Kuntoro^1^, Hari B. Notobroto^1^, Mochammad B. Qomaruddin^2^

#### ^1^Department of Biostatistics and Population Study, Public Health School of Airlangga University, Surabaya, 60286, Indonesia; ^2^Department of Health Promotion and Behavioral Sciences, Public Health School of Airlangga, Surabaya, 60286, Indonesia

##### **Correspondence:** Rachmah Indawati (rachindawati@yahoo.com)

**Background**

Gerotranscendence is a process of life experienced by elderly people. Individual changes in the direction of gerotranscendence can not be separated from values ​​that developed in society. Indonesia is a country with multicultural and multireligion whereby the elderly group spends time to participate in religious activities. Families are responsible for the well-being of the elderly. In individuals, the elderly has a developmental task for aging experience. The objective of the study was to describe gerotranscendence changes according to individual characteristics.

**Materials and methods**

This research was an observational research with cross-sectional approach. Simple random sampling technique was used in elderly aged 60 years who lived in Surabaya.

**Results**

The average age was 68.7 years old ranged from 60 to 92 years. Majority of elderly were female (64.6%). In association with age and sex showed, the cosmic transcendence graph fluctuates with age. Women were more ‘cosmic transcendent’ than men. Cosmic transcendent women declined at age of more than 70 years and increased after more than 80 years whilst cosmic transcendent men increased at age 70 years and decreased at age 79 years. The graph of ‘self-dimensional’ showed the process of individual consciousness in understanding the very slow life varied across gender. The graph of ‘social dimension & personal relationship’ showed also varied across gender.

**Conclusions**

The development of gerotranscendence was known to be related to individual characteristics.

## O41 The cause of low vitamin D level in women with single nucleotide polymorphisms: a cross sectional study

### Dina Keumala Sari^1^, Sri Lestari^2^, Sunna Vyatra Hutagalung^3^, Ratna Akbari Ganie^4^

#### ^1^Department of Nutrition, Faculty of Medicine, Universitas Sumatera Utara, Medan, 20155, Indonesia; ^2^Department of Public Health, Faculty of Medicine, Universitas Sumatera Utara, Medan, 20155, Indonesia; ^3^Department of Parasitology, Faculty of Medicine, Universitas Sumatera Utara, Medan, 20155, Indonesia; ^4^Department of Clinical Pathology, Faculty of Medicine, Universitas Sumatera Utara, Medan, 20155, Indonesia

##### **Correspondence:** Dina Keumala Sari (dinaridha@yahoo.com)

**Background**

Studies showed that 95% of women with single nucleotide polymorphisms of the vitamin D receptor gene had a serum level of 25 (OH) D lower than normal in North Sumatera Province, Indonesia. The work aimed to investigate the cause of low vitamin D level in women with single nucleotide polymorphisms.

**Materials and methods**

This was a cross-sectional study engaging 198 women participants with vitamin D deficiency-insufficiency and single nucleotide polymorphisms of the vitamin D receptor gene. The scoring was conducted to assess the knowledge (on what is vitamin D and how the vitamin affecting health), attitude (on increasing vitamin D through lifestyle), and action (on how to implicate the lifestyle to increase vitamin D level).

**Results**

The study respondents aged between 20 to 50 years old with single nucleotide polymorphisms of *Taq*I and *Bsm*I and vitamin D level at 18.76±4.69 ng/ml in average. All samples were found to have low level of sunrise exposure, having sunblock application, low vitamin D intake, and low physical activities. According to knowledge, 60.5% respondents had low knowledge, 52.5% had a positive attitude, and 76.2% with lack of action. There was a significant association between knowledge and education (*p=*0. 01), but there was no significant association between knowledge and action.

**Conclusions**

Besides single nucleotide polymorphisms of the vitamin D receptor gene and lifestyle, there was no association between knowledge and action that could be the cause of low vitamin D level.

## O42 Factors related to exclusive-breastfeeding in the sub-district of Pauh, Indonesia

### Ratno Widoyo, Fauziah Elytha, Nining Fajri

#### Epidemiology and Biostatistics Department, Faculty of Public Health, Andalas University, Padang, 25129, Indonesia

##### Ratno Widoyo (ratno.one@gmail.com)

**Background**

In 2015, the prevalence of stunting in Pauh sub-district was found at 14%. Previous study found that low exclusive-breastfeeding coverage was associated with stunting in this area. This study aimed to determine factors related to failure in exclusive breastfeeding in Pauh sub-district, Indonesia.

**Materials and methods**

This study was conducted in April to November 2016, used comparative cross-sectional method. Samples were taken by simple random sampling from birth registration. Around 109 mothers of 6-12 month infants, have been interviewed.

**Results**

This study found that 38.5% of infants received exclusive breastfeeding. This study revealed that 59.63% of mothers fed their infants with mineral water, whereas 50.62% of respondents provided formula milk and other foods to their infants. Failure in exclusive breastfeeding was found among 70% of infants who were delivered through caesarean procedure and 62.5% of those with low birth weight. Furthermore, this study found that 75% of infants with diarrhea did not receive exclusive breastfeeding.

**Conclusions**

Feeding mineral water to infants caused exclusive-breastfeeding fails. Mostly, mothers also feed their infants with formula milk and other foods. It is necessary for health workers and cadres to support and remind the mothers regarding the importance of exclusive breastfeeding.

## O43 Enhancing the effectiveness of health promotion through highly tailored campaigns: the role of language and culture

### Rima Semiarty^1^, Rebecca Fanany^2^, Nursyirwan Effendi^3^

#### ^1^Departement of Public Health, Faculty of Medicine, Andalas University, Padang, 25129, Indonesia; ^2^School of Humanities and Social Sciences, Deakin University, Melbourne, 3011, Australia; ^3^Faculty of Social Science Faculty, Andalas University, Padang, 25129, Indonesia

##### **Correspondence:** Rima Semiarty (rimamenkher@yahoo.com)

**Background**

Health promotion has great value in changing health behavior and affecting various health conditions. However, it has also been observed that even well-developed health promotion campaigns often do not achieve their goals. The effectiveness of such campaigns can be enhanced through the use of highly tailored material that is consonant with the language and culture of the target community. This paper discusses the characteristics of highly effective tailored health promotion campaigns and assesses their ability to affect health behavior of a target population.

**Materials and methods**

Semiotic analysis is applied to specific examples of past and present campaigns in West Sumatra, Indonesia, to elucidate their relevance and impact on the target population. The dominant local culture is assessed as a source for effective health promotion campaigns.

**Results**

The results indicate that highly effective health promotion campaigns tend to fit cultural and linguistic expectations and use relevant expressions and imagery from the target culture. Such campaigns generate greater recall and comprehension among the target population and also have high approval.

**Conclusions**

Language and culture represent two important sources of material in health promotion. Correspondingly, it may be difficult to transplant campaigns that were successful in one location to another because of these linguistic and cultural differences which may affect the way the campaign is perceived. For this reason, it is important to tailor health promotion campaigns to the target population in light of its specific characteristics as well as its health status.

## O44 Supporting factors in socialization to ex-leprosy individuals in Cina District, Bone Regency, Indonesia in 2017

### Nurul Hafsanjani, Masriadi, Rahmawati Azis

#### Department of Epidemiology and Biostatistics, Institute of Health Science Tamalatea, Makassar, 90224, Indonesia

##### **Correspondence:** Nurul Hafsanjani (nurulhafsanjani@gmail.com)

**Background**

Leprosy is a contagious disease caused by *Mycobacterium leprae* and able to trigger complex issues. The problem faced by the ex-leprosy individuals is stigma around the society. Based on this issue, the study aimed to search the supporting factors in socialization to the ex-leprosy individuals.

**Materials and methods**

This type of research utilized mix method in sequential exploratory. The research was conducting in Cina District, Bone Regency on June to August 2017. There were 200 respondents selected by using cluster sampling and the qualitative part utilized the snowball sampling for data collection through in-depth interview. The quantitative data analysis used path analysis and the quantitative functioned triangulation data for validity test.

**Results**

The result found that knowledge was significantly influenced stigma (ρ=0.041; Standardized Coefficients β=0.176). Stigma had a direct influence on socialization to ex-leprosy individuals (ρ<0.05; Standardized Coefficients β=0.289). Respondents who had good knowledge regarding ex-leprosy individuals did not feel disturbed, however respondents still worried their children when ex-leprosy individuals were around.

**Conclusions**

Supporting factors in socialization to ex-leprosy individuals were knowledge and stigma. Knowledge was significantly influenced stigma and was significantly influenced socialization to ex-leprosy individuals.

## O45 The relationship between breastfeeding pattern and early childhood caries in Bukittinggi City, Indonesia

### Susi^1^, Murniwati^1^, Nila Kasuma^1^, Minarni^2^

#### ^1^Faculty of Dentistry, Andalas University, Padang, 25129, Indonesia; ^2^Health Polytechnic, Kementrian Kesehatan Padang, Padang, 25129, Indonesia

##### **Correspondence:** Susi (susiabidin@gmail.com)

**Background**

Early Childhood Caries (ECC) is defined as caries in primary teeth among children under 71 months. ECC affect children’s growth and development. Studies found that feeding patterns are associated with ECC. This study aims to investigate the relationship between breastfeeding pattern and ECC.

**Materials and methods**

This is a cross sectional study located in Integrated Post Service (*Posyandu*) in Bukittinggi City. In this study, 66 pairs of parents and children aged 2-3 years were selected. Breastfeeding pattern was observed using questionnaire and deft index was used for ECC. Data were analyzed by Chi square test.

**Results**

The average of deft index was 2.33. Samples with exclusive breastfeeding have an average deft index of 1.61 and non-exclusive have an average deft index of 3.52 (p=0.045). This study also found that samples with exclusive breastfeeding and complementary foods have deft index of 2.59. Non-exclusive breastfeeding and complementary foods have a deft index of 1.96 (p=0.930).

**Conclusions**

Exclusive breastfeeding at the age of 0-6 months had a significant relationship with ECC. Children with exclusive breastfeeding have a lower ECC degree. Breastfeeding with complementary foods at 6 months to 2 years didn’t have a significant relationship.

## O46 Strengthening the community empowerment program through voluntary community facilitator in Tragah, Bangkalan District, Indonesia

### Qurnia Andayani (qurnia.andayani-13@fkm.unair.ac.id)

#### Health Science Program, Universitas Airlangga, Surabaya, 60115, Indonesia

**Background**

The Ministry of Village, Underdeveloped Regions, and Transmigration of Indonesia launched a program of voluntary community facilitator for remote, rural and underdeveloped villages. The purpose of voluntary community facilitator is strengthening community empowerment especially on better nutrition for child and maternal. This studywas aimed to explore the causes of mother and child malnutrition, analyze the situation of community empowerment program for mother and child health, explore the roles of voluntary community facilitator for mother and child health.

**Materials and methods**

This qualitative study was conducted on March to October 2014. Data were collected by observation, in-depth interview and focus group discussion (FGD). The informants consisted of districts nutritionist, head of primary health care, sub district nutritionist, coordinator of midwife, 3 village midwives, 3 heads of village, 5 community figures, 3 cadres, and 9 mothers. FGD was held in 3 villages that choosen purposively, it was attended by village leader, village secretary, midwives, PHC staff, cadre and community. Sampling method was purposive sampling. Data were validated using triangulation method.

**Results**

Villagers were aware that their area has many problems but few of them knew that health was still an important issue. Based on FGD, empowerment programs such as *posyandu* weakened than before which were consider as natural process reflected that the programs needed revitalization and promotion. Thus, voluntary community facilitator could be a catalisator for strengthening health program particularly child and maternal health based on local knowledge and practices.

**Conclusions**

It needs commitment from community and health institution to collaborate with other sectors to increase health status in underdeveloped villages especially Bangkalan district.

## O47 Application of theory reasoned action in decision making of clean and healthy life behavior to prevent environment-based disease

### H. Adnani^1^, A. A. Subiyanto^2^, D. Hanim^3^, E. S. Sulaeman^2^

#### ^1^School of Health Sciences Surya Global Yogyakarta, Yogyakarta, 55196, Indonesia; ^2^Departement of Public Health, Faculty of Medicine, Sebelas Maret University, Surakarta, 57126, Indonesia; ^3^Masters Program in Nutrition, Sebelas Maret University, Surakarta, 57126, Indonesia

##### **Correspondence:** H. Adnani (adnani.hariza@yahoo.com)

**Background**

Theory of reasoned action links beliefs, attitudes, intentions and behaviors. The objective of this study was to determine the relationship between attitude, subjective norms, intentions and behavior on clean and healthy life through the application of reasoned action theory.

**Materials and methods**

This was a cross sectional study, located in Imogiri traditional market in Bantul, Yogyakarta Province, Indonesia. Study population was the market visitors. Accidental sampling was used to take the samples of 165 market communities who had visited the market more than twice. Data was collected by using questionnaire and observation sheet and structural equation modeling was conducted for data analysis.

**Results**

This study found that attitudes affected the intention and behavioral beliefs significantly. Subjective norms affected outcome evaluation (C, R = 29,657; p < 0,05). The intention significantly affected the intention of disposing of waste, intention of utilizing latrines, intention of not smoking in the market, intention to wash hands with soap. Clean and healthy life behavior had an effect on non-smoking behavior in the market and hand washing behavior with soap. The Goodness of Fit test showed that the model fit 5 requirements (NFI = 0.866, RFI = 0.815, IFI = 0.887, NNFI or TLI = 0843, CFI = 0.886).

**Conclusions**

Attitudes, subjective norms had a greater effect on intention than on clean and healthy living behaviors in preventing environment-based diseases.

## O48 Educational perspectives on health and healthy school: a qualitative study

### Muji Sulistyowati, Muthmainnah

#### Department of Health Promotion and Behavior Science, Faculty of Public Health, Universitas Airlangga, Surabaya, 60115, Indonesia

##### **Correspondence:** Muji Sulistyowati (muji-s@fkm.unair.ac.id)

**Background**

Health is directly linked to educational achievement. Health promoting school is characterized as a school constantly strengthening its capacity as a healthy setting for living, learning and working. Perspective, include educational knowledge, perception, and motivation can improve the implementation of school health program. This study aimed to to explore educational perspective (understanding, perception, and motivation) on health and school health program.

**Materials and methods**

This was a qualitative research with case study approach. Four FGDs have been conducted with the Principals and Health School Officers from 10 elementary schools in Surabaya District, East Java, Indonesia. Ten in-depth interviews also done to one teacher from each school. Content analysis was used to analyze the findings.

**Results**

Most of informants had good knowledge about health, even though not completely as mentioned in WHO health definition. The Principals and Health School Officers had fairly good knowledge and perception, but rarely were mentioned regarding mental and spiritual health. Moreover, teachers had limited knowledge on health. Health from teacher’s perspective was just about physic, clean environments, and healthy snacks. Almost all informants agreed that healthy school was about clean environments. Few of them mentioned about external party involvement in gaining healthy school.

**Conclusions**

All informants had fairly good understanding about health and school health, but still stressed on physical environment. They had high motivation to keep and to promote health behavior at school. It implies that training and accompaniment to promote healthy school program among educational is needed.

## O49 Risk factors of smoking among smokers in tobacco-producing areas

### Kusyogo Cahyo, Aditya Kusumawati

#### Health Promotion & Behavioral Science Department, Public Health Faculty, Diponegoro University, Semarang, 50275, Indonesia

##### **Correspondence:** Kusyogo Cahyo (kusyogocahyo@undip.ac.id)

**Background**

Smoking caused 235,000 deaths among smokers in Indonesia each year and 25,000 non-smokers deaths. Currently, as many as 36.1% of adults in Indonesia smoke. Not only in smokers, diseases caused by exposure to tobacco can also be suffered by farmers because the tobacco farmers are exposed to harmful substances from cigarettes and tobacco itself. This study examined the associated factors of smoking behavior among smokers who lived in tobacco-producing areas.

**Materials and methods**

This study was an analytic research with cross sectional study design. Samples were 100 smokers in tobacco commodities areas in Weleri Kendal, Indonesia.

**Results**

The respondents at most were late adults (29%) and no late adolescents. Almost all respondents were male (96%). Most respondents were laborers or peasants (80%). The bivariate analysis showed the association between lung capacity and smoking behavior. Respondents with light restriction lung capacity were 40.0%, greater proportion than those with normal lung capacity (39.0%), moderate restriction lung capacity (12.0%), and poor restriction pulmonary capacity (9.0%). The respondent's knowledge about the impact of smoking is good (57%), greater than the proportion of respondents with less good knowledge about smoking effect (43%). Of the 100 respondents, 84% were active smokers. However, there was no association between social and cultural factors, access to health care, knowledge, attitude and media access to information toward smoking behavior.

**Conclusions**

The lung capacity was related to smoking behavior of smokers in the tobacco commodity area in Weleri Kendal. Further research is needed to identify deeper economic factors.

## O50 The effectiveness of personal hygiene promotion among elderly in Nursing Home Wisma Mulia, West Jakarta, Indonesia

### Intan Silviana Mustikawati (intansilviana@esaunggul.ac.id)

#### Department of Public Health, University of Esa Unggul, Jakarta, 11510, Indonesia

**Background**

The ageing population is particularly at risk because their immune systems slow down and susceptible to disease. Health promotion has been proposed as an effective way to deliver a program based on structured knowledge to enhance emotional support among people, including to improve personal hygiene in elderly. The purpose of this study was to measure the effectiveness of personal hygiene promotion in improving personal hygiene knowledge among elderly in Nursing Home Wisma Mulia, Jakarta, Indonesia.

**Materials and methods**

A quasi-experimental method was used with a pre-test and post-test design among 75 elderlies in Nursing Home Wisma Mulia, Jakarta who were randomly selected. Interview was used to measure personal hygiene knowledge before and after health promotion. Paired t-test was used to analyze the data.

**Results**

Majority of respondents were female (82.7%), aged 60-74 years old (57.9%), low education (65.3%), had working before (65.3%), no health insurance coverage (81.3%), less health information access (52%), had access to health facility (62.7%), and had adequate facility (84.2%). The results showed a statistically significant increase of personal hygiene knowledge (t = -6,542, p < 0.05) between pre and post-test.

**Conclusions**

There was an improvement of personal hygiene knowledge after a personal hygiene promotion. There is a need of communication, information, and education activities of personal hygiene benefits to be continuously conducted in order to increase knowledge; support from family, nursing home, and health workers. Further study exploring another method to improve knowledge of personal hygiene among elderly is recommended.

## O51 Knowledge and attitudes about reproductive health (Preliminary study of brides and grooms in Brebes District, Central Java, Indonesia)

### Nugraheni S.A^1^, Martini^1^, Istiarti T^1^, Johan I^2^, Sulistyowati E^1^

#### ^1^Faculty of Public Health, Diponegoro University, Semarang, 50275, Indonesia; ^2^Faculty of Health Sciences, Universitas Pesantren Tinggi Darul Ulum, Jombang, 61481, Indonesia

##### **Correspondence:** Nugraheni S.A (s.a.nugraheni.undip@gmail.com)

**Background**

Knowledge of brides about reproductive health and pregnancy preparedness in Indonesia still lack. There are so many brides and grooms who do not know how to maintain good reproductive health and prepare their pregnancy. Efforts to give lessons on reproductive health, sex education and pregnancy preparedness to brides and grooms today are still limited. Education on reproductive health and pregnancy preparedness is one of strategic ways to increase brides’ knowledge and attitudes on reproductive health. This study was conducted to evaluate an effect of reproductive health education and provision of media on brides and grooms in Brebes District.

**Materials and methods**

This study utilized the quasi experimental pre and post test one group design. Samples in this study was 54 brides and grooms (23 males and 31 females) in Brebes.

**Results**

Based on the results of a different test with Wilcoxon Match paired test, there was significant differences between respondents’ knowledge and attitudes before and after reproductive health education and provision of media (p = 0.008). That means there was influence of interventions in the form of education and provision of media to increase knowledge and attitudes of respondents about reproductive health.

**Conclusions**

There was a significant difference between knowledge and attitude of a brides and grooms in Brebes before and after obtained counseling. The study found that this intervention could significantly improve knowledge and attitude of the respondent. Shared responsibility for reproductive health matters by males and females would also be made.

## O52 Adolescents, partners and parents’ response to an unwanted pregnancy in Central Java, Indonesia: a qualitative study

### Aprianti^1^, Zahroh Shaluhiyah^2^, Antono Suryoputro^2^

#### ^1^Faculty of Public Health, Andalas University, Padang ,25128, Indonesia; ^2^Faculty of Public Health, Diponegoro University, Semarang, 50275, Indonesia

##### **Correspondence:** Aprianti (aprianty17@gmail.com)

**Background**

Unwanted pregnancy cases have been risen and more frequently found in early adolescents. It leads to a higher risk of maternal mortality in Indonesia. In Pati District, the unwanted pregnancies of adolescence under 17 years old has reached 43.8% in 2016. This study aimed to describe adolescents, partners and parents’ responses to unwanted pregnancies and their effects on adolescent health status.

**Materials and methods**

This was a qualitative research, interviewing 5 adolescents, their partners and parents. Data was collected by in-depth interviews to adolescents, partners and parents and focus group discussion was conducted to the surrounding community for triangulation. Five village midwives were interviewed as complement.

**Results**

Main informants consisted of 13 to 16 years old teenagers. Their responses to the unwanted pregnancy were anxiousness and feared, sad, depressed and trying to terminate the pregnancy. The early partners’ responses were shocked, feared, or depressed, before making a decision for marrying. Only one partner said that not willing to marry. The most earlier responses from the parents were disappointed, and some of them asking for termination of pregnancy. The next responses were the parents agreed that their children should be married. Psychological impacts for adolescents were fear of delivering disabled child, guilty, and feeling unprepared of being a wife and mother.

**Conclusions**

Adolescent unwanted pregnancies are met with a range of responses. Partners and parent’s responses influence the pregnancy adolescent on decision making for continuation or termination of pregnancy, which furthermore will affect adolescent health status.

## O53 Antenatal care satisfaction according to perspective of patient and midwives in Kronjo Public Health Center, Tangerang, Banten 2017

### Rini Kundaryanti^1^, Nursyirwan Effendy^2^, Masrul^2^, Asmawi^2^

#### ^1^Faculty of Public Health, Universitas Nasional, Jakarta Selatan, 12520, Indonesia; ^2^Faculty of Social and Political Science, Universitas Andalas, Padang, 25163, Indonesia

##### **Correspondence:** Rini Kundaryanti (rinik74@gmail.com)

**Background**

Antenatal care satisfaction is the main objective of antenatal care (ANC) in *Puskesmas* (public health center). Perceived complain from patient become indicator and as the main point to measure satisfaction. Many factors influence antenatal care satisfaction both from midwives and patient. This study was conducted to identify the quality of antenatal care from the perspective of patients (waiting time, attitude, and facilities) and midwives (knowledge and skill) in *Puskesmas*.

**Materials and methods**

This was a quantitative study with 11 midwives and 110 pregnant women who were selected through purposive sampling. Data analysis was conducted with smart PLS (Partial Least Square) 2.0.

**Results**

Findings showed that knowledge and skill had direct significant relation towards quality and satisfaction whereby. Furthermore, it was found that patient’s perspective explained positive significant correlation on facilities (t = 4,64) and attitude (t = 2,65). Nevertheless, waiting time (t = 1,69) showed negative significant correlation.

**Conclusions**

Satisfaction of antenatal care in *Puskesmas* influenced by skill and knowledge of midwives and furthermore associated with facilities and attitude. Waiting time had no correlation with satisfaction. Midwives should improve knowledge and skill to provide better and excellent antenatal care in Puskesmas. Moreover, patient evaluation is needed to improve satisfaction of antenatal care.

## O54 Quality of life assessment among gender dysphoria in Selangor, Malaysia

### Rishmarajeswari Rajah, Neni Widiasmoro Selamat

#### Faculty of Health and Life Sciences, Management and Science University (MSU), Shah Alam, Selangor, 40100, Malaysia

##### **Correspondence:** Neni Widiasmoro Selamat (neni_widiasmoro@msu.edu.my)

**Background**

People with gender dysphoria experience many difficulties which affect their quality of life (QoL) as they have been isolated and living their life without support from the society.

**Materials and methods**

A cross sectional study was conducted utilizing Gender Dysphoria Quality of Life Questionnaire (*Health Indicator*: 12 items; *Relationship Indicator*: 6 items; *Employment Indicator*: 4 items; *Social Indicator*: 8 items). SPSS version 23 was employed for data analysis.

**Results**

A total number of 45 participants with gender dysphoria (median age = 28 years, IqR = 5; male to female = 100%; Hindu = 53.3%; single = 80%; unemployed = 17.8 %; secondary school = 44.4%; urban = 93.3%; monthly income below RM1501 = 33.3%) were recruited. Overall, respondents demonstrated poor QoL (*Health Indicator:* median = 15, IqR = 2.50; *Relationship Indicator*: median =10, IqR = 1.50; *Employment Indicator*: median = 4, IqR = 1.50; *Social Indicator*: median = 11, IqR = 2). Majority of the respondents have diabetes mellitus (28.6%), on 935 Andrcord hormone therapy (28.6%) and had undergo metoidioplasty surgery (43.9%). There is no significant correlation between QoL on gender dysphoria based on demographic characteristics *(p>0.05).*

**Conclusions**

Worse QoL particularly in health, relationship, employment and social indicators has no association with demographic variables. Therefore, healthcare providers should be more sensitive to the healthcare needs of gender dysphoria to improve their psychosocial wellbeing.

## O55 The association of culture perception with chronic energy malnutrition in women of childbearing age in Terbanggi Besar, District of Central Lampung, Indonesia

### Dian Isti Angraini, Diana Mayasari, Reni Zuraida, Sofyan Mussabiq Wijaya

#### Department of Community Medicine and Public Health, University of Lampung, Bandar Lampung, 35141, Indonesia

##### **Correspondence:** Dian Isti Angraini (riditie@gmail.com)

**Background**

Prevalence of chronic energy malnutrition (CEM) among women of childbearing age (WCA) in Indonesia and Lampung Province in 2013 were 20,8% and 17,6% respectively. CEM among WCA reflecting poor nutrition status in the past may increase the risk of complication in pregnancy and childbirth. The objective of this study was to determine the association of culture perception with CEM in WCA in Terbanggi Besar, District of Central Lampung, Indonesia.

**Materials and methods**

This was an observational analytic study using cross sectional design. The study was conducted in Terbanggi Besar subdistrict, District of Central Lampung from August to November 2016. Samples were 183 WCA aged 20 to 45years, selected by cluster sampling. Chronic energy malnutrition was measured with mid-arm circumference, whereas culture perception (early marriage, parity, race, food taboo) were collected by questionnaire. Data was analyzed using chi square test.

**Results**

The results showed that 44,3% respondent suffered from CEM, 15,8% were lampungnese, 71% were married at young age, 38,8% were multipara and 71,6% had food taboo. Early marriage and multipara were significantly associated with CEM, while race and food taboo were not associated with CEM.

**Conclusions**

There was a significant relationship between early marriage and multipara with CEM among WCA in Terbanggi Besar, District of Central Lampung, Indonesia.

## O56 Determinants of unmet need for family planning in Salatiga

### Sri Winarni^1^, Yudhy Dharmawan^1^, Najib^2^

#### ^1^Department of Biostatistics and Population study, Public Health, Diponegoro University, Semarang, 50275, Indonesia; ^2^National Population and Family Planning Board, Semarang, 50275, Indonesia

##### **Correspondence:** Sri Winarni (wiwin.undip@gmail.com)

**Background**

Central Java provinces has high unmet need rate of family planning at 7,6%. In 2015, Salatiga city has the highest unmet need rate in Central Java (13,3%). This research aimed to analyze the determinants of unmet need of family planning in Salatiga city.

**Materials and methods**

This research was a cross sectional study. This study was conducted in rural area (Argomulyo) and urban area (Sidomukti) with high unmet need rate. Population of this study was 15,081 women of fertile couples in Argomulyo and Sidomukti sub-districts. This study involved 200 respondents selected by proportional random sampling.

**Results**

Significant predisposing factors of unmet need were a norm in the community that many children lead to family welfare (p= 0,0001), education of respondents (p=0,026), perceptions of contraceptive effects (p=0,0001), and age (p=0, 0002). It was found that significant enabling factors were exposure to media (p=0,009) and exposure to electronic media (p=0,001). Significant reinforcing factors of unmet need were husband support (p=0,0001) and feelings of sin when use contraception (p=0,0001). In addition, unmet need was also related with fear of its side effects (33,3%) and husband is not at home (18,2%).

**Conclusions**

Knowledge related to contraceptive side effects of FP was still low and understanding of the respondents about the myths of family planning needs to be corrected. Increased education and knowledge of family planning was needed. This study suggests that family planning education could be delivered through media or channeled by religious leaders.

## O57 Factors associated with fatigue in breast cancer patient

### Ismarina, Delmi Sulastri, Rizanda Machmud, Afriwardi

#### Public Health Department, University of Andalas, Padang, 25127, Indonesia

##### **Correspondence:** Ismarina (rinaismarina77@gmail.com)

**Background**

Cancer is one of the death causes in the worldwide. Therapy has been provided by health provider to reduce pain and only 5% of therapy are focus to reduce fatigue from cancer. More than 80% of breast cancer patients has an experiences on fatigue before, during, and after therapy. Fatigue due to breast cancer still received less attention. This study aimed to determine the associated factors of fatigue in breast cancer patient.

**Materials and methods**

This study employed cross sectional design. A total of 122 respondents with breast cancer in national cancer hospital Jakarta were selected through simple random sampling method. Data was collected by using questionnaire and secondary data was also collected. Data was analyzed by using logistic regression models.

**Results**

The prevalence of fatigue was 44,3% for light until moderate fatigue and 55,7% for severe fatigue. The associated variables with fatigue were education, stadium of breast cancer, depression level, and sports activities which involved in the best model of logistic regression analysis. The most important variable was depression level. This research found five confounders including basic information about breast cancer, difficulty of breathing, management of symptoms, communication with the doctor, and management of depression.

**Conclusions**

Severe fatigue was related to education, stadium of breast cancer, depression level, and sports activities. In order to prevent severe fatigue among breast cancer patient, depression level should be minimized and sport activities should be enhanced.

## O58 Using administrative data to develop cervical cancer policy and planning under Universal Coverage Scheme in Thailand

### Suwaporn Marsook^1^, Ngamphol Soonthornworasiri^2^

#### ^1^National Health Security Office, Bangkok, 10210, Thailand; ^2^Mahidol University, Nakhon Pathom, 73170, Thailand

##### **Correspondence:** Suwaporn Marsook (suwaporn.m@nhso.go.th)

**Background**

Administrative data, also known as claims data is huge health information, easily available, and cover large service of patients’ data but does not provide clinical details. For the health policy department as National Health Security Office, it is necessary to use this data for monitoring, and evaluation for improving the quality of patient care.

**Materials and methods**

We would like to use administrative data fiscal year between 2010-2014 for clinical research in order to find the survival of cervical cancer patients under the Universal Coverage (UC) scheme and associated factors of the survival rate of cervical cancer patients using two techniques which are data mining decision trees and Cox proportional hazard model. The outcome of this study will benefit to developing cervical cancer policy and planning such as specialty healthcare, workforce, etc. to increase accessibility to healthcare facility in the future. Outcome from this study will be applied in related policy development process in order to promote accessibility and quality of service related to cervix cancer.

**Results**

This study found survival curve of cervical cancer patients who have history of screening by Pap smear higher than who do not have. For finding associated factor of the survival of cervical cancer patients using data mining decision trees and Cox proportional hazard model found history of Pap smear is the most influence on survival.

**Conclusions**

Using administrative data may be suitable for forecasting or direction trend of health management to improve health service at the country level. On the other hand, at the health care units, using medical records which include more detailed clinical information is the most benefit the development of truly service quality.

## O59 The improved coverage of cervical-cancer screening with QOF indicator

### Saray Ruangdej, Kajeeratn Prugaego, Sirirat Wongprakornkul, Bumrung Chalodech, Wilasinee Salelanont

#### National Health Security Office, Bangkok, 10210, Thailand

##### **Correspondence:** Saray Ruangdej (saray.r@nhso.go.th)

**Background**

The Universal Health Coverage Scheme has provided free cervical cancer screening policy for all Thai female aged 35 to 60 years. The purpose is to reduce the cervical cancer mortality by prevention and early detection. The cervical cancer screening coverage of target group within five years has been throne of the key quality outcome framework (QOF) indicators for on-top payment since 2014.

**Materials and methods**

NHSO’s individual administrative data in 2014 and 2017 sent by 1,301 Contracting Units of Primary Care (CUPs) were used. Descriptive statistics and Wilcoxon Signed Ranks Test were applied.

**Results**

The national coverage of cervical screening was significantly increased from 28.60% to 36.68%. It was found that 97.30% of CUPs showed their improvement even though there were very much different among them.

**Conclusions**

Using QOF indicator led to cervical cancer screening coverage improvement. Therefore, it should be continued using this indicator together with developing service network as well as raising awareness and also promoting the right to receive the service free of charge under the UC’s benefit package.

## O60 Analysis of factors affecting *puskesmas* performance: a case study of health center in national health insurance era

### Evi Derma Sastiva, Afrizal, Hardisman, Hefrizal Handra

#### Public Health Department, Faculty of Medicine, Andalas University, Padang, 25128, Indonesia

##### **Correspondence:** Evi Derma Sastiva (evidermas@gmail.com)

**Background**

Indonesian National Health Insurance (JKN) has been implemented since January 1, 2014. The study aimed to analyze the factors that affect poor quality of *puskesmas* (public health center) in JKN era.

**Materials and methods**

The research used qualitative method to examine the natural condition of the object. Triangulation was conducted. This study was conducted in CHC I group consisted 3 units of *puskesmas* with low Minimum Service Standard (MSS) and CHC II group consisted 2 units *puskesmas* with high MMS.

**Results**

Input, process, output, environment, leadership, management, and organizational work culture in CHC II were performed better than CHC I. Better medical service was found in CHC II. Environment in the form of government policy and local government were better in CHCII. Leadership, management and culture of organizational work were better in CHC II. Output of public health target indicators in the form of MSS in CHC II was slightly better than in CHC I. It was found that *puskesmas* performance affected by sufficient human resource, funds, and facilities availability.

**Conclusions**

It can be concluded that human resource, funds, and facilities in public health center influenced its performance in JKN era.

## O61 Potential fraud of BPJS claims at Tenriawaru Public Hospital of Bone Regency, Indonesia

### Sukri Palutturi^1^, Siti Rahmawati Makkurade^2^, Reza Aril Ahri^2^, Ade Suzana Eka Putri^3^

#### ^1^School of Public Health Hasanuddin University, Makassar, 90245, Indonesia; ^2^School of Public Health Muslim University of Indonesia, Makassar, 90121, Indonesia; ^3^School of Public Health, Andalas University, Padang, 25128, Indonesia

##### **Correspondence:** Sukri Palutturi (sukritanatoa72@gmail.com)

**Background**

National Health Insurance scheme to meet the target of universal health coverage in Indonesia is still in its initial phase of implementation. Potential fraud that is disadvantageous to patients and others is possible to be found. The aim of this study was to obtain in-depth information about the potential fraud of health care claims to the Social Health Security Agency (BPJS) in Tenriawaru Public Hospital of Bone regency, Indonesia.

**Materials and methods**

This study was a qualitative research with descriptive analysis. Informant was selected by purposive sampling. Data collection techniques employed in this study were interview, observation and documentation. Data analysis was done descriptively and data validity used triangulation of data source.

**Results**

The results showed that there was a potential fraud that occurred at Tenriawaru Regional General Hospital. It was caused by health care providers including health workers and coders. This study identified eight types of potential fraud, namely up-coding, readmissions, type of room charge, unnecessary treatment, phantom billing, keystroke mistake, service unbundling of fragmentation, and cancelled service. This regulation has included elements of fraud and the types of potential fraud that occurs in primary health care and referral health.

**Conclusions**

Eight potential fraud was found in in Tenriawaru Public Hospital of Bone regency, Indonesia. This research recommends the government to develop rules preventing potential fraud that may occurs in primary health care and referral health facilities, and give deterrent effect for fraud perpetrators as well.

## O62 The obedience in paying premium and the health service quality received by BPJS patients in Haji General Hospital, Makassar City, Indonesia

### Darmawansyah, Chaerunnisa Agus Ronrong

#### School of Public Health Hasanuddin University, Makassar, 90245, Indonesia

##### **Correspondence:** Darmawansyah (darmawansyah1964@gmail.com)

**Background**

This study aimed to analyze the factors affecting the obedience to pay and the health service quality among *BPJS Mandiri* patients, the voluntary members of JKN (Indonesian National Health Insurance) in Haji General Hospital, Makassar City, Indonesia.

**Materials and methods**

This research employed quantitative method with cross sectional design to determine factors affecting the obedience of paying JKN premium and to find the correlation between obedience to pay and health service quality among *BPJS Mandiri* patients in Haji General Hospital, Makassar City.

**Results**

The research found that knowledge, the social class, past experience, the family support, affected the obedience to pay and there was correlation between the obedience to pay (and health service quality. The result of multiple logistic regression simultaneously showed that the knowledge had the greatest effect on the obedience of *BPJS Mandiri* patient to pay.

**Conclusions**

The study described the obedience of paying influenced by knowledge, social class, past experience and family support. There was correlation between the obedience to pay *BPJS Mandiri* patient and health service quality at Haji General Hospital, Makassar City.

## O63 The determinants of willingness to pay JKN Premium for third class ward service among non-subsidized people in Sawahan Timur Sub-district, Padang City

### Adila Kasni Astiena (adila.kasni@yahoo.com)

#### School of Public Health, University of Andalas, Padang, 25148, Indonesia

**Background**

Indonesian National Health Insurance (JKN) was launched on January 1, 2014. Most of families in Sawahan Timur sub-district comes from middle to lower economic level. Their inadequate income causes them to be unable to pay JKN premium. The purpose of the study was to determine the determinants of willingness to pay the lowest JKN premium among the community of Sawahan Timur sub-district, Padang City.

**Materials and methods**

The design of this research was quantitative analytic with cross sectional design. The dependent variable was willingness to pay. The independent variables were age, education level, income, ability to pay ATP1 and ability to pay ATP2. The study was conducted from December 2013 to February 2014. The samples were taken proportionally by random sampling with total 101 people from the population of Sawahan Timur sub-district. The data were analyzed by using chi square test.

**Results**

The results showed that most of the respondents were traders (34%), 34-44 years old (34%), junior high school (32.7%), length of stay in Sawahan Timur more than 10 years (73.3%), 56.4%), self-owned house (49.5%), family size of three people (58.4%). Most of them (76.2%) had income which is equal to or exceed provincial minimum wage, with preferred health providers were private clinic, ATP1 (37,6%), ATP2 (84,2%). Most respondents (81.2%) were willing to pay JKN premium. It showed that there was relationship between the level of education, ability to pay (ATP1 and ATP2) and the willingness to pay, whilst it was not related to age, income and occupation.

**Conclusions**

Willingness to pay was related to level of education and ability to pay (ATP1 and ATP2). This study suggests the government to more consider on public ability to pay in determining the JKN premium. In addition, government should subsidize the non-subsidized people who are unable to pay JKN premium.

## O64 Factors related to willingness of non-wage workers to pay national health insurance fee in Batang Kabung, Koto Tangah, Padang City in 2015

### Syafrawati (syafrawati@gmail.com)

#### Health Policy and Administration Department of Public Health Faculty, Andalas University, Padang, 25148, Indonesia

**Background**

Koto Tangah is a district in Padang City with the highest number of populations which have not registered yet as the participant of national health insurance in 2015. Batang Kabung Ganting is a village with the highest number of households, most of them are non-wage workers and without national health insurance coverage (89.4%). The purpose of this study was to find out the factors related to the willingness of non-wage workers to pay national health insurance premium.

**Materials and methods**

This research used cross-sectional study design with 94 samples of households and proportional random sampling was employed. Data were analyzed by using chi square test at 95% confidence interval.

**Results**

The result showed 57.1% respondents declare that they did not want to pay health insurance fee, 58.2% of respondents were 20 to 49 years old, 61.2% of respondents did not have children under five years old or elderly in their family, 77.6% of respondents had no ill experience, 74.5% of respondents were able to pay national health insurance fee based on ATP1, and 72.4% of respondents were able to pay national health insurance fee based on ATP2. There was no significant relationship between willingness to pay and age, the experience of illness and ATP 2. There was significant relationship between willingness to pay and the existence of children or older people in family and ATP1.

**Conclusions**

There was significant relation between the willingness to pay with the existence of children or older people in family and ATP1.

## O65 Correlation between public health service and satisfaction level of JKN beneficiaries in Pratama Kota Clinic, Samarinda City

### Dewi Astuti, Subirman, Siswanto

#### Public Health Faculty, Mulawarman University, Samarinda, 75119, Indonesia

##### **Correspondence:** Dewi Astuti (dewiastt21@gmail.com)

**Background**

*Pratama Clinic* is a primary health facility contracted by BPJS, the National Health Security Agency of Indonesia for National Health Insurance (JKN) beneficiaries. A qualified healthcare is imperative to attain patient's satisfaction. Based on an initial study in five *Pratama* clinics, patients mentioned that there has been a lack of hospitality, tardiness of doctors, overlong waiting-time, narrow waiting-room, and biased health information. The condition may indicate a lack of healthcare quality in *Pratama Clinics*. The aim of this study was to examine the correlation between quality of health care and satisfaction level of patients of JKN in Pratama Kota Clinic, Samarinda City.

**Materials and methods**

The research was conducted in March 2017, which took place in six *Pratama* clinics in Samarinda City. Analytic survey using cross-sectional approach was employed. Samples of this study were JKN beneficiaries who utilized healthcare in these six *clinics*, as many as 357 respondents. The sampling method was multi-stage sampling, and Rank Spearman Correlation test was used.

**Results**

The result showed that there was no correlation between technical competence with patient's satisfaction. This study found that there was correlation between healthcare, human-relation, and convenience with patient's satisfaction.

**Conclusions**

As the matter of fact, *Pratama* Clinic is suggested to minimize waiting time for patients to register, maximize consultation duration related to the illness that patients suffer from, and set a comfortable situation in waiting room to gain patients’ satisfaction.

## O66 Drug availability in Salido Public Health Center, Pesisir Selatan District, Indonesia

### Sri Siswati, Gusti Fauzi

#### Health Police Administration, Andalas Univerity, Padang, 25148, Indonesia

##### **Correspondence:** Sri Siswati (srisiswati@yahoo.co.id)

**Background**

In the era of JKN (Indonesian National Health Insurance), the increase in the number of patients leads to medicine shortage. Salido PHC, which in 2015 received an award of the best head of PHC in Indonesia, faced lack of drug availability to more than 50% from its requirement.

**Materials and methods**

This research was a descriptive research with qualitative design. The study was conducted in Salido PHC in November 2016 to April 2017. The samples were taken purposively. Data was collected through observation, in-depth interview among staffs and documentation.

**Results**

This study found that Medicine Need Plan has been done, however standard operating procedure of rational treatment was not found. Salido PHC has autonomy to purchase of medicines but are not available in Pharmaceutical Wholesalers. Local government aid drugs are also long-standing for coming. Replacement with patent medications, results in greater budget spending and the patient's impact, such as the patient should buy himself a drug that can not be given, or the patient is referred to the hospital to overcome it. The unavailability of drugs for 144 diseases is beyond the capacity of the Public Health Center. Many public and private health services facilities have participated in JKN Program.

**Conclusions**

It is recommended that private health service facilities in cooperation with BPJS should also develop a Medicines Needs Plan, send to health organization district and province. We hope the factories or principals can produce the right medicines. Many staffing must be capacity building about medicines plan. It needs commitment to implement JKN policy to prevent drug void in Community all Public Health Centers, Clinics and hospitals.

## O67 Relationship between the readability of emergency form with the accuracy on the external causes coding of traffic accident injury

### Nandita Risa Ramadhani, Nuryati

#### Department of Health Services and Health Information, Vocational College, Universitas Gadjah Mada, Yogyakarta, 55281, Indonesia

##### **Correspondence:** Nuryati (nur3yati@ugm.ac.id)

**Background**

RSU PKU Muhammadiyah Bantul is one of the referral hospitals in Yogyakarta Province, Indonesia. Based on the earlier study of 10 medical records of inpatients with traffic accidents, accuracy was found in 40% of medical records. It was also found that the percentage of readability in emergency form was 90%. The objective of this research was to analyze the relationship between the readability of the information in emergency form with the accuracy of the external cause coding of injury among traffic accident cases in RSU PKU Muhammadiyah, Bantul.

**Materials and methods**

This research was conducted by survey with quantitative approach. The samples of this research was 54 medical records of inpatients with traffic accident during February to March 2016. Sampling was done by quota sampling method. Pearson’s chi-square test was used for data analysis.

**Results**

The percentage of readability in emergency form was 92,6%. The percentage of the accuracy of external cause coding among cases with injury due to traffic accident was 14,8%. This study found that readability in emergency form was related with the accuracy of external cause coding (p-value = 0,039).

**Conclusions**

There was a relationship between the readability of the information in emergency form with the accuracy of the external cause coding of injury among cases with traffic accident in RSU PKU Muhammadiyah Bantul.

## O68 Prolonging micronutrients supplementation in 2-6 months prior to pregnancy significantly improves birth weight by modifying maternal weight gain rate: a randomized double blind community-based trial

### Sri Sumarmi^1^, Soenarnatalina Melaniani^2^, Kuntoro^2^, Bambang Wirjatmadi^1^

#### ^1^Department of Nutrition, Faculty of Public Health, Universitas Airlangga, Surabaya, 60286, Indonesia; ^2^Department of Biostatistics and Population Study, Faculty of Public Health, Universitas Airlangga, Surabaya, 60286, Indonesia

##### **Correspondence:** Sri Sumarmi (msrisumarmi@gmail.com)

**Background**

Nutrition intervention in very early pregnancy is important to support the successful of scaling up nutrition (SUN) movement to safe the first 1000 days of life. Micronutrients supplementation prior to pregnancy is required to reach this critical period and improve pregnancy outcomes. This research was conducted to evaluate the effect of prolonging multi-micronutrients supplementation 2-6 months prior to pregnancy on maternal weight gain and birth weight.

**Materials and methods**

A randomized double blind community-based trial was conducted at District of Probolinggo East Java, Indonesia. A two-arms study consists of group I that received placebo before pregnancy and continued with daily iron and folic acid (IFA) during pregnancy, and group II received multi-micronutrient containing 15 micronutrients, 2 other days before pregnancy, and continued with daily dose during pregnancy. A sample size of 115 pregnancy outcomes was obtained from 420 brides-to-be. The primary outcome variables were maternal weight gain and birth weight. Socioeconomic status and maternal body mass index were assessed as base line data. Data were analyzed using MANCOVA and ANCOVA to evaluate effect of supplementation. General linear model was used to test the homogeneity between two groups.

**Results**

Results showed that the characteristic between treatment group and control group were not different (Box’s M value p = 0.398; Hotelling's Trace value p = 0.478I). Birth weight of babies delivered by mothers who supplemented with multi-micronutrients 2-6 months prior to pregnancy were significantly higher compared to babies delivered by mother who received iron-folic acid supplementation during pregnancy (Δ= 309.89 g; p=0.000). Total maternal weight gain does not difference between two group (p=0.995), otherwise descriptively there was differentiation pattern of weight gain rate between two group.

**Conclusions**

It implies that prolonging supplementation of multi-micronutrient 2-6 months prior to pregnancy improves birth weight by modifying the pattern of maternal weight gain rate.

## O69 The correlation between knowledge, attitude, and types of childbirth and Early Initiation of Breastfeeding (EIB) at Ria Kencana Hospital Maternity of PKBI Samarinda

### Lia Kurniasari, Ainur Rachman, Muhammad Argobi

#### Universitas Muhammadiyah Kalimantan Timur, Samarinda, Indonesia

##### **Correspondence:** Lia Kurniasari (sri.sunarti@gmail.com)

**Background**

Indonesia needs to improve the quality of human resources, especially in the field of health which focuses on the improvement of children and mother’s health by accelerating the reduction of children mortality rate (for babies and children under five years) and the decrease of maternal mortality rate. This study aimed to find out the correlation between knowledge, attitude, and the types of delivery and the early initiation of breastfeeding (EIB) at Ria Kencana Maternity Hospital of PKBI Samarinda.

**Materials and methods**

The design of this research was an analytic survey with a cross-sectional approach.

**Results**

It was found that knowledge, attitude and the types of delivery influenced the early initiation of breastfeeding at Ria Kencana Maternity Hospital of PKBI Samarinda.

**Conclusions**

There was a correlation between knowledge, attitude, types of delivery and early initiation of breastfeeding (EIB) at Ria Kencana Maternity Hospital of PKBI Samarinda. Midwives need to initiate early initiation of breastfeeding (EIB) and make mothers to be confident for early breastfeeding either for normal or cesarean delivery.

## O70 Energy, protein intake of maternal and economic factor as determinants of birth weight: a prospective study

### Azrimaidaliza^1^, Kusharisupeni^2^, Abas Basuni^3^, Diah Mulyawati Utari^2^

#### ^1^Public Health Faculty, Andalas University, Padang, 25147, Indonesia; ^2^Nutrition Department, Public Health Faculty, Indonesia University, Depok, 16424, Indonesia; ^3^Applied Technology Center for Health and Clinical Epidemiology, Depok, 25147, Indonesia

##### **Correspondence:** Azrimaidaliza (uniminda@yahoo.com)

**Background**

Birth weight less than 3000 grams is an important risk factor of childhood morbidity and may lead to degenerative disease in adulthood besides low birth weight. Several studies approved that inadequate nutrient intake on pregnant women and low economic status had an effect on birth weight. The aim of this study was to know the difference of birth weight based on maternal energy intake, protein intake, and economic status.

**Materials and methods**

Prospective cohort design was used to know the determinants of birth weight. Study sample was 202 pregnant women who were observed from early pregnancy until delivery. By using Independent t-test, Mann Whitney test, and simple linear regression, the determinants of birth weight was analyzed.

**Results**

The results showed that 41.3% of mothers delivered babies with birth weight less than 3000 grams, the mean of maternal energy intake was 1819.6 kcal (sd = 209.3 kcal), the mean of maternal protein intake was 65.7 grams (sd = 9.3 grams) and the mean of household expenditure was IDR 3,481,929 (sd = IDR 2,840,730). Maternal who delivered the baby with birth weight more than 3000 grams had better mean energy intake, protein intake and economic status during pregnancy than those who delivered the baby with birth weight less than 3000 grams. Maternal energy intake at third semester (month 9) has the impact to birth weight.

**Conclusions**

Birth weight was significantly different according to maternal energy intake, protein intake and economic factor during pregnancy.

## O71 Risk factors of chronic energy deficiency among pregnant woman in North Gorontalo District, Indonesia

### Rifa’i Ali, Zul Adhayani Arda, Sri Lestisya N. Jole

#### Faculty of Public Health, Gorontalo University, Gorontalo District, 96211, Indonesia

##### **Correspondence:** Rifa’i Ali (rifai_ali86@yahoo.co.id)

**Background**

One of the nutritional problems among pregnant women is Chronic Energy Deficiency (CED) and accompanied with the emergence of various health problems. The purpose of study was to determine the risk factors associated with chronic energy deficiency in pregnant women in North Gorontalo District in 2016.

**Materials and methods**

The study was an observational analytic with case control design. Case groups consisted of 345 pregnant women who experienced CED, and control group consisted of 345 pregnant women who did not experience CED. The data were analyzed by using logistic regression test.

**Results**

The study result showed that antenatal care (OR= 1.88; 95% CI: 1.39-2.56) and consumption of iron tablets (OR= 1.91; 95% CI: 1.28-2.85) were significant risk factors for Chronic Energy Deficiency (CED) among pregnant woman. Age in first pregnant and age of menarche were not significantly associated with CED among pregnant woman. The result of multivariate analysis showed that consumption of iron was mostly influence CED in pregnant women (Exp(B)= 1.81; 95% CI: 1.22-2.71).

**Conclusions**

It is recommended to the public especially in pregnant women to do early pregnancy checkup and regular consumption of tablets Fe.

## O72 Consumption of high glycemic index food and blood glucose level in patients with Diabetes Mellitus at Dr. M. Djamil Hospital, Padang City, Indonesia

### Hafifatul Auliya Rahmy^1^, Yosi Irene Putri^2^, Nur Indrawaty Lipoeto^3^

#### ^1^Faculty of Public Health, Andalas University, Padang, 25129, Indonesia; ^2^Faculty of Medicine, Diponegoro University, Semarang, Central Java, 50275, Indonesia; ^3^Faculty of Medicine, Andalas university, Padang, 25148, Indonesia

##### **Correspondence:** Hafifatul Auliya Rahmy (rauliyabee@gmail.com)

**Background**

Diabetes Mellitus (DM) is very related to behavior such as dietary habit. The consumption of meals with high of Glycemic Index (GI) can increase the amount of blood glucose level. The aim of this research was to know the correlation between the consumption of meals with high level of GI and blood glucose level in patients with diabetes mellitus at polyclinic of Dr. M. Djamil Hospital, Padang City, Indonesia.

**Materials and methods**

This cross-sectional study took 94 patients as samples who were chosen by using purposive sampling technique. Blood glucose level was measured through laboratory test and consumption of meals with the high GI was measured by using semi quantitative FFQ. Statistical tests used in this study was simple linear regression.

**Results**

This study found that the average of blood glucose levels was 186.68 mg/dl, consumption of carbohydrate was 518.44 gr/day, beverage consumption was 175.12 ml/day, and fruits consumption was 49.95 gr/day. The statistic test showed that there was a correlation between consumption of carbohydrate, beverage consumption, and fruits consumption with high GI (p value < 0.05) and blood glucose levels.

**Conclusions**

Consumption of carbohydrate, beverage, and fruits with high GI are correlated with blood glucose levels. We expect that nutritionists could educate DM patients regarding meals with high level of GI.

## O73 Effects of food supplementation and psychosocial stimulation in children under 2 years of age on their nutritional status in Indonesia (a follow-up study)

### Helmizar Agus^1^, Nur Indrawaty Lipoeto^2^, Fasli Jalal^3^

#### ^1^Department of Nutrition, Faculty of Public Health, Universitas of Andalas, Padang, 25129, Indonesia; ^2^Department of Nutrition, Faculty of Medicine, Universitas of Andalas, Padang, 25148, Indonesia; ^3^Post Graduate Program, University of Jakarta, Jakarta, 13220, Indonesia

##### **Correspondence:** Helmizar Agus (eelbiomed@gmail.com)

**Background**

Stunting remains a prominent global health problem which need great concern. Interventions initiated before 2 years of age can prevent stunting and greatly benefit nutritional status in the next period of life. The aim of the study was to evaluate the effect of food supplementation and psychosocial stimulation for children under 2 years in West Sumatra province, Indonesia.

**Materials and methods**

In this study, there were 160 children recruited through cluster random sampling. It was conducted from August 2016 to August 2017. Nutritional assessment was conducted after dividing the sample population into four groups according to the type of intervention received: 1) Food Supplementation (FS) group, 2) Psychosocial Stimulation (PS)group, 3) Food Supplementation and Psychosocial Stimulation (FS+PS) group, and 4) Control (CG) group. The data are analyzed based on one-way ANOVA and linear regression.

**Results**

The children showed the weight gain about 7.4±2.4kg (95% CI; 7.1 - 9.7). The height increase after follow-up was 30.7±5.2 cm (95% CI; 29.9 – 31.5). Significantly, differences were found in nutritional status of children based on height for age index, with an increase as large as 0.14. This was found in food supplementation and psychosocial stimulation group.

**Conclusions**

Combination of food supplementation and psychosocial stimulation called *manjujai* in local language had positive effect on linear growth and nutritional status after follow-up of the children.

## O74 The correlation between income level, attitude and smoking habit in family with nutritional status of children under five years old in Surabaya, Indonesia

### Lailatul Muniroh, Triska Susila Nindya, Farapti

#### Department of Nutrition, Faculty of Public Health, Universitas Airlangga, Surabaya, 60115, Indonesia

##### **Correspondence:** Lailatul Muniroh (lailamuniroh@fkm.unair.ac.id)

**Background**

Family income and smoking habits affect food security that furthermore will affect nutritional intake and nutritional status of the children. This study was aimed to analyze the relationship between family income, attitude and smoking behavior in the family with nutritional status of children under five years old in Surabaya.

**Materials and methods**

This research was an observational analytic, with cross sectional design. The population was children under five years old and their mothers in five sub-districts of Surabaya City, Indonesia. Multistage random sampling obtained 468 samples. Primary data were obtained by interviews using questionnaires, and weight and height of children were measured by using digital scales and microtoice. Data analysis used chi square test.

**Results**

The results showed that family income was in the range of IDR 400,000 to IDR 19,500,000 per month. Most of family income was above minimum salary of Surabaya (59.4%). There was 61.5% family members who smoked. Average cigarettes consumed was 9.5 sticks per day. Family attitude on smoking mostly showed a supportive attitude that smoking was a bad habit (93.9%). Nutritional status of children was mostly within normal category. There was a relationship between income level and family attitude about smoking with nutritional status of W/A index. There was no relationship of the existence of active smoker in the family with nutritional status of various indexes.

**Conclusions**

Increasing income and good attitude about smoking in the family can increase the nutritional status of children.

## O75 Adolescent’s knowledge on the dangers of glue addictive substance on VIII grade students of YPS Junior High School, Samarinda City, Indonesia

### Sri Sunarti, Riri Apriani, Nida Amalia, Fery Fadzul Rahman

#### Universitas Muhammadiyah Kalimantan Timur, Samarinda, Indonesia

##### **Correspondence:** Sri Sunarti (sri.sunarti@gmail.com)

**Background**

Adolescent is a risk group related to narcotics, psychotropic, and other addictive substances. Glue contains inhalation addictive substance product at low price and easy to find in all types of markets. Sucking flavor from glue is the way of glue addictive substance abuse. Sucking glue behavior can be due to lack of knowledge about the dangers of glue addictive substance. Thus, health education and information about the dangers of glue addictive substance through video for students is highly needed. This research was effect of health education through video to adolescent’s knowledge on glue addictive substances abuse on VIII grade students of YPS Junior High School, Samarinda City.

**Materials and methods**

This study was a mix-method research. The study used pre- and post-test one group design and the qualitative design. The sample was 75 students of VIII grade students of YPS Junior High School, Samarinda. Proportionate stratified random sampling technique was used for sample selection.

**Results**

Environment, peer, and ignorance related to the behavior of glue addictive substance abuse. There was a significant increase on knowledge after watching the video. The proportion of students with good knowledge increased was from 4% to 85.3%.

**Conclusions**

There was an influence of health education through video media toward adolescent’s knowledge on the dangers of glue addictive substances among VIII grade students of YPS Junior High School, Samarinda City.

## O76 The policy of addition of bitter substance Denatonium Saccharide (DSc) in formalin as the effort to prevent misuse from formalin to food chain

Wulan Puspita Puri (wulanpuspitapuri@gmail.com)

The misuse of formaldehyde in food is regrettable. Formalin has a function as an antibacterial agent that can slow the activity of bacteria in foods that contain lots of protein, therefore formalin reacts with proteins in food and makes food durable. However, when entering into the human body formalin is mutagenic and carcinogenic and trigger cancer cells and gene defects in the body. The Government through the Ministry of Trade as the regulator has issued Regulation of Minister of Trade Number: 75 / M-DAG / PER / 10/2014 regarding Second Amendment to Regulation of the Minister of Trade No. 44 / M-DAG / PER / 9/2009 on Procurement, Distribution and Control of Dangerous Materials Formalin / formaldehyde fall into the category of Hazardous Materials. In addition to the regulation of production and distribution conducted by the Ministry of Trade, the NADFC with the duties and functions of drug and food control also impose some regulation of supervision and education efforts related to the control of hazardous substances that are misused in food. Based on the results of NADFC supervision during 2013-2015 there was a decrease in food containing hazardous materials, but still found food or food containing hazardous materials, especially formalin. Through this paper will be discussed policy about formalin formalization through the addition Denatonium Sacharida (DSc). This policy is expected that misuse of formalin into the food chain does not occur. Supervision is not necessary in the distribution channel, and supervision is only done with a bitter test on food. With the addition of bitter substances, formalin will not be used in food because it will cause the food to be bitter and not sold.

## O77 Fish bars made of bilih fish flour (*Mystacoleuseus padangensis*) as zinc source for diabetic patient

### Deni Elnovriza^1^, Hadi Riyadi^2^, Rimbawan^2^, Evy Damayanthi^2^, Adi Winarto^3^

#### ^1^Public Health Faculty, Andalas University, Padang, West Sumatera, 25129, Indonesia; ^2^Department of Community Nutrition, Faculty of Human Ecology, Bogor Agricultural University, Bogor, West Java, 16680, Indonesia; ^3^Department of Anatomy, Physiology and Pharmacology, Faculty of Veterinary Medicine, Bogor Agricultural University, Bogor, West Java, 16680, Indonesia

##### **Correspondence:** Deni Elnovriza (denielnovriza@gmail.com)

**Background**

Bilih fish is one of the potential of local food of West Sumatera which has nutrient content, especially zinc. Therefore, Bilih fish can be used as an alternative sources of zinc in people with diabetes mellitus, which usually have low level of zinc. The aim of this research was to know nutrition content of fish bars made by different percentage of Bilih fish flour with the best acceptable organoleptic characteristic.

**Materials and methods**

This research used Randomized Complete Design (RCD). The percentage of addition of Bilih fish flour were A = 30%, B = 40%, C = 50% to total weight of rice flour and white glutinous rice flour. The data were analyzed by ANOVA at 95% confidence interval. The best treatment is determined by good nutrition content and acceptable organoleptic characteristic.

**Results**

Nutrition content of fish bars substituted with Bilih fish flour showed that protein content ranged from 17.303±0.638 - 22.750±0.864 (p=0.004); carbohydrate 32.060±0.299 - 40.573±0.790 (p<0.05); ash 3.340±0.286 - 4.437±0.425 (p=0.012) and zinc levels of 32.930±0.820 - 45.823±2.425 (p=0.001). Bilih fish flour addition treatment did not significantly affect fat content, and water and energy, with value respectively ranged from 10.700±0.435 – 11.987±0.735 (p=0.060); 28.010±1.075 – 28.278 ± 1.465 (p=0.823) and 327.120±9.009 – 327.807±8.657. Organoleptic analysis showed that all of organoleptic parameters except taste have no significant effect on fish bars made of Bilih fish flour, namely colors (p=0.699), aroma (p=0.169) and texture (p=0.264).

**Conclusions**

The best treatment is the addition of 50% Bilih fish flour of total weight of rice flour and glutinous rice flour. The fish bars contain 327.120 kcal energy; 22.75% protein; 11.987% fat; water 28.27%; ash 4.437%; zinc 45.823 ppm. Organoleptic characteristics are quite interesting colors; aroma and taste of fish rather strong with crunchy texture. It seems fish bars roasting technique should use optimal temperature and time in order to obtain snack with more optimal storage time.

## O78 Analysis of nutrients and solubility test of *Moringa oleifera* leaf from three Indonesian provinces

### Syahrial, Rimbawan, Evy Damayanti, Dewi Apri Astuti, Pipih Supjijah

#### Public Health Faculty, Andalas University, Padang, West Sumatera, 25129, Indonesia

##### **Correspondence:** Syahrial (Abilwawa@gmail.com)

**Background**

*Moringa oleifera* is a species of the monogeneric family of *Moringaceae*. *Moringa oleifera* called as *Kelor* in Indonesian language, is a high source of calcium and in other hand, nanotechnology will help calcium to be absorbed faster. The purpose of this study was to analyze the nutrients from *Moringa oleifera* both in the form of powder and in the form of nano and perform the soluble test.

**Materials and methods**

The aim of this research was to analyze the nutrients of *Moringa oleifera,* including vitamins and minerals in powder and nano-form. Moringa leaf powder is obtained from Betawi, Blora and Yogyakarta. Methods used were Particles Size Analysis (PSA) to determine the particle size is nano, AAS method for mineral analysis, HPLC method for vitamin analysis and solubility test.

**Results**

This study obtained that the particle size of leaf powder from Jakarta was the largest at 80 mesh compared with Yogyakarta at 100 mesh and Central Java at 500 mesh. Nano particles was made from Yogyakarta moringa leaf with 352.5 nm size. The results of proximate analysis were almost same, but in nano size there was a decrease of nutritional value. Higher vitamin content was found in Moringa leaves originating from Central Java with 119 mg Vitamin A, 309 mg Vitamin C, whilst vitamin D3 and B12 were not detected. Mineral contents were 355 mg calcium, 314.5 mg phosfor, 373.1 mg potassium, 446.5 mg magnesium in Yogyakarta Moringa leaf in the form of powder and insoluble nano particle.

**Conclusions**

The largest part of the powder particles was moringa leaf originated from Jakarta. The energy and vitamin C content was mostly found in moringa leaf originated from Central Java, while the mineral and other vitamins were almost no different in the three provinces.

## O79 The effect of stunting on the academic achievement of elementary school students in Tinggi Moncong District, Gowa, Indonesia

### Siti Sari, Sitti Gampang

#### Epidemiology Department, Faculty of Medical and Health Scince, UIN Alauddin Makassar, South Sulawesi, 92113, Indonesia

##### **Correspondence:** Siti Sari (hasnahhardiyanti7@gmail.com)

**Background**

Stunting is a deviation representing a long-term malnutrition. Stunting may affect the academic achievement of children caused by the non-optimal growth of the brain. In Indonesia and South Sulawesi the prevalence of stunting is 37.2% and 40.9%. These rates exceed the maximum prevalence standard of WHO that is 20%. This research was conducted to find out the stunted students frequencies and its effects on academic achievement in the minimal reach of health services as the related factor.

**Materials and methods**

This study was quantitative observational with cross sectional design. The location was at elementary schools in minimal reache of health services based on public health center of Tinggi Moncong preference. The samples of the study were the stunted students based on deviation standard z-score (72 students) and the tall students (15 students) who were measured by using WHO indicator. The data were students’ height measured by using microtoice, students’ mid test score and teachers interview score. The data were analyzed using Chi Square test.

**Results**

There were 10 students of 19 very stunted students with bad scores (52.6%). As many as 26 students of 55 stunted students with bad score (47.3%). There is no bad score among tall students.

**Conclusions**

There is a relation between stunting and academic achievement in the preference schools of minimal reaches of health services.

## O80 The effects of autogenic relaxation on blood glucose level among patients with Diabetes Mellitus Type 2

### Martalina Limbong, Pipin Sumantrie

#### Nursing Academy of Surya Nusantara Pematangsiantar, Sumatera Utara, 21101, Indonesia

##### **Correspondence:** Martalina Limbong (martalinalimbong@gmail.com)

**Background**

Autogenic relaxation could be expected to control blood glucose levels. The purpose of this study was to determine the effect of autogenic relaxation on blood glucose levels in patients with Type 2 Diabetes.

**Materials and methods**

Research design was quasi-experimental with pre- and pos-testt with control group, for each sample group consisted of 31 people obtained through consecutive sampling technique.

**Results**

Data were analyzed using Wilcoxon Sign-Range Test and U Mann Whitney. Analysis showed there was an effect of autogenic relaxation on blood glucose level decrease (p = 0.001).

**Conclusions**

There was significant autogenic relaxation to decrease blood glucose levels in patients with diabetes mellitus type 2. Results of this study can be an input for nursing services to conduct autogenic relaxation in providing nursing care to patients with DM Type 2.

## O81 A relationship between knowledge, attitude and practice about balanced nutrition guidelines and metabolic syndrome among central obese teachers in Makassar

### Nurzakiah^1^, Veni Hadju^2^, Nurhaedar Jafar ^2,^ Ridwan Thaha^2^

#### ^1^Public Health Study Program, Stikes Baramuli, Pinrang, Sulawesi Selatan, 91211, Indonesia; ^2^Faculty of Public Health, Hasanuddin University, Makassar, 90245, Indonesia

##### **Correspondence:** Nurzakiah (nurzakiahksruh@gmail.com)

**Background**

Metabolic syndrome prevalence is increasing in Asia Pacific. A balanced nutrition guideline has been developed by Ministry of Health in Indonesia and has been promoted. This study aimed to assess a relationship between knowledge, attitude, and practices about balanced nutrition guidelines and metabolic syndrome (Mets) among high school teachers with central obese.

**Materials and methods**

This was a cross-sectional study conducted in twelve high schools in Makassar city. Subjects were 131 teachers (28 men and 103 women) diagnosed with central obese. Knowledge, attitude and practice were assessed by trained field workers using a validated questionnaire. Mets was defined by measuring blood glucose, blood pressure, HDL, triglycerides, and waist circumference. Having three parameters or above were stated as Mets group and others were stated as risk group. KAP was categorized according to score and divided into low (Q1), moderate (Q2), and high (Q3). Chi-square test was used to assess the relationship.

**Results**

Subjects were mostly women (78.6%), Bugisnesse ethnic (66.4%), and married (96.2%). The prevalence of Mets was 39.5%, (57.1% were men and 34.7% were women). KAP was distributed evenly to three categories for each variable. The relationship between knowledge and Mets was borderline significant (p=0.093). There was no significant difference between attitude and Mets (p=0.406). However, there was significant difference between practice and Mets (p=0.016). Majority of respondents in the Mets group were in the high knowledge about balance nutrition guidelines and moderate practices (51.0% and 51.2%) compared with the risk groups that were mostly in moderate knowledge and low practice (71.1% 76.6%). These adequate knowledge and practice were higher in Mets group compared to Risk group.

**Conclusions**

There was relationship between knowledge and practice on balanced nutrition guidelines with Mets. Better knowledge and practice in Mets group because the patients followed health worker instruction even it should be still encouraged.

## O82 Analysis of long waiting time for quality improvement in health care in Adnaan WD Public Hospital Payakumbuh, Indonesia

### Isniati, Irsyadi Fauzan Afif

#### Faculty of Public Health, Andalas University, Padang, West Sumatera, 21528, Indonesia

##### **Correspondence:** Isniati (yetisniati@yahoo.com)

**Background**

Adnaan WD Hospital got a lot of negative response from patients because of long waiting time which was more than the 60 minutes. This study aimed to patients’ waiting time and its cause in Adnaan WD Hospital.

**Materials and methods**

This study combined quantitative and qualitative research methods. Quantitative research by counting patients’waiting time was conducted to 60 patient samples through observation and using stop watch. Qualitative research was conducted to 6 people to find out the causes of long waiting times through interviews.

**Results**

The research showed that there were 50 patients (83.3%) reported long waiting time. It caused by several factors namely the lack of health personnel at the registration table and medical record, indiscipline of the staffs and their performance has not been done in accordance with SOP, lack of facilities and infrastructure such as internet network problem, computer, printer, and late arrival of the doctors.

**Conclusions**

Based on the result, it can be concluded that average of the long waiting time was 2 hours 10 minutes which is more than standard. It was caused by problems in every point of registration service.

## O83 Health facilities assessment following the September 2009 West Sumatra earthquake

### Defriman Djafri (defrimandjafri@fkm.unand.ac.id)

#### Department of Epidemiology & Biostatitstics, Faculty of Public Health, Andalas University, Padang, 25147, Indonesia

**Background**

On September 30, 2009, an earthquake of moment magnitude 7.6 on the Richter scale occurred off the coast of Padang in West Sumatra province, Indonesia. Seven hospitals, 19 community health centers, and 38 supporting community health centers were damaged. Details of the recovery phase and the impact on health services were, however, lacking.

**Materials and methods**

A health facility-based situation review was conducted at 26 health facilities from November 2010 to May 2011 in Padang city to assess the damage and speed of recovery of the facilities.

**Results**

Two of them were severely damaged so that the activities had to be carried out in a nearly camp. Two hospitals and 6 community health centers were moderately damaged. Only outpatient service activities were available. Ten community health centers and 4 hospitals were mildly damaged. Services were maintained with minor limitations. Two health facilities were undamaged. Electricity was blacked out in forty per cent of facilities in the first week. The system was backed up by generator in almost one half of all service facilities. Only at the end of the third month did all facilities have a full electricity supply. Initial disruption of water supply was worse. More than a half of the centers were without water supply in the first week.

**Conclusions**

All these data suggested that in disaster prone areas, health facilities should be specially built or renovated to prevent damage. Humanitarian aid can shorten the recovery period of health services and should therefore be fostered.

## O84 Differences between patient safety at accredited public health center and non-accredited community health center in Padang City

### Dien Gusta Anggraini Nursal^1^, Rizanda Machmud^2^, Eryati Darwin^2^, Nana Mulyana^3^

#### ^1^Andalas University Faculty of Public Health, Padang, 25147 Indonesia; ^2^Andalas University, Faculty of Medicine, Padang, 25147 Indonesia; ^3^Ministry of Health Indonesian Republic, Jakarta, 12950, Indonesia

##### **Correspondence:** Dien Gusta Anggraini Nursal (diennursal@fkm.unand.ac.id)

**Background**

Patient safety is one of public health center (PHC) accreditation indicators. There was no reported of adverse event in PHC yet, it can be assumed that patient's safety had been good, however, many incidents in PHC was reported by mass media. This study aimed to determine the differences of patient safety between accredited and non-accredited PHC.

**Materials and methods**

This research used quantitative method with cross sectional study design. The study was held at two accredited PHC and two non-accredited PHC. Thirty health workers have been given validated questionnaires based on Malcolm Baldridge framework. SEM analysis was done.

**Results**

The result showed the average score of each variable in accredited PHC was higher than non-accredited PHC. Variables related to patient safety were leadership, policy, incident detection, mitigation, patient satisfaction, patient commitment, risk grading, RCA and FMEA, staff workload, staff commitment and internal audit. Factors related to patient safety in non-accredited PHC were incident detection, patient commitment, risk grading, and internal audit.

**Conclusions**

It can be concluded that patient safety at accredited PHC was better than non-accredited PHC.

## O85 Rational drug use and outcomes of respiratory diseases in the district hospitals under the Universal Coverage Scheme, Thailand

### Jutatip Thungthong^1^, Jatica Rattanadadas^2^, Cherdchai Soontornpas^3^, Thananan Ratanachopanich^4^, On-anong Waleekhachonloet^4^, Chulaporn Limwattananon^3^, Supon Limwattananon^3^, Noppakun Thammatacharee^5^, Kanjana Sirikomol^1^, Jiraphan Jaratpatthararoj^1^, Ninutcha Paengsai^1^, Amornrat Ngowabunpat^1^, Kunakorn Aewsuwan^1^, Wanna Eiadprapan^1^, Pongphan Ratchakom^1^, Wanida Jitvimonrat^1^, Jadej Thammatacharee^1^

#### ^1^National Health Security Office, Bangkok, 10210, Thailand; ^2^Master degree student, Faculty of Pharmaceutical Sciences, Khon Kaen University, Khon Kaen, 40002, Thailand; ^3^Faculty of Pharmaceutical Sciences, Khon Kaen University, Khon Kaen, 40002, Thailand; ^4^Faculty of Pharmacy, Mahasarakham University, Maha Sarakham, 44150, Thailand; ^5^Health System Research Institute, Nonthaburi, 11000, Thailand

##### **Correspondence:** Jutatip Thungthong (jutatip@nhso.go.th)

**Background**

The project of promoting of rational drug use (RDU) hospital has been implemented in Thailand since fiscal year (FY) 2014. This study aims to examine whether the RDU hospitals project could improve patient outcomes.

**Materials and methods**

Hospitalization data of the Universal Coverage Scheme (UCS) members which contained data of approximately 48 million populations and 3-4 million hospitalizations each year were analyzed between the RDU hospitals (N=160) and the non RDU hospitals (N=616) before (FY 2009 to 2013) and after implementation (FY 2014 to 2016). The RDU hospitals were further divided into phase I (N=46) and phase II RDU hospitals (N=114). Time series analysis was used. Outcomes included admission rate of asthma and COPD among population with aged at least 15 years, admission rate of URI among all population.

**Results**

From FY 2009 to 2016, trends of decreases in admission rate of asthma (80 to 70 per 100,000 population) and URI (54 to 31 per 100,000 population) were found. Trend of increase in admission rate of COPD (156 to 202 per 100,000 population) was observed. Based on a time-series analysis, admission rate of URI after implementation of the project among the non RDU hospitals increased significantly by 7.3 per 100,000 population annually while the rate among RDU hospitals was not significantly increased. Other outcomes did not statistically change after implementation of RDU hospitals.

**Conclusions**

The RDU hospital project has an effect in URI and asthma.

## O86 Accelerating admission flow in emergency department: identifying waste and resources by lean management approach

### Nurul Jannatul Firdausi^1^, Trisasi Lestari^2^, Agus Aan Adriansyah^1^, Kuncoro Harto Widodo^3^

#### ^1^Public Health Department, Health Faculty, University of Nahdlatul Ulama, Surabaya, 60237, Indonesia; ^2^Hospital Management, Public Health Department, Medical Faculty, Gadjah Mada University, Yogyakarta, 55281, Indonesia; ^3^Agroindustrial Technology, Agrocultural Technology Faculty, Gadjah Mada University, Yogyakarta, 55281, Indonesia

##### **Correspondence:** Nurul Jannatul Firdausi (nuruljf@unusa.ac.id)

**Background**

Delay in inpatient admission at Emergency Department (ED) is a daily challenge for a hospital and it is associated with longer length of stay and increase costs. The study aimed to explore the barriers of patient admission at ED by identifying waste and available resources.

**Materials and methods**

An exploratory case study using lean management approach was conducted at the ED and an internal medicine ward of Dr. Mewari’s hospital from March to July 2015. A total of 45 patient’s admission process from the ED to the internal medicine ward were observed. We measured the value of each activity, identified waste, compared available resources with demand and described the care process in a Value Stream Map (VSM). In-depth interviews with medical and non-medical staffs were conducted to explain the finding.

**Results**

The average total waiting time in ED was 183 minutes and 52 seconds. Eight types of waste, namely waste of waiting, motion, transportation, defect, over processing, over producing, inventory and human talent, were identified. Available resources and demands was unmatched.

**Conclusions**

Standard patient’s waiting time for admission from the ED was not achieved. Waste reduction and efficient use of resources for system improvement are required to accelerate patient’s flow.

## O87 Intervention program in efforts to improve compliance and blood pressure control of citizens in Kronjo Primary Health Center

### Rio Alexsandro, Prily Prianty, Rianty Febriandani, Novendy, Muhammad Fridzi Fikri

#### Public Health Department, Tarumanagara University, Jakarta,11440, Indonesia

##### **Correspondence:** Rio Alexsandro (alexsandrorio1993@gmail.com)

**Background**

There are 972 billion people with hypertension worlwide. As prevention measure, blood pressure control compliance is crucial for hypertension patients. In Kronjo Primary Health Center, a decrease of hypertension visits by 47% was reported. This study aimed to identify the cause of non-compliance issue regarding blood pressure control and develop a counseling method to address the problem.

**Materials and methods**

This research method was using Blum paradigm to find the cause. This study used quasi-experimental design. The respondents were health workers and patients. Samples were taken by consecutive non-random sampling from May to July, 2017. Interventions for 6 health workers were counselling skill development while education and motivation were conducted to 130 patients in 2 weeks. Data were collected by questionnaire and hypertension control cards. The analysis was done using chi Square.

**Results**

Intervention in 6 health workers resulted 83.3% improvement in counselling skill. It was found that 100 (77%) patients became obedient and 30 (23%) patients did not comply. A total of 76 people had reached blood pressure targets (76%), and 23 were uncontrolled (23%). The causes of non-compliance were lack of public knowledge, lack of counseling, and incorrect salt density.

**Conclusions**

Intervention in health worker (counselling skill), and intervention in patients (education and motivation) could improve blood pressure control compliance.

## O88 Descriptive analysis of health services in Barrang Lompo Island, Makassar City, Indonesia

### Amran Razak^1^, Munawar^2^, Sitih Nur Djanna Renfaan^2^, Ainun Nidaá^2^, Ayu Sri Wiyanti^2^

#### ^1^Department of Health Policy and Administration, School of Public Health Hasanuddin University, Makassar, 27867, Indonesia; ^2^Graduate School of Public Health Hasanuddin University, Makassar, 27867, Indonesia

##### **Correspondence:** Amran Razak (profamranrazak@gmail.com)

**Background**

This study aimed to provide an overview of various aspects of health services in Barrang Lompo Island, Makassar City, Indonesia.

**Materials and methods**

This research used a mixed method by studying factors associated with utilization and satisfaction of health services obtained by of BPJS Kesehatan (Indonesian social security administering body on health) beneficiaries, influence of intrinsic and extrinsic motivation on health performance, analysis of drug management system at community health centers in Barrang Lompo Island.

**Results**

This study illustrated that health care utilization was most influenced by pain complaint (p = 0.004). The quality of health services was much determined by assurance dimension (98%). Extrinsic motivation, especially incentives, and intrinsic motivation such as recognition influenced officer performance significantly. Furthermore, there was still something problem in drug management such expired drugs.

**Conclusions**

This study described that the utilization of health services was influenced by pain complaint. The level of satisfaction of the BPJS Kesehatan patients on the quality was quite high. Recognition has the most influence on the performance of community health center officers. Drug management needs to be strengthened.

## O89 The implementation of drug counseling and VCT (Voluntary, Counseling and Testing) in preventing HIV/AIDS among drug user groups in Biaro Health Center, Agam District, Indonesia

### Abdiana, Rima Semiarty

#### Department of Public Health, Faculty of Medicine, Andalas University, Padang, 25147, Indonesia

##### **Correspondence:** Abdiana (abdiana_epid08@yahoo.com)

**Background**

Biaro Health Center is one of community health centers in Agam district, West Sumatera province, which has started to deal with the problem of drug users and HIV/AIDS since 2007. The programs and activities that have been implemented including prevention, therapy, and rehabilitation programs. The purpose of this study was to get an overview of the implementation for drug and VCT counseling services to prevent HIV/AIDS among drug users in Biaro Health Center in 2014.

**Materials and methods**

This study was a qualitative research. The population in this study were health workers providing drug counseling and VCT services at Biaro Health Center, patients with drug abuse at Biaro Health Center Agam District. The sample collection technique done by in-depth interviews.

**Results**

The study obtained that human resources involved in counseling and VCT activities were sufficient in terms of number and educational background. The facilities and infrastructures used in counseling and VCT services were sufficient. Funds used for counseling and VCT services were sufficient for rapid HIV testing. Counseling and VCT services had been implemented in accordance with VCT service guidelines. Planning of consolidation activities and VCT was integrated with the planning of the Health Center program. There was a clear organizational structure in counseling and VCT activities, so that the task and authority were clear. Stages of VCT services had been implemented from pre-testing consoles to post-testing consoles. Evaluation activities conducted in the form of patient data collection.

**Conclusions**

It is expected to improve VCT training and to improve officer skills in VCT services. Carry out VCT quality control activities to ensure the availability of quality VCT services is also important.

## O90 Potential source of inefficient budget expenses under Indonesian national health insurance (JKN) scheme in financing cardiovascular disease in West Sumatera Province, Indonesia

### Ade Suzana Eka Putri (adesuzana@gmail.com)

#### Department of Epidemiology and Biostatistics, Andalas University, Padang, 21528, Indonesia

**Background**

Cardiovascular diseases absorbed a high portion of Indonesian National Health Insurance (JKN) claims due to its expensive and long-term treatment. This study aimed to identify the potential source of inefficient budget expenses under JKN scheme particularly in financing cardiovascular disease in West Sumatera Province, Indonesia.

**Materials and methods**

This study was conducted in five districts in West Sumatera Province. Mixed method of quantitative and qualitative research was employed. Data were collected from medical records of JKN patients with cardiovascular disease determined by ICD 10 code to identify upcoding occurrence in two secondary hospitals which was selected purposively according to the availability of JKN claim data. Public health staffs in one randomly selected primary health center from each district were interviewed regarding cardiovascular prevention program.

**Results**

Ischemic heart disease was found as the most frequent diagnosis claimed by hospitals.

The proportion was 34.5%, followed by cerebrovascular diseases (26.8%) and hypertension (16.7%). Upcoding was found in 34 out of 183 medical records (18.6%) of patients with cardiovascular diseases with estimated loss of JKN funds was 8.6% of hospital total claims on cardiovascular diagnosis. Furthermore, this study found that hypertension was a priority to be solved and prevented by primary health center. However, health promotion funds were not used properly by primery health centers and the utilization of cardiovascular disease early detection service by the community was very low.

**Conclusions**

The potential source of inefficient budget expenses under JKN scheme particularly in financing cardiovascular disease were money loss due to upcoding practice and low utilization of health promotion and cardiovascular prevention funds by primary health centers.

## O91 Trends and determinants of long term contraceptive method (LTCM) use among married women in East Java

### Ni’mal Baroya^1^, Andrei Ramani^1^, Thohirun^1^, Iswari Hariastuti^2^, Nyigit Wudi Amini^2^

#### ^1^Department of Epidemiology, Biostatistics and Population Studies, Public Health Faculty, University of Jember, Jember, 68111, Indonesia; ^2^National Board of Population and Family Planning East Java, Surabaya, 60286, Indonesia

##### **Correspondence:** Ni’mal Baroya (nbaroya@unej.ac.id)

**Background**

The using effective contraceptive methods become an important strategy for reducing maternal mortality through prevention of unwanted pregnancies. However, the using of long term contraceptive method (LTCM) in East Java is still low. This study aimed to assess the trends and determinants of using LTCM among married women in East Java Indonesia.

**Materials and methods**

A cross sectional studies was conducted among married women who become respondent of National Medium-Term Development Plan Survey in 2009-2014. Independent variables were age, education, occupation, welfare status, place of residence, the Human Development Index (HDI), the ratio of field staff of family planning, the ratio of midwives, community health center ratio and ratio of doctors. Data were analyzed using chi square test, correlation test and logistic regression test at the significance level of p value was less than 0.05.

**Results**

Trend of LTCM in East Java looked fluctuated and tend to decrease. LTCM use differed significantly according to age, education, employment status, employment, place of residence and family welfare status. HDI and the ratio of physicians correlated positively, whereas negative correlation was found with field staff of family planning ratio. The ratio of midwives and health centers were not significantly correlated to the use of LTCM in East Java.

**Conclusions**

The determinant of long-term use of contraceptive methods in East Java were age over 30 years, medium and high educated and living in urban areas.

## O92 Accessibility to the acceptance of health services on island community, Indonesia: a qualitative study

### Sukri Palutturi^1^, Muliana^2^, Darmawansyah^1^, Amran Razak^1^, Sitti Haerani^3^, Masni^4^, Hamzah^5^

#### ^1^Department of Health Policy and Administration, Faculty of Public Health, Hasanuddin University, Makassar, 90245, Indonesia; ^2^Graduate School of Public Health, Department of Health Policy and Administration, Faculty of Public Health, Hasanuddin University, Makassar, 90245, Indonesia; ^3^Department of Management, Faculty of Economics, Hasanuddin University, Makassar,90245, Indonesia; ^4^Department of Biostatistics and Population and Family Planning, Faculty of Public Health, Hasanuddin University, Makassar, 90245, Indonesia; ^5^Faculty of Law, Hasanuddin University, Makassar, 90245, Indonesia

##### **Correspondence:** Sukri Palutturi (sukritanatoa72@gmail.com)

**Background**

The accessibility of health services to island communities has been widely examined quantitatively, including Indonesia. A further assessment for a more comprehensive decision-making process is indispensable. This study aimed to explore the health services of island communities Satangnga, Indonesia.

**Materials and methods**

This research was a qualitative research with case study approach. In-depth interview was conducted to the 19 informants: village secretaries, village staff, head of Public Health Center (PHC), and head of Auxiliary PHC, midwives, community leaders, and pregnant women who had used health services. Informant selection was done purposively.

**Results**

The results showed that the accessibility of public health service of Satangnga Island has not been fulfilled. This was due to the unavailability of health equipment. The community responds positively to public health services without discrimination although the availability of facilities and infrastructure at health facilities still inadequate and needs to be improved.

**Conclusions**

The community has accessed health facilities, but the service is only in auxiliary PHC. From the cultural side, people give positive response because there is partnership between midwife and shaman especially in delivery service. Additional health personnel such as doctors is needed.

## O93 Implementation of the operational cooperation in the agency’s hospital public service areas of Surabaya with private parties 2016

### Christyana Sandra (christyana_sandra@yahoo.com)

#### Department of Administration and Health Policy, Faculty of Public Heatlh, University of Jember, Jember, 68121, East of Java, Indonesia

**Background**

Regional general hospitals are unit of work most local government changed its status into BLUD. The improvement of the status of the provincial hospitals being BLUD means as a form of professional public service by the government of the region. Nevertheless, some people criticized this policy because it basically indicates that the actual local government hasn not been able to manage the abundant funds and empowers them to provide quality public services. The objectives of this research was to describes the conditions of implementation regional BLUD hospital operational cooperation refers to the legislation.

**Materials and methods**

This was a descriptive research conducted at the 3 BLUD Hospitals in Surabaya in April until October 2016.

**Results**

Operational cooperation is as a focus the issues to be examined from the perspective of legal consistency in applying the BLUD General Hospital area in a running partnership with another party. The government's actions in commercial transactions put the government in the scope of private law. The paper agreement or cooperation conducted by the local government poured in the form of a cooperation agreement between the government of the region can be based on private law or public law. The implementation of the provincial hospitals in cooperation with private parties must meet to be able to improve the quality of service of hospitals and can increase the income of the provincial hospitals. Cooperation between the provincial hospitals with a third party, among others, could be operational co-operation, renting, and other businesses that support the tasks and functions of the provincial hospitals.

**Conclusions**

Until the end of this study there is no legislation which governs specifically about the mechanism of the selection of potential partners to implement operational co-operation for BLU.

## O94 Factors associated with chronic energy deficiency among pregnant women in Padang, Indonesia

### Idral Purnakarya (idral_pkarya@yahoo.com)

#### Faculty of Public Health, Andalas University, Padang, 25172, Indonesia

**Background**

The problem of chronic energy deficiency (CED) in pregnant women is one of the most of maternal health and nutrition problems in Indonesia. This study aimed to identify factors associated with CED among pregnant women in Padang.

**Materials and methods**

The study design was cross-sectional. Samples were pregnant women with 16-32 weeks of gestational age in Padang with the total of 171 pregnant women. The characteristics of subjects included the age of pregnant women, gestational age, education level and expenditure level. Data of nutrient intake consist of energy intake, protein intake and Fe intake obtained from Semi-quantitative food frequency questionnaire (SFFQ). CED was assessed by upper arm circumference (MUAC).

**Results**

Result showed that prevalence of CED among pregnant women was 9.9 percent. The median of upper arm circumference (MUAC) of pregnant women was 27.5 cm (21.5 to 39.0 cm). The results showed that Fe intake was significantly associated with CED among pregnant women (p <0.05). However, energy intake, protein intake and other factors were not significantly associated (p> 0.05).

**Conclusions**

Hence, a serious effort was need to meet adequate levels of food consumption of pregnant women doing by promote on the selection of foods with contain balanced nutrition.

## O95 The effectiveness of tax and price policy for tobacco control in Southeast Asia: a systematic review

### Wardiah Rizalia^1^, Anggraini Sevrima^2^

#### ^1^Department of Administration and Health Policy, Faculty of Public Health, Universitas Indonesia, Depok, 16424, Indonesia; ^2^Department of Biostatistics, Faculty of Public Health, Universitas Indonesia, Depok, 16424, Indonesia

##### **Correspondence:** Wardiah Rizalia (rizaliawardiah@gmail.com)

**Background**

Tobacco taxes and prices are considered as the most cost-effective means to reduce consumption and address the burden of morbidity and mortality. This study was a systematic review that focuses on taxes, prices, and tobacco control.

**Materials and methods**

Researchers traced multiple databases from ProQuest and Scopus and examined several studies that fit the inclusion and exclusion criteria. Researchers conducted a systematic review of 8 studies, all of them were cohort studies.

**Results**

Based on the results of systematic review it was known that the price of cigarettes in Malaysia was MYR 9.40 became MYR 9.55, a significant increase (p=0.006) and led to Malaysian cigarette consumption. In 1999, cigarette taxes in Thailand were raised to 71.5%, as smoking had decreased and was not socially acceptable. In Vietnam, the prevalence of smoking in men exceeded 50%, but students accelerated the onset of smoking by 14.8% compared to people who were no longer in school or at work. Each country had price gap. In Vietnam, 76% of international brands and 86% of domestic brands for sale at VND 1000. In the Philippines, 57% of single international brand was sold for PHP 2, and 86% of single brand domestic brand was sold for PHP 1.

**Conclusions**

Based on the results of 8 studies on taxes, prices and tobacco control, it is known that every country in southeast Asia has the same policy of raising tobacco tax and cigarette price for tobacco control and cigarette consumption.

## O96 Thailand experience in hospitalization policy for new sputum smear positive pulmonary tuberculosis patients

### Juthapat Rattanadilok Na Bhuket^1^, Defriman Djafri^2^, Petchawan Pungrassami^3^, Virasakdi Chongsuvivatwong^4^

#### ^1^Bureau of AIDS TB and STI, Department of Disease Control, Nonthaburi, 11000, Thailand; ^2^Faculty of Public Health, Andalas University, Padang, 25128, Indonesia; ^3^Department of Disease Control, Nonthaburi, 11000, Thailand; ^4^Epidemiology Unit, Faculty of Medicine, Prince of Songkla University, Songkla, 90000, Thailand

##### **Correspondence:** Juthapat Rattanadilok Na Bhuket (juthapat47@yahoo.com)

**Background**

In 2008, Thailand was among the 22 highest tuberculosis burden countries. In 2009, the Ministry of Public Health announced new policy to hospitalize for NSS+ PTB patients for two weeks. In 2010, this study objective was to evaluate treatment outcomes under a 2-week admission policy for NSS+ PTB patients.

**Materials and methods**

The registry patients during October 2008 – September 2009 was retrieved from 59 public hospitals. The patient records were reviewed and categorized into 6 groups: (a) non-admitted, (b)admitted before diagnosis, (c)admitted within 1 week of diagnosis and period (c1) < 2 weeks, (c2)2 weeks, (c3)>2 weeks, and (d)delayed admission after diagnosis. Main outcomes were treatment success, death and default.

**Results**

Of the 2809 eligible patients. The respective rates of success/death/default among the non-compliant (a+d), partially compliant (c1) and fully compliant (c2+c3) groups were 87.1%/4.9%/3.4%, 85.0%/10.2%/1.0%, and 83.3%/10.6%/2.0%, respectively.

**Conclusions**

Admission of NSS+ PTB patients under the study was far from the policy statement. Relationship between admission and treatment outcome was probably confounded by the severity of the patients.

## O97 Law enforcement on the implementation exclusive breastfeeding among independent practice midwives in Maros, Indonesia in 2016

### Indar^1^, Nurhayani^1^, Arsyad^2^

#### ^1^Department of Health Policy and Administration, Faculty of Public Health, Hasanuddin University, Makassar, 90245, Indonesia; ^2^Departmentt of Health, Biak, Indonesia

##### **Correspondence:** Indar (indar.sh@gmail.com)

**Background**

This research aimed to investigate the responsibility of the government, the independent practice midwives (BPM), and the mothers for the provision of exclusive breastfeeding for the newborn babies.

**Materials and methods**

The study was a quantitative research. The samples were 20 BPM and 20 mothers who gave birth in respective BPM. Samples were chosen by using cluster sampling technique.

**Results**

The research results revealed that the breastfeeding had not run well due to the facts that 14 BPMs (70 %) still sold infant formula milk, 13 BPMs (65 %) did not implement breastfeeding initiation, and 15 BPMs (75%) did not provide information about the benefits of breastfeeding. It was found that only 2 mothers (10 %) did not exclusively breastfeed their babies due to medical indication reasons, and 18 mothers (90 %) did not breastfeed their babies due to their inadequate breats milk which made their babies cried. The research showed that from the 20 mothers who gave birth in BPM, only 3 mothers (15%) knew that breastfeeding was their own responsibility as regulated in the Health Regulation, while 17 mothers (85%) did not know the existence of such regulation.

**Conclusions**

The research showed that the central and regional governments had set up regulations reminding that the newly born babies had the legal right to receive breast milk and therefore it was the obligation of all mothers to breastfeed the newly born babies exclusively. However, the implementation and the control of the matter had not been complied because of some of the BPM did not obey the regulation and some of the customers were not sanctioned.

## O98 Vaccines, GAVI-Alliance and Civil Society Organizations (CSOs) in developing countries: prospect and challenges

### Sharifa Ezat Bt Wan Puteh^1^, Umar Ibrahim^2^

#### ^1^Head, International Centre for Case-mix and Clinical Coding (ITTC), UKM Medical Centre, Kuala Lumpur, 56000, Malaysia; ^2^International Institute for Global Health (UNU-IIGH), Kuala Lumpur, 56000, Malaysia

##### **Correspondence:** Sharifa Ezat Bt Wan Puteh (shaezat@gmail.com)

**Background**

Immunization is a practical preventive health mechanism that protect lives in line with SDG 3 stipulations. A feat made possible by GAVI Alliance (Global Alliance for Vaccines and Immunizations), which save millions lives through its work in developing countries. Against this background, the article assessed, ‘Vaccines, GAVI Alliance and CSOs roles in developing countries; prospects and challenges.

**Materials and methods**

The study employed desk survey review approach by exploring published and unpublished materials on GAVI-Alliance, immunizations and roles of CSOs in GAVI’s constituency in developing countries

**Results**

GAVI Alliance had successfully through it supported vaccines approach immunized over 500 million children, averted about 7 million deaths. GAVI’s contribution and SDG 3 aspirations of reducing morbidity and mortality rates among children under the age of five by 2030 is a realizable ambition, but the project is not without challenges, such as insecurity and inadequate financing among others.

**Conclusions**

Reducing the burden of morbidity and mortality rates in developing countries, as outlined in SDG 3 targets, is vaccines and partnership dependent. Indeed, without partnership among governance sectors, as demonstrated by GAVI Alliance, SDG 3 targets cannot be met by 2030.

## O99 Development of *nagari* institutional involvement in West Sumatra as a strategic effort to increase BPJS membership to achieve universal health coverage in 2019

### Denas Symond, Nizwardi Azkha, Hafifatul Auliya Rahmy

#### Faculty of Public Health of Andalas University, Andalas University, Padang, 25172, Indonesia

##### Denas Symond (denaspdg@gmail.com)

**Background**

Universal Health Coverage is a health system that ensures every citizen in the population has fair access to high quality health services at affordable cost. The objective of the research was to know the local institutional diversity in rural area, and to arrange alternative institutional model to increase the participation of Indonesian national health insurance (JKN).

**Materials and methods**

This research was conducted in two villages in Padang Pariaman District in July to November 2016. The method was a combination of survey and FGD.

**Results**

This study found that 67% of respondents did not registered as participants of JKN due to the complexity of administrative system. Existing local institutions and groups had not played an optimal role in improving JKN participation. Establishment model of institutional group empowerment in two villages, that are *badoncek saparuik kaum* and *arisan korong* or *nagari*.

**Conclusions**

This model demonstrated the strategic role of institutions and local groups in increasing the participation under JKN.

## O100 The influence of nutrition education through EMODEMO to the behavior of food management and food assembling in pregnant woman in Padang City, Indonesia

### Denas Symond^1^, Erwinda^2^

#### ^1^Faculty of Public Health, Andalas University, Padang, 25172, Indonesia; ^2^Faculty of Medicine, Andalas University, Padang, 25172, Indonesia

##### **Correspondence:** Denas Symond (denaspdg@gmail.com)

**Background**

Nutritional education for pregnant women is important to reduce the risk of anemia. A method for improving the nutritional knowledge and attitude among pregnant women is the using of media such as EMODEMO*.* EMODEMO was developed as health education media the behavior of food management and food intake in pregnant women.

**Materials and methods**

This type of this research was an experimental research with pre- and post-test approach. This study was carried out in Padang since March until May 2017. The study population was pregnant woman with a total of 38 pregnant women. Sampling was done by purposive sampling method. Nutrition education using EMODEMO media was conducted.

**Results**

There was a significant difference in the behavior of pregnant women after receiving nutritional education at p value < 0.001. The nutritional intake of pregnant women increased after being given nutrition education for energy content, protein, fat and carbohydrate. It was found that 72% of pregnant women's knowledge increased regarding nutritional needs, iron and nutritious food sources as an effort to save the first 1000 days of life.

**Conclusions**

This study showed that nutrition education with EMODEMO could be used to improve the knowledge and attitudes of pregnant women on food management and food intake the city of Padang.

## O101 Analysis of smoking habit on periodontal status in Solok City, West Sumatera, Indonesia

### Minarni^1^, Susi^2^

#### ^1^Department of Dental Nursing, Kemenkes Health Polytechnic, Padang, 25146, Indonesia; ^2^Faculty of Dentistry, Andalas University, Padang, 25172, Indonesia

##### **Correspondence:** Minarni (nenny8869@yahoo.co.id)

**Background**

Cigarettes contain harmful toxin that interfere human’s health. Smoking habits not only cause systemic effects, but also cause pathological conditions in oral cavity. One of them is gingival changes and destruction of periodontal tissue in various severity. Cigarette consumption in West Sumatera continues to increase despite adverse health effects. This research aimed to determine the effect of smoking habit on severity of periodontal status in Solok, West Sumatera, Indonesia.

**Materials and methods**

The research design was analytic descriptive with cross sectional approach. Samples were taken by simple random sampling method with total sample of 124 respondents. Inclusion criteria were male smokers aged 25-45, and smoking filter type cigarrete. Exclusion criteria were those who had systemic disease and using artificial teeth. Data collection was using questionnaires and Periodontal Disease Index checking using Ramjord teeth. Data were analyzed by using chi square test with confidence interval of 95 %.

**Results**

The study found that 6.5% of respondents were light smokers, 79% were moderate smokers, and 14.5% of respondents were heavy smokers. Healthy periodontal tissue was found in 2.4% of respondents, gingivitis was found in 98 respondents (79%) and periodontitis was found in 23 respondents (18.6%). The study revealed that smoking was significantly related to periodontal tissue condition.

**Conclusions**

Smoking habit influences severity of periodontal status which moderate smokers had gingivitis dominantly in Solok, West Sumatera.

## O102 Local wisdom and antenatal care behaviors among Kajang ethnic in Bulukumba, Indonesia

### Nurdiyanah Syarifuddin^1^, Dwi Santy Damayati^2^, Rizky Chaeraty Syam^1^, Muhammad Rachmat^3^

#### ^1^Department of Health Promotion and Behavioral Science, Faculty of Medical & Health Sciences, Universitas Islam Negeri Alauddin Makassar, Sulawesi Selatan, 92111, Indonesia; ^2^Department of Nutritional Science, Faculty of Medical & Health Sciences, Universitas Islam Negeri Alauddin Makassar, Sulawesi Selatan, 92111, Indonesia; ^3^Department of Health Promotion and Behavioral Science, Universitas Hasanuddin Makassar, Sulawesi Selatan, 90245, Indonesia

##### **Correspondence:** Nurdiyanah Syarifuddin (nurdiyanah@uin-alauddin.ac.id)

**Background**

Kajang Society is one of many ethnic groups in South Sulawesi, located in the district of Bulukumba. The people is still strongly hold their traditions and their daily behavior always in harmony with nature. This study aimed to describe local values and the behavior of antenatal care among Kajang Society in Bulukumba District, South Sulawesi.

**Materials and methods**

In this ethnographical qualitative research, in-depth personal interviews and observation were conducted in pregnant mothers, family members, and traditional shaman, midwifery and community leaders in Kajang ethnic group. Data collection continued until saturation stage.

**Results**

The results showed that local wisdom determined with pre- and post-natal care in Kajang Society and until this day, the local values still remained used. Pre-natal care in Kajang’s tradition was a form of a series of rituals which conducted since seven months of pregnancy until 40 days after delivery. Local knowledge, believes and tradition, and family support became determinant factors in forming a community adherence in Kajang ethnic.

**Conclusions**

It is important to identify the values that are still available in the community as an asset to develop healthier behaviors and as a basis for designing culture-based health promotion programs in the society.

## O103 Factors affecting utilization of antenatal care services in Sana'a City, Yemen

### Seham Othman^1,2^, Taha Almahbashi^2^, Alabed Ali A Alabed^3^

#### ^1^Community medicine Department, Faculty of Medicine, Sana’a University, Sana’a, Yemen; ^2^High Institute of Health Sciences (HIHS), Sana’a, Yemen; ^3^Department of Community Medicine, Faculty of Medicine, Lincoln University College (LUC), Selangor Darul Ehsan, Malaysia

##### **Correspondence:** Alabed Ali A Alabed (abed11k@gmail.com)

**Background**

Antenatal care (ANC) is a vital part of primary healthcare that is known to improve maternal and newborn outcomes. The aim of this study was to identify the factors affecting utilization of ANC services for women in reproductive age in Yemen.

**Materials and methods**

This cross-sectional community-based study was conducted in six districts of Sana’a City in Yemen. Data were collected from 460 mothers who gave birth in the past six months via face-to-face interviews at home between September to December 2010.

**Results**

Only 54% of mothers were found to have four or more ANC visits. Almost two third of participants made their first visit during their first trimester due to presence of health problems and did not follow up when they became healthy during pregnancy. Reasons for not receiving ANC services were an absence of health problems, high cost of ANC services, long waiting time, and poor staff attitude. Sixty percent of participants were unaware of the danger symptoms of common health problems in pregnancy. The significant factors affecting utilization of ANC services were mother education, residence place, age at first pregnancy, gravida, parity, occurrence of pregnancy without planning, and number of live children.

**Conclusions**

The factors affecting the number of visits were mother education, place of residence, and husband work. Future healthcare activities should focus on improving women’s awareness of the importance of ANC even in the absence of noticeable health problems and lack of education about the common danger signs and symptoms of pregnancy.

## O104 The need of public health professionalism in accelerating SDGc achievement

### Hanifa Maher Denny (hanifadenny@live.undip.ac.id)

#### Faculty of Public Health, Diponegoro University, Semarang, Indonesia

**Background**

The agenda for sustainable development goals rely on each country policy and program and global partnership by multi stakeholders.

**Materials and methods**

This paper review utilized secondary data from the Ministry of Health website, other means of official websites related to SDGs in health aspects, non-published document from the management of Faculty of Public Health Diponegoro University and the data that were cited from the Indonesian workshops/conferences related to public health developments in Indonesia as well as the peer review journals.

**Results**

Public health professional faces some challenges due to multi complex problems and situations in health. Constructing public health student by providing a professional continuing education is an alternative to improve the competence of public health practitioners. Moreover, involving public health students to conduct field practices on the existing community health problem is an effective method to expose them with some applicable solution.

**Conclusions**

The competence and performance of public health professional would be a big contributor asset for the achievement of SDGs in health.

## O105 Tuberculosis in Cinangka Serang Banten, Indonesia: a social and cultural perspective study of disease

### M. Farid (fr_sayoon@yahoo.co.id)

#### PSKM Fakultas Kedokteran dan Imu Kesehatan, UIN Syarif Hidayatullah, Jakarta, 15412, Indonesia

**Background**

The reality of tuberculosis in Indonesia has never been addressed since colonial era. The health scientist often disregards the results of social and cultural perspective research on tuberculosis. Etiology and transmission of disease is not only biological, but social conditions and culture too. Disease is not found, but constructed and resulted by social process. This study aimed to offer “the power” as a new theme of social and cultural perspective on tuberculosis.

**Materials and methods**

Participation observation and in-depth study conducted to personality and naturalistic shamans or indigenous medical practitioners, staffs of public health center, and tuberculosis suspects or sufferers and their family. The data also collected from local public figures and relevant community members.

**Results**

The results of this research were that the knowledge of tuberculosis was constructed by the community of Cinangka Serang Banten, and the implementation of power can stimulate, push, or even facilitate the biological process of tuberculosis.

**Conclusions**

The social process (power) and biological process are an integrated process of tuberculosis, and cannot be viewed separately. For tackling and preventing tuberculosis must integrate the social and biological process.

## O106 Dole-Dole tradition in health seeking behavior for Butonese community, Southeast Sulawesi, Indonesia

### Andi Asrina^1^, Sukri Palutturi^2^, Andi Tenri^3^

#### ^1^School of Public Health, Muslim University of Indonesia, Makassar, 90231, Indonesia; ^2^School of Public Health, Hasanuddin University, Makassar, 90245, Indonesia; ^3^Dayanu Ikhsanuddin University, Baubau, 93711, Indonesia

##### **Correspondence:** Andi Asrina (rinatibrisi@yahoo.com)

**Background**

Perceptions about the causes and cures of diseases vary greatly among different community groups in Indonesia including in Buton. This study aimed to examine the Butonese community health seeking pattern for infants and under five years based on personalistic etiology.

**Materials and methods**

This type of research was qualitative using an ethnographic approach. The study was conducted at Baubau. Eight informants were interviewed; 4 ordinary informants, 2 key informants and 2 persons of supporting informants. Data collection methods were observation, in-depth interviews and documentation. Data were analyzed in cultural domain.

**Results**

The results showed that the treatment seeking started from home treatment to traditional health and modern treatment. Dole-dole tradition performed by a shaman was believed by local community and practiced hereditary as a powerful way of prevention, immunity, and healing, especially for infants and children under five years.

**Conclusions**

This study concludes the community health seeking is based on beliefs in traditional healing performed by hereditary and is considered more successful than medical treatment. Acculturation of local culture and modern health services need to be strengthened.

## O107 Characteristics of host and house environment of leprosy patients

### Juwita Wijayanti, Ela Laelasari, Yuli Amran

#### Public Health Department, Islamic State University Syarif Hidayatullah, Jakarta, 15412, Indonesia

##### **Correspondence:** Yuli Amran (yuli.amran@uinjkt.ac.id)

**Background**

Indonesia has achieved elimination of leprosy in 2000. However, in South Tangerang City, there are areas with leprosy prevalence rate more than 1 per 10.000 population. This disease causes social, economic and psychological problems. Therefore, this study aimed to analyze the characteristics of the host and house environment in order to formulate the intervention prevention and mitigation plan.

**Materials and methods**

This research was a descriptive quantitative study with case series design. The samples of this research were 34 leprosy patients in South Tangerang. Data were collected by distributing questionnaires to patients, measuring and observing house environment conditions. Descriptive data analysis was conducted.

**Results**

The results showed that most (88.2%) of leprosy patients were at productive age, 67.4% were male, 76.5% were poorly educated, 55.9% had low-income, 50% worked as laborers or farmers, 55.9% had poor bathing habits, and over 60% had the habit of lending clothes or towels to others. More than half of patients’ houses were at risk for developing leprosy disease.

**Conclusions**

Based on the results of this study, it is advisable in patients to treat multidrug therapy, changing healthy behavior and maintaining the healthy house environment.

## O108 Factors related with quality of life of type 2 diabetes mellitus patients in Dr. M. Djamil Hospital, Padang City, Indonesia

### Fauziah Elytha, Vivi Triana, Yoni Fitri Aprilla, Roma Yuliana

#### Epidemiology and Biostatistics Department, Faculty of Public Health, Andalas University, Padang, 25148, Indonesia

##### **Correspondence:** Fauziah Elytha (elytha12@gmail.com)

**Background**

Type 2 diabetes mellitus patients have much lower quality of life (71.2%). This study aimed to identify factors related to quality of life of type 2 diabetes mellitus patients at Dr. M. Djamil Hospital in Padang.

**Materials and methods**

This study was quantitative research with cross sectional design. The population was patients who were diagnosed with type 2 diabetes mellitus treated at Dr. M. Damil Hospital in 2016. This study involved 57 respondents as samples. The sampling technique was simple random sampling. Data was collected through interview by using questionnaire and analyzed by using bivariate and multivariate analysis.

**Results**

The study found that 68.4% respondents had low quality of life. There was significant relationship between diabetes complications, family support and depression level with quality of life of type 2 diabetes mellitus patients. Multivariate modeling showed that the dominant factor related to quality of life was depression level.

**Conclusions**

Diabetes complications, family support and depression level influenced the quality of life of type 2 diabetes mellitus patients at Dr. M. Djamil Hospital in Padang. This study suggests to the health professionals to improve the implementation four pillars of diabetes mellitus management and involve the family in counseling.

## O109 Android based mobile phone application to support student to quit smoking in Andalas University

### Ayulia Fardila Sari ZA (ayuliafardila@gmail.com)

#### Public Health Faculty, Andalas University, Padang, 25163, Indonesia

**Background**

Andalas University has implemented no smoking policy for all academic community, but it has not followed by the decreasing number of students who smoke. Based on initial study, 85% of engineering faculty’s students and 75% of law faculty’s students are smokers. It requires innovative effort to control the number of students who smoke in Andalas University. The goal of this research was to develop Android base mobile phone application to support students to quit smoking in Andalas University.

**Materials and methods**

This research was an action research by using qualitative method to explore step by step collaboration in developing Android Base Supporting Mobile Phone Application to quit smoking. The research was conducted in Andalas University. Data collection method used was Focus Group Discussion (FGD) on two groups of students from Engineering Faculty and Faculty of Law and by using in-depth interview with health promotion lecturer.

**Results**

Based on the result of the research, the informant prefered android-base application which informative and consists entertainment. All informants agreed with application with calculation on economic and health impact when they smoke or quit smoking. Most of them liked the view of standard, simple, lightweight application and the existence of daily pops up to remind them to quit smoking. Most of informant wanted counseling content with experts, and most of them did not want application that was connected to social media.

**Conclusions**

Supporting application to quit smoking named “Quit Smoking” and can be used by users of android mobile phone. The content meets user’s need but no counseling content. The evaluation of the benefit of this application must be done for improvements.

## O110 Opportunities, challenges and benefits of big data analysis in healthcare for decision making on disease control and prevention (a review)

### Marko Ferdian Salim (markoferdiansalim@mail.ugm.ac.id)

#### Department of Health Information and Services, Vocational College, Universitas Gadjah Mada, Yogyakarta, 55281, Indonesia

**Background**

Growth and development of data more rapidly in line with the development of information technology that also provides a positive impact on health sector. Medical data not only in large number, but also complex, varied, and actual. Thus, opportunities for the implementation of big data analysis on health care facilities is very potential. However, it has not been optimal in many developing countries compared to developed countries.

**Materials and methods**

This paper was a review with narrative review category. Source of material was from relevant journal and textbook on big data analysis. This paper describes the opportunities, challenges and benefits of using big data analysis in health services.

**Results**

Opportunities for the implementation of big data analysis in healthcare facilities are very potential and have positive impacts, such as the use of electronic health record (EHR). The Challenges are data structure, data security, data standardization, data quality, storage and transfers of data, managerial issues, lack of skill human resources, regulatory compliance, and real-time analytics. Benefit of big data analysis can be used in decision making to disease prevention and control, prediction, and identification of disease risk factors in healthcare.

**Conclusions**

Utilization of big data analytics for health services have positive impact although there are still challenges that need to overcome.

## O111 Applying the technology acceptance model in primary health centers

### Angga Eko Pramono, Dian Budi Santoso

#### Vocational College, Gadjah Mada University, Yogyakarta, 55281, Indonesia

##### **Correspondence:** Angga Eko Pramono (anggaekopramono@ugm.ac.id)

**Background**

Primary health centers in Yogyakarta have implemented health information systems since 2005. The following years, other health information systems started to be implemented up to seven systems. This study aimed to examine the key factors that could influence the healthcare professionals in primary health centers on health information system use.

**Materials and methods**

A questionnaire survey was conducted from October to November 2015 at primary health centers in Yogyakarta. A hundred and seventy questionnaires were distributed among healthcare professionals. The analysis was performed to test the validity and reliability of measurement models and hypotheses proposed using SEM-PLS and technology acceptance model (TAM).

**Results**

A response rate of 58,8% was achieved. Convergent and discriminant validity values and also cronbach alpha values were acceptably high (> 0.7). The TAM concept was good to measure the usage intention of health information system at primary health centers. It was indicated by the significant correlation between variables. Furthermore, correlation between perceived ease of use and perceived usefulness has the most significant value (p= 0.001).

**Conclusions**

The most important factor to be considered by health information system developers and stakeholder is perceived ease of use. It means the system should be easy to learn and operate by the healthcare professionals. Thus, it will enhance their intention and encourage them to use the system properly.

## O112 The effectiveness of self-administered and interview based questionnaire method in the reporting sexual behavior among the youth in Medan City, Indonesia

### Sri Rahayu Sanusi, Lanova Dwi Arde

#### Faculty of Public Health, University of Sumatera Utara, Medan, 20155, Indonesia

##### **Correspondence:** Sri Rahayu Sanusi (ayusans@yahoo.com)

**Background**

The result of population census showed that about 41 million or 26% of the Indonesian population are adolescent aged 10-24 years. This large number of young people will cause many problems such as sexual behaviors. Sexual health problem is a sensitive issue that is both privacy and at the same time regulated by social and religious norms. This is why getting true number of youth sexual behavior is difficult. This study aimed to compare the effectiveness of self-administered and interview based questioner method to obtain a true number of youth sexual behavior.

**Materials and methods**

It was a descriptive-quantitative study. The samples of this study were 366 students of senior high school and a college in Medan City aged 15-21 years, selected by simple random sampling.

**Results**

This study showed that using self administered method, 7.9% of respondents reported that they got intercourse when they were with boy or girlfriend. By using interview-based questioner method, 5% of respondents reported that they ever got intercourse.

**Conclusions**

The result showed that there was difference in reporting youth sexual behavior between self administered method and interview-based questioner method. Young people are more likely to report sexual behaviors by self administered method compared to interview-based questioner method, especially behavior that is still considered taboo such as intercourse.

## O113 Designing the next generation of national electronic health record for primary health care in Indonesia

### Dian Budi Santoso, Angga Eko Pramono

#### Vocational College, Gadjah Mada University, Yogyakarta, 55281, Indonesia

##### **Correspondence:** Dian Budi Santoso (dianbudisantoso@ugm.ac.id)

**Background**

As a developing country, Indonesia made a quantum leap in the utilization of information technology with developing the first national electronic health record (EHR) for primary health care to support the national health insurance program. This study objectives were to identify the user needs and design the next generation of EHR in primary health care.

**Materials and methods**

Participatory action research used to design the new EHR in collaboration with 25 primary health care in Yogyakarta, Indonesia.

**Results**

Indonesia has an infrastructure to connect and integrate all primary health care patient data but limited to national health insurance member which not all citizens become beneficiaries. As for medical record content, it needs more additional data items to make it more comprehensive in recording patient care history. The user experience, data standardization, security system, information exchange, and patient referral system also become important issues in the process of designing the new EHR.

**Conclusions**

The next generation of national EHR for primary health care need to be designed based on user needs assessment to take the next step of development process.

## O114 Sustainability analysis of non-communicable diseases prevention model in Benda Village, Sukabumi Regency

### Sri Handayani (aniverret@gmail.com)

#### National Institute of Health Research and Development, Ministry of Health of Indonesia, Jakarta, 10560, Indonesia

**Background**

In 2015, National Health Research and Development, Indonesian Ministry of Health implemented NCD’s community based prevention model in 8 Indonesia regency. The objective of this model was to control NCD’s such as hypertension, stroke, diabetes, coronary heart disease through community empowerment.

**Materials and methods**

A qualitative research design was conducted in Benda village, Sukabumi Regency. The methods for data collection were in-depth interview and observation. The informants of this study were 16 people in Benda village who were trained as NCD’s agents of change. Agents of change were expected to encourage people to do a healthy life styles such as not smoking, eating healthy food and increasing physical activity.

**Results**

Agents of change couldn’t perform their role optimally. They still faced difficulty to practice healthy life styles, particularly to change their smoking behavior. Lack of technical assistance from supporting facilitator also hampered the succesfull of the program. In addition, agents of change have not utilized their social networks optimally to support the sustainability of this program.

**Conclusions**

Sustained non-communicable diseases community based prevention model in Benda village did not reach the targeted result. Local government support is needed for program sustainability.

## O115 SWOT analysis of primary healthcare readiness in the implementation of *e-puskesmas* in Padang City in 2016

### Ayulia Fardila Sari ZA, Nengsih Purnama Sari

#### Departement of Public Health, Andalas University, Padang City, West Sumatera, 25148, Indonesia

##### **Correspondence:** Ayulia Fardila Sari ZA (ayuliafardila@gmail.com)

**Background**

Padang District Health Office takes part in taking benefit of the progress of technology and information toward the implementation of *e-Puskesmas*. Padang DHO targeted all primary health care implemented *e-Puskesmas* in 2016. The aim of this research was to analyze the readiness of primary health care in implementing *e-Puskesmas* through SWOT method.

**Materials and methods**

This research used qualitative method. Data were collected by using in-depth interview, focus group discussion, observation and document study. The informants were selected by using purposive sampling method. The data were analyzed using SWOT method.

**Results**

This study found that the quality of human resources was still low. Organization readiness was reflected from the positive response of leaders and officers but there was no written policy. Hardware readiness was constrained due to internet and applications that sometimes errors which interfere the quality of service. Based on SWOT analysis, it was known that the preparedness of primary health care was in third quadrant.

**Conclusions**

Primary health care’s readiness was weak but still had a good opportunity. Thus, the priority strategy that need to be done by primary health care is weakness-opportunity strategy such as officer training, the making of policies related to *e-Puskesmas* and the improvement in internet network speed.

## O116 Patient safety climate survey in 11 Indonesian hospitals

### Emma Rachmawati, Ony Linda, Ipik M. Fikri

#### Faculty of Health Sciences, UHAMKA, DKI Jakarta, 13830, Indonesia

##### **Correspondence:** Emma Rachmawati (emma_rachmawati@uhamka.ac.id)

**Background**

The measurement of Patient Safety Climate (PSC) is very important before implementing patient safety initiatives in hospital. The PSC Survey was developed to evaluate the patient safety climate of Indonesian hospital.

**Materials and methods**

A cross-sectional survey design was used. The survey tool was designed to measure 4 dimensions of PSC, including transformational leadership, teamwork, individual consciousness, and PS culture. The survey included 9 personnel characteristics questions and 80 items of safety issues using 5-Likert scales. The instrument was tested to evaluate its psychometric properties and administered to 11 various type of hospitals in Jakarta, Indonesia.

**Results**

The response rate was 68,2%. The psychometric properties of PSC were good (Cronbach’s α: 0.51 to 0.86, t-scores >1.96, and Q square pred. relevance= 68. 9%).T he results showed that there were various scores of 4 dimensions of the PSC. Transformational leadership contributed the highest probability in building better patient safety at hospital level compared to the other dimensions.

**Conclusions**

The PSC survey showed good psychometric properties and was able to measure an accurate assessment of the overall patient safety climate across various Indonesian hospitals.

## O117 Facts and expectations of antenatal care data management quality by the midwives

### Cahya Tri Purnami^1^, Farid Agushybana^1^, Aris Puji Widodo^2^

#### ^1^Department of Biostatistics dan Demograhpy, Diponegoro University, Semarang, 50275, Indonesia; ^2^Faculty of Science and Methematics, Diponegoro University, Semarang, 50275, Indonesia

##### **Correspondence:** Farid Agushybana (hybana@gmail.com)

**Background**

Monitoring health condition of pregnant women by midwives might have an impact on the accuracy of the determination of the risk of pregnancy and the kind of intervention that should be given to a pregnant woman. Accordingly, the accuracy of recording and reporting is important for pregnant women themselves and for midwives. The purpose of this study was to describe and analyze the quality of data management in pregnancy test results conducted by village midwives.

**Materials and methods**

This study used survey method and focus group discussions. The data was processed by descriptive and qualitative approach. The study involved 218 midwives who provide pregnancy services in health centers in 3 districts of Central Java Province.

**Results**

The results indicated that midwives applied a form of antenatal care standards, but the way of recording was not consistent and not complete, although the manual had been provided. In addition, the data processing was done manually. Importantly, midwives were not able to identify the pregnancy risk and delivery as a result of the lack of data in the recording and reporting of antenatal care.

**Conclusions**

This study concluded that the guidelines for the managing the maternal health data already is exist, however the recording and reporting are less precise, in consequnce it cannot help midwives in the detection of risk factors accurately and promtly.

## O118 Access to screening and treatments for diabetic retinopathy under the universal coverage scheme in Thailand

### Ninutcha Paengsai^1^, Thananan Ratanachopanich^2^, On-anong Waleekhachonloet^2^, Chulaporn Limwattananon^3^, Supon Limwattananon^3^, Noppakun Thammatacharee^4^, Kanjana Sirikomol^1^, Jutatip Thungthong^1^, Kunakorn Aewsuwan^1^, Jiraphan Jaratpatthararoj^1^, Wanna Eiadprapan^1^, Amornrat Ngowabunpat^1^, Pongphan Ratchakom^1^, Wanida Jitvimonrat^1^, Jadej Thammatacharee^1^

#### ^1^National Health Security Office, Bangkok, 10210, Thailand; ^2^Faculty of Pharmacy, Mahasarakham University, Maha Sarakham, 44150, Thailand; ^3^Faculty of Pharmaceutical Sciences, KhonKaen University, Khon Kaen, 40002, Thailand; ^4^Health System Research Institute, Nonthaburi, 11000, Thailand

##### **Correspondence:** Ninutcha Paengsai (ninutcha.p@nhso.go.th)

**Background**

Diabetic retinopathy (DR), a complication of diabetes, should be screened and treated. The 5^th^ National Eye Survey in Thailand (2006-2007) reported the blindness in DR was 5.1%. Therefore, the Universal Coverage Scheme (UCS) has implemented the DR screening program and a referral system for laser or bevazicumab therapies. This study aimed to estimate current access to screening and treatments for DR.

**Materials and methods**

Outpatient visits and hospitalization data of the UCS were analyzed. The proportions of DR severity were based on published reports and used to estimate an access to the DR screening and further treatment. Descriptive statistics were used.

**Results**

There were approximately 2,389,527 adult patients with diabetes in 2015, which was 6.3% of adult UCS members. In 2014 and 2015, 114,155 (4.9%) and 105,444 (4.4%) patients had DR. It was estimated that 16.7% of them (19,064 and 17,626 patients, respectively) required for treatments. For an access to the treatments, 7,027 (36.9%) and 8,504 (48.2%) patients received laser therapy and 2,768 (14.5%) and 2,942 (16.7%) received bevacizumab therapy in the same periods. The waiting time from being diagnosed with DR until receiving the treatments was 87 days on average in both years.

**Conclusions**

Screening and access to treatments for DR in the UCS members with diabetes were modest. With the universal coverage of health insurance, there was still a room for improvement in effective coverage for ophthalmic complications of this key non-communicable, chronic diseases.

## O119 Noise control cost analysis: comparison of the installation of waste based noise barrier and the hearing protection devices usage

### Iting Shofwati^1^, Sjahrul Meizar Nasri^2^

#### ^1^Occupational Health and Safety Department, State Islamic University Syarif Hidayatullah Jakarta, Jakarta, 15412, Indonesia; ^2^Occupational Health and Safety Department, Faculty of Public Health, University of Indonesia, Jakarta, 16424, Indonesia

##### **Correspondence:** Iting Shofwati (iting_shofwati@uinjkt.ac.id)

**Background**

The selection of the noise control methods affects the effectiveness and the efficiency in reducing noise. In reality, the use of Hearing Protection Devices (HPD) is preferred than noise barrier installation. The previous study on the material development made of recycled Expanded Polystyrene (EPS) waste–called BATAFOAM-proved that it has well ability to block sound. Thus, it is suitable to be utilized as noise barrier material. This study aimed to analyze the estimated costs of BATAFOAM installation and the use of HPD in reducing noise.

**Materials and methods**

The cost effectiveness ratio was assessed by calculating the Average Cost-Effectiveness Ratio (ACER) of both noise control alternatives by looking at the costs to reduce noise each person/year and the costs to reduce noise each person/dBA.

**Results**

The cost of BATAFOAM installation to reduce noise 1 dBA/person/year was lower than the use of HPD with replacement once a month or once in 3 months. These costs were five times higher than the cost of BATAFOAM installation in reducing noise by 1 dBA.

**Conclusions**

It was proved that BATAFOAM installation is effective and efficient as a noise control alternative.

## O120 Natural antibody of IgG Anti Phenolic Glycolipid-1 is positively correlated to protective antibody of IgG Anti Epitope IDALLE ESAT-6 *Mycobacterium leprae* among household contact in leprosy endemic area

### Moch Irfan Hadi^1^, Dinar Adriaty^2^, Iswahyudi^2^, Shinzo Izumi^2^, Indropo Agusni^3^, Chatarina Umbul Wahyuni^4^, Soedjajadi Keman^4^, Jerhi Wahyu Fernanda^5^

#### ^1^Faculty of Health Sciences, State Islamic University of Sunan Ampel, Surabaya, 60237 Indonesia; ^2^Institute of Tropical Disease, Airlangga University, Surabaya, 60286, Indonesia; ^3^Faculty of Medicine, Airlangga University, Surabaya, 60131, Indonesia; ^4^Faculty of Public Health, Airlangga University, Surabaya, 60131, Indonesia; ^5^Departement of Medical Record Health Information Management, Institut Ilmu Kesehatan Bhakti Wiyata, Kediri, 64114, Indonesia

##### **Correspondence:** Moch Irfan Hadi (m_i_h@uinsby.ac.id)

**Background**

Household contact is a high risk group for transmission of leprosy. Household contact with high titers of antibody IgG anti Phenolic Glycolipid-1 has a greater risk of becoming leprosy later on. While antibody IgG anti IDALLE ESAT-6 *Mycobacterium leprae* (IDALLE L-ESAT 6) has a protective effect against subclinical stage of leprosy.

Therefore the aim of this study was to analyze the correlation of natural antibody of IgG anti Phenolic Glycolipid-1to protective antibody ofIgG anti IDALLE L-ESAT 6.

**Materials and methods**

This study was designed as an observational analitical study. The subjects of the study consisted of 95 household contacts who lives in leprosyendemic area of Lamongan Regency, East Java. Measurement of IgG anti Phenolic Glycolipid-1 and IgG antiIDALLE L-ESAT 6 levels were using by enzyme linked immunosorbent assay (ELISA) technique.

**Results**

Results showed that mean of antibody IgG anti Phenolic Glycolipid-1 level in all subjects was 134.54± 295.15Units/mL, while mean of antibody IgG antiIDALLE L-ESAT 6level was 408.61 ±Units/mL. Interestingly, there was a significant positive correlation between antibody of IgG anti Phenolic Glycolipid-1 levels and protective antibody of IgG anti IDALLE L-ESAT 6 levels (Rank-Spearman correlation test, p=0.016; r=0.247).

**Conclusions**

It is concluded that the higher natural antibodyof IgG AntiPhenolic Glycolipid-1level leads to the higher protective antibody of IgGAnti IDALLE L-ESAT 6 Level among household contacts. It means household contacts with the greater risk of becoming leprosy has the greater protective antibody against leprosy.

## O121 Estimating age of maxillary and mandibular third molar eruption of late adolescent age

### Nila Kasuma^1^, Susi Abidin Hasam^1^, Haria Fitri^1^, Fildzah Nurul Fajrin^2^

#### ^1^Department of Dentistry, Andalas University, Padang, 25128, Indonesia; ^2^Biomedical Graduate Program , Andalas University, Padang, 25128, Indonesia

##### **Correspondence:** Nila Kasuma (nilakasuma10@gmail.com)

**Background**

Third molars are the most variable teeth. They are most often congenitally missing and can follow an abortive eruption path and become impacted. The 3rd molar is the one tooth marker indicating that an individual is likely to be at least 18 years of age. The aim of this study was to predict the risk of 3rd molar eruption problems in late adolescent age as well as to find the relationship between third molar eruption and age.

**Materials and methods**

This was a cross sectional study involving a sample of 300 male students aged 16-21 and 300 female students aged 17-19. An oral examination was taken for each visual clinical subject.

**Results**

This research reveals that the mean age of having complete clinically erupted maxillary third molars was 21.02 years in male subjects and 21.48 years in female subjects. While the mean age of having complete clinically erupted mandibular third molars was 20.23 years in male subjects and 21.03 years in female subjects.

**Conclusions**

This approach requires the target individuals to have regular dental reviews or ‘checkups’, so that the status of the wisdom teeth can be monitored to prevent further pathologic conditions later in life.

## O122 The relationship of extra-familial system with sexual adolescent behavior in Padang City, Indonesia

### Vivi Triana (vivietri.76@gmail.com)

#### Faculty of Public Health, University of Andalas, Padang, 25163, Indonesia

**Background**

Unmet fulfillment of adolescent needs for achievement, conformity, find identity, popularity, and sexually needs cause adolescent easy to fall into deviant activity. One form of deviant activity is risky sexual behavior. Based on the results of the Indonesian Demographic and Health Survey 2012, there was an increase in adolescent sexual behavior (21.6%) compared to 2007 data, with pregnancy rate, abortion, and on sexual transmission disease. This study aimed to determine the relationship of extrafamilial system with teenage sexual behavior in Padang City in 2017.

**Materials and methods**

The type of research was quantitative research, with cross-sectional design. Respondents were 158 high school students in Padang City. Sampling was through multistage random sampling technique, while the number of samples was done proportionally.

**Results**

Extrafamilial system was associated with adolescent sexual behavior, where the prevalence of adolescents with high-risk sexual behaviors was 3.8 times more likely to be found in peer influences (POR = 3.8, 95% CI: 1.47-9.91) . Family system relates to adolescent sexual behavior (with 95% CI, value of communication communicationwith parent 1,97-23,48, parental supervision 1,37-7,16, family structure 1,21-8,10, family support 3,36 -77.90). Family support was a confounder for relationship of peer influences to adolescent sexual behavior, adolescents with peer influences had an opportunity to engage in high-risk sexual behavior 5.6 times compared to adolescents who did not have peer influence after being controlled by gender variables and family support.

**Conclusions**

The results showed the influence of peers had a significant relationship with teenage sexual behavior. But peer influences can be controlled by maximizing family support (parents) in the form of informational support and emotional support. It is suggested to the Education and School Office to provide counseling and understanding to teenagers and parents related to sexuality issues, so that parents can monitor teenagers association for risky sexual behavior prevention.

## O123 Public's knowledge and attitude of safety sign guide in safe behavior at railway crossings area in South of Tangerang and South of Jakarta

### Fase Badriah^1^, Najmatun Nisa^1^, Nindy Widiastuti^1^, Muhammad Farid^1^, Takeru Abe^2^

#### ^1^Faculty of Medicine and Health Sciences, Universitas Islam Negeri Syarif Hidayatullah Jakarta, Jakarta, 15412, Indonesia; ^2^Advanced Critical Care and Emergency Center, Yokohama City University Medical Center, Yokohama, 2310016, Japan

##### **Correspondence:** Fase Badriah (fase_bzm@uinjkt.ac.id)

**Background**

The high number of accidents caused by the lack of human resources railway operators, infrastructure at railway crossings, and the lack of discipline of road drivers. Safety signs at railway crossings area are important factors that affect the safety of drivers at railway crossings. This study was aimed to determine the Public’s knowledge and attitudes towards safety sign guide in safe behavior at railway crossings area.

**Materials and methods**

This study was a qualitative research. The participants were drivers, pedestrians and railway operators at four busy highway-railway crossings areas in South of Tangerang and South of Jakarta, Indonesia.

**Results**

The results showed that driver's knowledge of the safety sign guide in safe behavior at railroad crossings areas still lacking and poor. Most of drives stated that they familiar with safety sign but they did not know the meaning of all safety signs guide. This study found that many safety sign were not feasible and less strategically located.

**Conclusions**

All parties need to pay attention to the behavior of drivers in the railway crossings and improvement is needed for safety sign guide that is not feasible and put it in a more strategic place.

## O124 Effectivity of aerobic exercise toweight loss and waist circumferencedecrease in overweight adult studentsin Cimahi City, West Java, Indonesia

### Susilowati^1^, Budiman^1^, Asep Dian Abdillah^1^, Novia Arviani^1^, Kuspriyanto^2^

#### ^1^Public Health Study Programme, School of Health Sciences Jenderal A. Yani Cimahi, Cimahi City, 40521, Indonesia; ^2^Eureka Indonesia Foundation, Cimahi City, 40511**,** Indonesia

##### **Correspondence:** Susilowati (satjadibrata.susi@gmail.com)

**Background**

Indonesia has entered the top 10 rank of overweight worldwide with 19.6% (based on BMI) and 37.2% (based on waist circumference (WC)) prevalences in 2013. Weight loss is achievable with moderate intensity exercise in 30-60 minutes duration on most days of the week. This study aimed to analyze weight loss and WC decrease from nine times aerobic exercise in three weeks treatment program among overweight and obese adult students.

**Materials and methods**

Quasi-experimental design applied with one group pre and post test design. The population obtained from the screening overweight/obese in adult students. Samples were 17 students. Data were analyzed by t-test dependent and simple linear regression.

**Results**

Aerobic exercise significantly affected weight loss and WC decrease with consecutive results, weight loss mean was 2.69kg (95%CI: 2.84-3.73), WC decrease mean was 3.28 cm. Weightloss and WC decrease showed in moderate relationship with positive pattern (r= 0.35). As many as 12% of WC decrease explained by loss weight.

**Conclusions**

Moderate intensity aerobic exercise is appropriate to achieve weight loss and WC decrease in overweight or obese students.

## O125 Inhibiting factors in reducing smoking behaviour on urban poor smokers

### Suharni, Andi Asrina, Ella Andayanie

#### Faculty of Public Health, Moslem University of Indonesia, Makassar, 90231, Indonesia

##### **Correspondence:** Ella Andayanie (ella_andayanie@yahoo.com)

**Background**

The number of smokers in Indonesia increased every year and smokers are dominated by the poor. Many factors can cause smoking behavior, among others are the influence of tobacco dependency which perceived positively by smokers. The aim of this study was to analyze the inhibiting factors in reducing smoking behavior among the poor smokers in urban area.

**Materials and methods**

This was a qualitative study with descriptive approach. Fifteen informants were interviewed, they were poor people who have been smoked for a long time and worked in informal sector and lived in Mamajang District. Information was obtained through indepth interview.

**Results**

The results showed that it was very hard for poor smokers to reduce or change their smoking behavior. In this case, smoking could give comfort feeling that made them maintained the pleasure. Subjective feelings perceived by poor smokers became inhibiting factors in reducing smoking behavior.

**Conclusions**

The study concludes that the sense of comfort felt by smokers is become inhibiting factor in reducing smoking behavior.

## O126 Effect of nitrogen dioxide (NO_2_), premature birth, and low birth weight against the recurrence of asthma in children based on residential area: a retrospective cohort study

### Septia Pristi Rahmah (pristia.rahmah@gmail.com)

#### Faculty of Public Health, Andalas University, Padang, 25163, Indonesia

**Background**

One of contaminant that can cause an asthma relapse is nitrogen dioxide(NO2). The highest concentrations of NO2 in the city of Padang was found in Lubuk Kilangan sub-district and low NO2 concentration was found in the sub-district of Koto Tangah. Data of ashtma cases taken from each primary health center in respective sub-district.

**Materials and methods**

The study was conducted with retrospective cohort study design, by which NO2 exposure has occurred in the past and history of asthma relapse was followed from January to November 2014. Respondents were children ≥ 7 years old and had been suffering from asthma for at least 2 years at the time of research. The determination of sample location used spatial analysis. An assessment of dispersion towards the concentration of NO2 at each location was conducted.

**Results**

The results showed a significant relationship between the concentration of NO2 with recurrence of asthma in children (RR = 2,273; 95% CI = 1,307 –3,951). There was no difference of gender in asthma recurrence among children in this area. Low birth weight (LBW) had significant association with asthma recurrence (RR = 1,681; 95% CI = 1,004 – 2,813). It was found that the relationship between the concentration of NO2 and Asthma recurrence in children was strongly found among children with history of premature birth (RR = 1,895; 95%CI = 0,952 – 3,772). These results were obtained after controlled others variables in multivariate using Cox Regression.

**Conclusions**

Children who lived in exposed areas were at high risk of recurrence of asthma episodes than children who lived in non-exposed area. The relationship between the concentration of NO2 and Asthma recurrence in children was strongly found among children with history of premature birth.

## O127 The relationship of DPT immunization status with diphtheria based on geographic information system in Padang

### Masrizal, Febi Damisti Ramadhani

#### Faculty of Public Health, Andalas University, Padang, West Sumatera, 21528, Indonesia

##### **Correspondence:** Masrizal (masrizal_khaidir@yahoo.com)

**Background**

This study was aimed to identify the effect of covariate variables (education, knowledge, and attitude of the mother as well as the density of occupancy bedrooms) to DPT immunization status with diphtheria in children under 15 years in the city of Padang based on Geographic Information System.

**Materials and methods**

A case-control study was conducted to the population of mothers of children under 15 years in the city of Padang whereby diphtheria cases (probable cases and confirmed case) were existed. The total of 102 samples were taken by simple random sampling, matched by gender, and region of residence. Controls were the closest neighbors with the cases. Data were collected through interviews by using questionnaires. Data were analyzed by using spatial analysis.

**Results**

Bivariate analysis showed that the DPT immunization status (OR=2,42 95%CI 1,00-5,85), mother’s education level (OR=0,333 95%CI 0,12-0,9) and the density of bedrooms (OR=3,2 95% CI 1,17-8,7) were linked to diphtheria. While the knowledge and attitude of the mother did not have relationship with diphtheria and. Final multivariate modeling indicated that mother’s education, mother’s attitude, and density of bedrooms were confounders to the DPT immunization status and diphtheria.

**Conclusions**

Mother’s education, mother’s attitudes and density of bedrooms influenced the relationship status of DPT immunization and diphtheria. Thus, it is expected to the Local Public Health Center to improve health promotion about immunization and information regarding diphtheria, especially in the *Kuranji Timur*, Padang, and North Padang subdistrict.

## P1 Comparison of Accuracy of Kinyoun Gabbet Stain and Lugol Stain to detect Soil-Transmitted Helminths

### Merina Panggabean^1^, Cut Lifia Fitriani^2^, Yoan Carolina Panggabean^1^

#### ^1^Department of Parasitology, Faculty of Medicine, Universitas Sumatera Utara, Medan, 20155, Indonesia; ^2^Master Study Program Tropical Diseases, Faculty of Medicine, Universitas Sumatera Utara, Medan, 20155, Indonesia

##### **Correspondence:** Merina Panggabean (mer.pgb@gmail.com)

**Background**

In Indonesia, the most frequent helminthiases are those caused by intestinal infection with Soil-Transmitted Helminths (STH). STH includes *Ascaris lumbricoides, Trichuris trichiura*, and hookworms (*Necator americanus* and *Ancylostoma duodenale*). It can be readily diagnosed by detecting the helminthes eggs in stool samples by using microscopic techniques of Lugol stain. The most important for preventing of STH is an accurate diagnosis to prevent the transmission. The objective of this study was to compare the accurate concentration of formol-ether method with Kinyoun Gabbet stain and Lugol stain.

**Materials and methods**

A cross-sectional study was conducted on primary school children at SDN 27 Peusangan, Bireun Regency, Nagroe Aceh Darussalam (NAD) Province. The total samples obtained were 80 participants on February to June 2016. Statistical analysis was performed by Chi-square to compare accuracy between Kinyoun gabbet stain and Lugol stain.

**Results**

The result showed that sensitivity of Kinyoun gabbet stain was 84,62%, the specificity was 98,51% and its accuracy was 96,25% in detecting STH.

**Conclusions**

Kinyoun gabbet stain was more sensitive and specific in detecting Soil Transmitted Helminths compared Lugol stain in this study.

## P2 Nutritional status, physical activity, and VO_2_ max among male students in SMA Sutomo 2 Medan

### Irianto^1^, Nenni D Lubis^2^, Sri Amelia^3^

#### ^1^Student in Faculty of Medicine, Universitas Sumatera Utara, Medan, 20155, Indonesia; ^2^Department of Nutrition, Faculty of Medicine, Universitas Sumatera Utara, Medan, 20155, Indonesia; ^3^Department of Microbiology, Faculty of Medicine, Universitas Sumatera Utara, Medan, 20155, Indonesia

##### **Correspondence:** Nenni D Lubis (nenni@usu.ac.id)

**Background**

In 2013, 17.3% of Indonesian adults aged 16-18 years were obese. The effective management of obesity including diet, physical activity and behavior therapy. The purpose of this study was to determine nutritional status, physical activity and VO_2_ max among male students in SMA Sutomo 2 Medan.

**Materials and methods**

This study was descriptive analytic with a cross-sectional design. Fifty-two male students aged 15-19 years were randomly selected to participate in this research. Height was measured with stature meter and body weight was measured by weight scales. BMI was calculated and nutritional status of the students was classified based on CDC curve for age. Physical activity has been identified as the frequency of doing exercise in a week. Queens College Step Test was used to estimate VO_2_ max. The result was presented as ml oxygen per kg of body weight per minute and subsequently grouped by Bruce Protocol category.

**Results**

From 52 students, seven (13.5%) of them were found underweight, 30 (57.7%) with normal weight, twelve (23.1%) were overweight and 3 (5.8%) were obese. This study found 29 (55.8%) of all respondents had low activity and others, 23 (44.2%) had moderate and high activities. This study also found that the VO_2_ max classified as very poor to poor was 19.2% of respondents, while in good to excellent by 80.8%. BMI showed a negative correlation with VO_2_ max (r: -0.509).

**Conclusions**

This study explains that nutritional status shown by BMI affects cardio-respiratory fitness.

## P3 Factors affecting work stress of garment workers in South Tangerang villages, Indonesia

### Dini Widianti, Dian Mardhiyah, Citra Dewi

#### Public Health Department, University of Yarsi, Jakarta, 10510, Indonesia

##### **Correspondence:** Dini Widianti (dini.widianti@yarsi.ac.id)

**Background**

Work stress can be caused by work environments, excessive workload, jobs that are no longer interesting or challenging to employees, high-risk jobs, role conflict, career development, and structural organization. Village convection is a home industry area of approximately 1250 convections located in the region. Work stress can be found among workers with uncertain workdays from Monday to Sunday, no holidays depending on the number of orders, overtime to exceed 8 hours of work to pursue the target. If there are excessive orders, workers can spend hours late at night until the target orders were finished. The more materials finished, the more the workers get paid. Health services do not exist because the majority of workers are not in contract. The payroll system is done weekly. This is certainly causing stress on workers which results in decreased work productivity. This study aimed to identify factors affecting work stress among garment workers in home industry area of South Tangerang, Indonesia.

**Materials and methods**

This research is descriptive analytic research with cross sectional study method, the number of respondents 137 workers. The questionnaire used was the SDS (Survey Diagnostic Stress) questionnaire. Independent variables consist of age, education, length of work in a day, years of work, and work area. Job stressors consisting of quantitative workloads, qualitative workloads, role constraints, responsibilities to others, career development and role conflict.

**Results**

Based on the results of the research, the majority of workers (73 workers) had low stress, the production area (warehouse, pattern cutting, and sewing) caused stress by 31 times, and length of work ≤ 8 hours increased work stress 126 times.

**Conclusions**

Workers who experience low work stress can get high job stress if they don’t get further treatment. Moreover, production area and working hours ≤ 8 hours can potentially cause work stress.

## P4 E. Coli infection at a newborn celebration: food poisoning outbreak in Teluk, Indonesia

### Muhammad Syairaji (msyairaji@ugm.ac.id)

#### Department of Health Information and Service Vocational College, Gadjah Mada University, Yogyakarta, 55281, Indonesia

**Background**

On 21 November 2013, Banyumas Health Center received a notification from a healthcare that 51 people in Teluk Village suffered abdominal pain, diarrhea, nausea, vomiting, and headache after consuming food at a newborn celebration. The objective of this study was to confirm the outbreak and to identify the source of infection and causative agent.

**Materials and methods**

A case-control study was conducted. Cases were those who had eaten food at the celebration and suffered abdominal pain and/-or at least one symptom of diarrhea, nausea, vomiting, headache, or fever. Controls were people who consumed food at the celebration but remained healthy. Logistic regression was used to determine the source of illness and samples were collected for laboratory testing to identify the causative agent.

**Results**

It was found that among cases, 51% was female and 26% was 11 to 20 years old. Point source transmission was identified with the average incubation period was 7.75 hours. The significant cause was goat satay (OR = 58.43; 95% CI:5.84-584.48). Laboratory test indicated the goat satay and goat curry were contaminated by Escherichia coli.

**Conclusions**

This study confirmed an outbreak in Teluk Village. The likely source of infection was the goat meat served in two dishes. As a response to the outbreak, food hygiene counseling was conducted to people who involved in food preparation.

## P5 Environmental health risk assessment of PM10 exposure to traders in Siteba Market Area Padang

### Aria Gusti (aria.mkes@gmail.com)

#### Faculty of Public Health, University of Andalas, Padang, 25163, Indonesia

**Background**

Dust is often used as one of the indicators of air pollution. The transport sector plays an important role in air pollution. PM10 is a dangerous dust that can cause a variety of health problems, especially the increase in respiratory diseases. This study aimed to determine the level of environmental health risks through the analysis of risk of PM10 exposure to traders in the Siteba market area and risk management that can be done.

**Materials and methods**

This research used environmental health risk assessment (EHRA) method. EHRA aims to calculate the level of risk a population receives due to environmental exposure. The study was conducted from November 2016 to March 2017 with 45 respondents. Accidental sampling was used in samples selection.

**Results**

The average concentration of PM10 was 0.014 mg per kg per day. The intake lifetime and intake value of PM10 inhalation at Simpang Kodam Siteba and Simpang Perumnas Siteba have Risk Quotient (RQ) > 1, indicating that exposure is not safe for traders so control needs to be done. The intake real-time value of PM10 exposure inhaled at the three sampling sites showed that exposure was still safe or not at risk to the trader with an RQ value <1.

**Conclusions**

The calculation of lifetime risk shows that there are 2 risky sampling sites at Simpang Kodam Siteba and Simpang Perumnas Siteba with RQ > 1. This indicates that traders suffer from respiratory problems in the next 30 years.

## P6 The quality of antenatal care services in Ciputat Timur Health Center

### Desty Pratiwi Marlisman, Fajar Ariyanti

#### Department of Health Care Management, Major of Public Health Faculty of Medicine and Health Science UIN Syarif Hidayatullah Jakarta, Tangerang Selatan, 15412, Indonesia

##### **Correspondence:** Desty Pratiwi (destymar@gmail.com)

**Background**

Quality of antenatal care services (ANC) improve maternal and child health outcome. The objective of the study was to assess the quality of ANC services in Ciputat Timur Health Center.

**Materials and methods**

This was a mix-method study (qualitative and quantitative). In qualitative part, four midwives, the head of health center and head of administration officer were interviewed. In quantitative part, 32 respondents who were pregnant women had been observed during ANC from April to May 2017.

**Results**

This study showed that midwives, facilities, infrastructure and equipment met the standard requirement of ANC. Iron supplements and folic acid supplements did not fulfill the standard requirement because the stock was empty within a few days. ANC process did not meet the standard requirement because midwives did not wash hands, measure body temperature, screen tetanus immunization, provide Fe supplementation, do laboratory examination, and do maternal counseling. However, respondents were satisfied on ANC service with tangible dimensions at 98.4%, reliability by 93.6%, responsiveness by 98.51%, and assurance by 99.5%, and empathy at 99.1%.

**Conclusions**

ANC services in Ciputat Timur Health Center did not meet the standard requirements of quality ANC from Ministry of Health. The result of this study can be used as a recommendation for Ciputat Timur Health Center to improve the quality of ANC.

## P7 Risk factors of positive deviance among under-five children in fishermen families in Balanipa, Polewali Mandar district

### Dwi Santy Damayati, Nurdiyanah Syarifuddin, Syarfaini, Endang Ayu Lestary

#### Department of Public Health, Universitas Islam Negeri (UIN) Alauddin, Makassar, 92111, Indonesia

##### **Correspondence:** Dwi Santy Damayati (Santy@uin-alauddin.ac.id)

**Background**

Positive deviance explains the factors that affect the growth and good nutritional status of children living in poor or slum neighborhoods. In these area, most of children suffer from growth and development disorders with less nutritional conditions. Fishermen families are one of the marginal community groups and are subject to children exploitation due to economic and politic reasons. The purpose of this research was to determine the risk factors of positive deviance among children under-fivein fishermen families in Balanipa, Polewali Mandar District.

**Materials and methods**

This research was an observational with case-control study design. The samples were 35 under-five children with good nutritional status as cases and 35 under-five children with low nutritional status as controls. The samples were selected through purposive sampling method.

**Results**

The result showed that the pattern of care, feeding and hygiene habits, protein intake were risk factors of positive deviance among under five children in the family of fishermen. The other findings indicated that knowledge of mothers on nutrition, energy intake, history of infectious diseases were protective factors.

**Conclusions**

The pattern of care, feeding and hygiene habits, protein intake were risk factors of positive deviance whereas knowledge of mothers on nutrition, energy intake, history of infectious diseases were protective factors. This research suggests to improve community counseling program on the pattern of care, feeding and hygiene habits for under five children and to socialize positive deviance through various activities in health care services and communities.

## P8 Analysis of early intervention and detection stimulation of childhood growth and development (ECD) in primary health care of Tahtul Yaman in 2017

### Novi Berliana, Frisca Lusiana Sihotang

#### Program Studi Kesehatan Masyarakat, STIKES Harapan Ibu Jambi, Jambi, 36132, Indonesia

##### **Correspondence:** Novi Berliana (noviberliana13@yahoo.co.id)

**Background**

In 2016, among 20 primary health care centers in Tahtul Yaman District, Jambi Province, Indonesia had the lowest coverage of ECD which was about 6.49 %. This study aimed to see the ECD implementation in Tahtul Yaman in 2017

**Materials and methods**

This research was a descriptive qualitative research with in-depth interviews and FGD.

**Results**

The results showed that the implementation of ECD had not optimally been running. The input component was an executive staff of ECD with an educational history of midwifery (one-year diploma) and had followed the training. Facilities and infrastructure had not been adequate because there was no growth and developing corner. ECD implementation methods were not implemented in accordance with the manual. Process component showed that there was no specific division of tasks in training the cadre. ECD was not done at primary health center because of inadequate facilities. Executive staff had double duties and has never attended training. Furthermore, the mothers were lazy to carrying their children because they did not know information about ECD.

**Conclusions**

ECD coverage was still low due to having inadequate facilities and non-optimal organizing and implementation. The health department should do direct supervision on Tahtul Yaman. Meanwhile, Tahtul Yaman should provide counseling to the cadres and provide socialization to the mothers about ECD.

## P9 Family participation on basic sanitation in Puskesmas X Koto II, Tanah Datar District, Indonesia in 2015

### Shelvy Haria Roza (shelvyhr@gmail.com)

#### Prodi Ilmu Kesehatan Masyarakat, Stikes Syedza Saintika, Padang, 25176, Indonesia

**Background**

In developing countries, health problems often appear in terms of basic sanitation. In the work area of primary health center Puskesmas X Koto II, access to proper basic sanitation is still low or about 27.3%. This study was aimed to determine the factors associated with the family participation to basic sanitation and identifying the families’ participation model to basic sanitation in the work area of Puskesmas X Koto II in Tanah Datar District.

**Materials and methods**

The study used quantitative methods with cross-sectional design. The data was collected using a questionnaire which was spread to 95 people who work in the area of the Puskesmas. The data gathered was about the level of knowledge, attitudes, income, availability of facilities and infrastructure, support from public and medical personel, and family participation. The technique of sampling was proportional stratified random sampling. The data analysis was performed by Chi-Square test and logistic regression.

**Results**

The result found a relationship between the level of knowledge, attitude, availability of facilities and infrastructure, and the role of health personnel with public and family participation to basic sanitation. However, there was no relationship between the level of income with family participation to basic sanitation. From the results of multivariate analysis, it was identified that knowledge has the most dominant influence on family participation.

**Conclusions**

Health personnel at Puskesmas X Koto II should often provide health counseling in popular media. Tanah Datar District health office needs to socialize basic sanitation. Health authorities need to work with stakeholders who provide basic sanitation facilities and infrastructure.

## P10 Factors of length of stay in patient with diabetes mellitus type 2: analysis of electronic medical records in PKU Muhammadiyah Hospital Yogyakarta

### Ismil Khairi Lubis^1^, Susilawati^2^

#### ^1^Vocational College of Universitas Gadjah Mada, Yogyakarta, 55281, Indonesia; ^2^Puskesmas Gondokusuman 2, Yogyakarta, 55213, Indonesia

##### **Correspondence:** Ismil Khairi Lubis (ismil.khairi@mail.ugm.ac.id)

**Background**

Diabetes mellitus is one of the causes morbidity and mortality in Indonesia. Length of stay (LOS) becomes an indicator for determining the success of patient treatment. This study aimed to determine factors associated with length of stay among patients with DM type 2 in PKU Muhammadiyah Hospital in Yogyakarta, Indonesia.

**Materials and methods**

This study was an observational study. Data of all inpatient of DM type 2 admitted in 2016 were taken from the electronic medical record. The total samples were 49 patients. The variables under study were LOS, sex, age, occupational, cost source, ward class, supporting examination, informed consent and complications. Data were analyzed by using chi-square and independent sample t-test.

**Results**

The majority of patients with DM type 2 had LOS more than5 days (55.9%), were female (55.1%), 45 to 65 years (49.0%), private employment (30.6%), voluntary participant of National Health Insurance (61.2 %), third class ward (51%), supporting examination (100%), no informed consent (98%) and suffering complication (81,6%). None of these factors were related significantly with LOS due to small sample size.

**Conclusions**

This study found that patient's characteristics and the clinical condition can be used as a preliminary assessment of patients with DM type 2 to minimize the length of stay.

## P11 Climate and socio-cultural risk on the high incidence of dengue hemorrhagic fever in North Sumatera Province, Indonesia

### Fazidah A Siregar^1^, Tri Makmur^2^

#### ^1^Faculty of Public Health, University of Sumatera Utara, Medan, 20155, Indonesia; ^2^Faculty of Medicine, Islamic University of Sumatera Utara, Medan, 20155, Indonesia

##### **Correspondence:** Fazidah A Siregar (fazidah@usu.ac.id)

**Background**

Transmission of dengue virus depends on the existence of Aedes mosquitoes that are influenced by climate. Socio-cultural factor has a role on the incidence of dengue. This study determined the influence of climatic and socio-cultural factors on the incidence of dengue in North Sumatera Province, Indonesia.

**Materials and methods**

The study was a comparative cross-sectional study with 688 households both in high and low incidence district selected by using systematic random sampling. Data analysis was performed by using simple and multiple logistic regressions.

**Results**

The results revealed that length of stay, experience of DHF in a family, frequency of cleaning of water container more than 15 days and minimal practices on preventive measures as well as temperature had roles on the high incidence of dengue in North Sumatera Province.

**Conclusions**

Climatic and socio-cultural factors related to the incidence of dengue in North Sumatera Province. Health promotion activities regarding dengue prevention should be enhanced to improve the knowledge and better preventive practices among the community. Furthermore, integrating climatic factor in the national control program is necessary to strengthen the dengue control program.

## P12 Household food restriction and food access as risk factors of malnutrition in Padang Pariaman regency in 2016

### Denas Symond^1^, Erwinda^2^

#### ^1^Faculty of Public Health, Andalas University, Padang, 25163, Indonesia; ^2^Faculty of Medicine, Andalas University, Padang, 25163, Indonesia

##### **Correspondence:** Denas Symond (denaspdg@gmail.com)

**Background**

Food security is a fundamental condition for development progress and quality of life. The availability and adequacy of food will increase the productivity. The study aimed to know whether food insecurity and low food access were the risk factors of less nutrient in Padang Pariaman Regency in 2016.

**Materials and methods**

This research was analytical with a quantitative descriptive design with cross-sectional approach. This research was conducted among 75 respondents’ who were farmers and had toddler and other 75 respondents who were fishermen and have toddler in Padang Pariaman Regency. Food security was measured by 10 questions of Radimer / Cornel questionnaire, while food access was calculated by the share of food expenditure using national economic survey questionnaire.

**Results**

Most respondents had low family income in both groups (63.07% in farmers and 71.60% in fishermen). Food insecurity was more prevalent in fishermen group (72.36%) than farmer (65.67%). Non-food expenditure in fishermen group was higher than farmer group. Based on logistic regression, family nutrient intake had the strongest correlation to nutritional status.

**Conclusions**

Food insecurity and food access were not the risk factors of malnutrition, while family nutrition intake was a risk factor of malnutrition in Padang Pariaman Regency in 2016.

## P13 Soil transmitted helminthes contamination in vegetables at traditional markets in Medan City

### Talitha Letitia^1,2^, Praveena^1,2^, Yoan C Panggabean^1,2^, Merina Panggabean^1,2^, Lambok Siahaan^1,2^

#### ^1^Medical Student, Faculty of Medicine, Universitas Sumatera Utara, Medan, 20155 Indonesia; ^2^Department of Parasitology, Faculty of Medicine, Universitas Sumatera Utara, Medan, 20155, Indonesia

##### **Correspondence:** Yoan C Panggabean (carolina_yoan@yahoo.com)

**Background**

Vegetables protect the human body by providing nutrients, vitamins, minerals, protein and fibers. Otherwise, vegetables act as vehicles for the transmission of parasitic infection when contaminated as a result of various associated factors related to planting, harvesting, transportation, storage and market chain.

**Materials and methods**

In this study the contamination rate of STH were determined in two common edible vegetables including lettuce and cabbage from 25 selected traditional markets in Medan. Each of the vegetables was randomly bought from five sellers in each market. Each vegetable was put in plastic bag then labeled by name of the market and the seller and time. Each of the samples was rinsed twice with distilled water in one litrebeaker, it was decanted and centrifuged. The sediment was placed on a slide and observed under a microscope.

**Results**

The results showed that 51 lettuces (40,8%) and 44 cabbage (35,2%) were contaminated of STH. The identified STH was hookworm larvae and Ascaris *lumbricoides* egg in both vegetables. Contamination rate of hookworm larvae was 40 % in lettuce and 34,4 % in cabbage. Contamination rate of *Ascaris lumbricoides* egg was 0,8 % in lettuce. Contamination rate of Hookworm egg was 0,8% in cabbage.

**Conclusions**

Since vegetables are potential sources of transmission for intestinal parasites, consumers should always avoid acquiring parasitic infection from contaminated vegetables supplied through proper cleaning and cooking. STH contamination is found on lettuce and cabbage in selected traditional market in Medan.

## P14 Junk food and soft drink consumption as risk factors of obesity among children at elementary school in Padang City

### Rizki Adriana, Azrimaidaliza, Hafifatul Auliya Rahmy

#### Public Health Faculty, University of Andalas, Padang, 25163, Indonesia

##### **Correspondence:** Rizki Adriana (rizkiadr26@gmail.com)

**Background**

Children of elementary school are one of the risk groups that must be concerned about their nutritional status. They like to consume junk food and soft drink that may have an impact on risk of obesity. The aim of this study was to determine the relationship of junk food and soft drinks consumptions and risk of obesity among elementary school children in Padang City in the year of 2017**.**

**Materials and methods**

The study design was case-control study, and it was matched by gender and age. A total of 74 students (37 cases and 37 controls) participated in this study in the selected schools with the highest obesity prevalence in Padang City. Food intake including junk food and soft drink consumption was collected by using semi-quantitative food frequency questionnaire (SQ-FFQ). Nutritional status was measured by weight and height. Logistic Regression test was used to determine risk factors of obesity among children of the elementary school.

**Results**

There was a significant relationship between energy, carbohydrate and fat intake of junk food and soft drink with the risk of obesity. Besides, there was a significant relationship between the frequency of soft drink consumption with obesity. The energy intake of junk food and soft drink (OR 3.85), the carbohydrate intake of junk food and soft drink (OR 2,71), the fat intake of junk food (OR 4.44), and the consumption frequency of soft drink (OR 3.18) were the risk factors for obesity. The result of multivariate analysis found that fat intake from junk food and soft drink was the dominant factor with the risk of obesity among school children.

**Conclusions**

Energy, carbohydrate, and fat of junk food and soft drink consumption have a significant relationship with the risk of obesity among elementary school children We suggest schools to educate the children about healthy foods and give more focus to school canteen especially for children consumption with healthy foods and beverages.

## P15 The influence of prevention program in Dengue Hemorrhagic Fever occurence at the work area of Lubuk Buaya health center, Padang City in 2017

### Sevilla Ukhtil Huvaid, Hary Budiman

#### Public Health of Faculty, Baiturrahmah University, Padang, 25586, Indonesia

##### **Correspondence:** Sevilla Ukhtil Huvaid (sevilla_0428@yahoo.com)

**Background**

DHF is a major problem of infectious diseases in various places of the world. In Padang City throughout January of 2016, there were 100 cases of dengue fever and 4 of them died. Puskesmas Lubuk Buaya is one of the health centers with the highest dengue fever case, covering 6 urban villages where all urban villages are dengue-endemic areas because the dengue cases are always found every month. The government is continuously working to improve the DHF prevention program. But in fact, it is not easy to cope with DHF because there are various obstacles in the implementation. The purpose of this study was to identify the effect of DHF prevention programs in the number cases of dengue disease in the working area of ​​Puskesmas Lubuk Buaya Padang City Year 2017.

**Materials and methods**

The type of this research was analytic research by using cross-sectional design. The sample was 100 people who are taken through simple random sampling. The data analysis consisted of bivariate analysis using chi-square and multivariate analysis using logistic regression.

**Results**

The results showed that 13% of respondents stated that their family members experienced DHF case. Bivariate results showed that three variables had a significant relationship to the incidence of DHF including communication, resources, and disposition. Multivariate results showed that the most influential on the decrease of DHF case is the communication variable.

**Conclusions**

It is suggested to the health professionals to be able to increase cross-sectoral cooperation and to communicate behavior change through forming and increasing the active role of larva monitoring cadre. In addition, local governments also need to issue a policy in order to promote eradication of mosquito breeding activities.

## P16 Environmental risk factors of Dengue Haemorrhagic Fever in Mojokerto

### Asih Media Y, Himawan Saputra, Dwi Helynarti, Devi Wahyuni

#### Department of Public Health, STIKes Majapahit, Mojokerto, 53146, Indonesia

##### **Correspondence:** Asih Media Y (art.media79@gmail.com)

**Background**

Mojokerto District is an endemic area of DHF in Indonesia. There was an outbreak in 2015. Therefore, it is essential to know the factors that influence the transmission of Dengue virus based on geographical region. The objective of this study was to analyze the risk factors of DHF in Mojokerto.

**Materials and methods**

This study was an observational research with ecological approach. This study used secondary data in 2016. The population of this study was the whole administrative regions of Mojokerto.

**Results**

There was statistically significant association between rainfall (OR = 12, CI 95 %: 1,29-111,32) and vector population density (OR = 15, CI 95%: 1,215-185,198) with the incidence of DHF.

**Conclusions**

Risk factors for DHF outbreaks in Mojokerto District were rainfall and vector population density.

## P17 Uropathogen bacteria in urinary tract infection and antimicrobial susceptibility pattern in Haji Adam Malik Hospital, Medan, Indonesia

### Nazri Ulimaz^1^, Dian D Wahyuni^1^, Tetty A Nasution^1^, Ariyati Yosi^1^

#### ^1^Medical Faculty, University of Sumatera Utara, Medan, 20155, Indonesia; ^2^Microbiology Department, University of Sumatera Utara, Medan, 20155, Indonesia; ^3^Dermato & Venereology Department, University of Sumatera Utara, Medan, 20155, Indonesia

##### **Correspondence:** Tetty A Nasution (tetty@usu.ac.id)

**Background**

Urinary tract infection (UTI) is one of the most common bacterial infections in developing countries. The prevalence of the disease varies greatly based on the age and gender. There are several types of bacteria that cause UTI. *Escherichia coli* is the most common cause. The aim of this study was to determine the uropathogen bacteria of UTI and antimicrobial susceptibility pattern in Haji Adam Malik Hospital, Medan City, Indonesia.

**Materials and methods**

A cross-sectional study was conducted from June to December 2015. Organisms were identified from mid-stream urine samples and catheter from 110 clinically-suspected cases of UTI by using standard procedures. Antimicrobial susceptibility test was performed for the isolated pathogens using VITEK 2 (Biomerieux®).

**Results**

The most common pathogens isolated were *Escherichia coli* (42,7%), *Klebsiella pneumonia* (30,9%), *Acinetobacter baumanii* (10,0%) and *Pseudomonas aeruginosa* (6,4%). *E. coli* had the highest percentage of resistance to ampicillin and ceftriaxone (93,6%) while K. *pneumoniae* (83%) and all isolates of *A. baumanii* (100%) were resistant to ampicillin. Multidrug resistance was identified in all isolated bacteria. However, all isolates were susceptible to Amikacin.

**Conclusions**

The resistance of bacterial pathogens to the commonly prescribed drug needs routine surveillance of UTItreatment.

## P18 Bacteria identification on the air conditioner in classrooms of medical department, faculty of medicine, university of NorthSumatera in 2016

### Rahmad Diansyah^1,2,3,4^, Sri Amelia^1,2,3,4^, Nenni D Lubis^1,2,3,4^, Syah Mirsya Warli^1,2,3,4^

#### ^1^Student in Faculty of Medicine, University of North Sumatra, Medan, 20155, Indonesia; ^2^Department of Microbiology, Faculty of Medicine, University of North Sumatra, Medan, 20155, Indonesia; ^3^Department of Nutrition, Faculty of Medicine, University of North Sumatra, Medan, 20155, Indonesia; ^4^Department of Surgery, Faculty of Medicine, University of North Sumatra, Medan, 20155, Indonesia

##### **Correspondence:** Rahmad Diansyah (doktermely@yahoo.com)

**Background**

Air ventilation is an important factor in determining indoor air quality. Air Conditioner (AC) is used as an alternative of natural air ventilation. Students spend half of the day in the classroom resulting in increasing the number of pollutants in the room which may cause airborne diseases. This study aimed to identify the bacteria encountered in the AC in the classrooms of Medical Department, Faculty of Medicine USU in 2016.

**Materials and methods**

This research was a descriptive study. Bacteria were identified by using laboratory tests including culture, gram staining, and biochemical tests. The sample in this research was 26 well-functioned units of AC in the classrooms.

**Results**

The result showed that *Bacillus subtilis* bacteria were found in all AC units. These bacteria were airborne contaminants. In addition, *Klebsiella spp*. (23.08%), *Staphylococcus aureus* (19.23%), *Staphylococcus epidermidis* (11.54%), and *Escherichia coli* (3.85%) were also found. These four types of bacteria are normal flora at various places in the human body, such as skin, mouth, respiratory tract and gastrointestinal tract.

**Conclusions**

*Bacillus subtilis*, an airborne disease agent, was found in all AC units. In immunocompetent people, bacteria belonging to airborne contaminants and normal flora will not cause health problems.

## P19 Analysis of occupational accidents risk with job safety analysis among workers in ship dock area of PT. Putra Sultra Samudera of Kendari in 2016

### La Dupai, Syawal Kamiluddin Saptaputra, La Ode Firman, Yusuf Sabilu

#### Public Health Faculty, Halu Oleo University, Kendari, 93132, Indonesia

##### **Correspondence:** Syawal Kamiluddin Saptaputra (syawalkesker2012@gmail.com)

**Background**

The Job Safety Analysis (JSA) is a study method to identify hazards and potential incidents associated to develop solutions for eliminating hazard that could potentially cause occupational accidents. The purpose of this study was to know the risks in the workplace in the PT. Putra Sultra Samudera of Kendari by using JSA method, and establish control measures based on the stage of works.

**Materials and methods**

The study method was qualitative with case study approach. The informants in this study consisted of 3 key informants and 5 regular informants that directly involved in the work of boat dock.

**Results**

The analysis results of the JSA showed that in every stage of works in boat dock have different levels of risk, so that the company needs to conduct the safety briefing before doing the work as well as provide a recommendation of introduction on the potential hazards on the stage of works that could potentially be occupational accident. Based on interviews with informants, there were occupational accidents that caused death and occupational diseases. The awareness of workers about the importance of safety at work was still lack.

**Conclusions**

This study found that in every stage of works in boat dock have different levels of risk and the awareness of workers about the importance of safety at work was still lack. The company should provide the personal protective equipment especially for the handling of electricity, as well as punishment to the workers who do not use the PPE and improve the supervision at every stage of works.

## P20 The effect of therapeutic thought stopping toward anxiety in hemodialysis patients in RSAM Bukittinggi

### Yade Kurnia Sari^1^, Melia Fransiska ^2^, Fredia Heppy ^1^, Cecep Sobirin ^1^

#### ^1^Nursing Department, STIKes Prima Nusantara, Bukittinggi, West Sumatra, 26122, Indonesia; ^2^Public Health, STIKes Prima Nusantara, Bukittinggi, West Sumatra, 26122, Indonesia

##### **Correspondence:** Yade Kurnia Sari (yade_pratama@yahoo.com)

**Background**

Hemodialysis patients often experienced anxiety conditions. This is caused by 12 to15 hours of dialysis every week, or at least 3 to 4 hours’ therapies. This activity will take place continuously throughout their life. This research was aimed to determine the effect of therapy thought stopping towards anxiety among patients who undergo hemodialysis in RSAM Bukittinggi.

**Materials and methods**

The research design was quasi experimental pre- and post-test with a total sample of 64 respondents. The samples who received Therapy Thought Stopping were 32 peoples, and 32 respondents who did not get Therapy Thought Stopping. Then, HARS scale was used to determine the client's level of anxiety.

**Results**

The results showed a significant decrease in anxiety condition in the intervention group who received therapy thought stopping, compared with the group who did not get of therapy thought stopping.

**Conclusions**

Therapy Thought Stopping is recommended to clients’ hemodialysis who experience anxiety in RSAM Bukittinggi.

## P21 Analysis of friends influence with drug abuse among adolescent in Prison Class II-A Padang, 2017

### Mellia Fransiska^1^, Yade Kurnia Sari^2^, Cecep Sobirin^2^, Vivi Okta Sanggara^2^

#### ^1^Public Health Department, STIKes Prima Nusantara, Bukittinggi, 26122, Indonesia; ^2^Nurshing Department, STIKes Prima Nusantara, Bukittinggi, 26122, Indonesia

##### **Correspondence:** Mellia Fransiska (fransiska2003@gmail.com)

**Background**

National Survey on Drug Abuse in 2014 found that the prevalence of drug abuse in Indonesia was 2.18% or 3.8 million to 4.1 million people. Every day, there were 33 people died because of drugs and will increase to 5.0 million in 2020. The research objective was to analyze the influence of friends to drug abuse among adolescents in prison class II A Padang.

**Materials and methods**

This study was a combination research where quantitative method as primary research and qualitative method as secondary were employed. The total sample in this study was 42, with 21 cases and 21 controls. Qualitative data collection was conducted by in-depth interviews by which informants were selected by using purposive sampling technique. The data were analyzed with chi-square test at 95% CI.

**Results**

The results showed that the influence of friends wasa risk factor for drug abuse, and there was significant relationship between the influence of friends with drug abuse in adolescents.

**Conclusions**

It can be concluded that the influence of friends was the risk factor of drug abuse. It is recommended that parents should control their children and suggests the government to provide rehabilitation center at each hospital.

## P22 The influence of counseling on cervical cancer to the knowledge and attitude of female students at SMKN 4 and SMKN 7 Makassar, South Sulawesi province, 2017

### Masriadi^1^, Saleha Sampara^2^, Muhammad Syafar^3^

#### ^1^STIK Tamalatea, Makassar,90245, Indonesia; ^2^STIK Tamalatea, Makassar,90245, Indonesia; ^3^FKM UNHAS, Makassar, 90245, Indonesia

##### **Correspondence:** Masriadi (arimasriadi@gmail.com)

**Background**

Cervical cancer is the major cause of woman mortality due to cancer. All over the world, it is estimated of 500,000 new cancer cases and there are 250,000 deaths every year and 80% of them occurs in developing country including Indonesia. Cervical cancer is cancer with the highest prevalence in Indonesia, it was 0,8% in 2013 and similar prevalence was reported its in South Sulawesi province. This research was aimed to identify the relevance of counseling on cervical cancer on to the knowledge and attitude of female students at SMKN 4 and SMKN 7 Makassar.

**Materials and methods**

The research was a quasi experiment pre- and post-test with control group design*.* The samples were taken through simple random sampling*.* There were 94 samples involved in this study.

**Results**

The research result depicted the significant different knowledge level and respondents’ attitude on cervical cancer between treatment and the control group after the socialization was given.

**Conclusions**

This study indicates the change of knowledge and respondents’ attitude on cervical cancer illness after counseling. This research suggests the importance of consciousness for the woman on cervical cancer by taking care of themselves and prevention measures including screening and early detection.

